# Liquid Crystalline Hydroxyapatite Nanorods Orchestrate Hierarchical Bone‐Like Mineralization

**DOI:** 10.1002/smll.202310024

**Published:** 2024-08-23

**Authors:** Jishizhan Chen, Martin Birchall, Alexander J. MacRobert, Wenhui Song

**Affiliations:** ^1^ UCL Centre for Biomaterials in Surgical Reconstruction and Regeneration Department of Surgical Biotechnology Division of Surgery & Interventional Science University College London Rowland Hill Street London NW3 2PF UK; ^2^ UCL Ear Institute University College London 332 Grays Inn Road London WC1X 8EE UK; ^3^ Royal National Ear Nose and Throat and Eastman Dental Hospitals University College London Hospitals 47‐49 Huntley Street London WC1E 6DG UK

**Keywords:** hydroxyapatite nanorods, liquid crystalline structure, mineralization, osteogenesis, stem cells

## Abstract

Bone matrix exhibits exceptional mechanical properties due to its unique nanocomposite structure of type I collagen fibrils and hydroxyapatite (HAp) nanoparticles in hierarchical liquid crystalline (LC) order. However, the regeneration mechanism of this LC structure is elusive. This study investigates the role of the LC structure of HAp nanorods in guiding aligned mineralization and its underlying molecular mechanism. A unidirectionally oriented LC phase of HAp nanorods is developed through engineering‐assisted self‐assembling. This is used to study the growth direction of long‐range aligned extracellular matrix (ECM) and calcium deposit formation during the osteogenic differentiation of human bone marrow‐derived mesenchymal stem cells. It is found that 2 key regulatory genes, COL1A1 and COL4A6, lead to the formation of aligned ECM. Activation of the PI3K‐Akt pathway enhances osteogenesis and promotes ordered calcium deposits. This study provides evidence for elucidating the mechanism of LC‐induced ordered calcium deposition at hierarchical levels spanning from the molecular to macro‐scale, as well as the switch from ordered to disordered mineralization. These findings illuminate bone regeneration, contribute to the development of biomimetic artificial bone with long‐range ordered structures, and suggest a basis for therapeutic targeting of microstructure‐affected bone disorders and the broader field of cell‐ECM interactions.

## Graphical abstract

Unidirectional liquid crystalline hydroxyapatite nanorods guide the growth and osteogenesis of stem cells and the formation of ordered extracellular matrix and calcium deposits at multi‐scales. The PI3k‐Akt signaling pathway mediated by two key genes COL1A1 and COL4A6 is activated in this process. The study suggests that the liquid crystalline arrangement promotes long‐range ordered ECM and calcium deposition, reflecting natural bone formation.

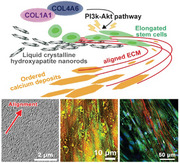

## 1 Introduction

From the first day of life, organisms engage with their surrounding environment and develop hierarchical structures. One example of such structures is liquid crystalline (LC) arrangements with long‐range orientational order.^[^ ^1^ ^]^ These organized structures are found extensively in biology, including eukaryotic cilia and flagella,^[^ ^2^ ^]^ cuticles of arthropods,^[^ ^3^ ^]^ spider^[^ ^4^ ^]^ and silkworm silk,^[^ ^5^ ^]^ mother of pearl,^[^ ^6^ ^]^ liver,^[^ ^7^ ^]^ dermis,^[^ ^8^ ^]^ tendon,^[^ ^9^ ^]^ cornea,^[^ ^10^ ^]^ and bone.^[^ ^11^ ^]^ Long‐range ordered arrangements can be observed not only in a liquid crystalline phase but also in their solidified form, resulting in unique mechanical properties. In the context of bone (Figure S1A, Supporting Information), type I collagen molecules self‐assemble into highly ordered fibrillar structures, leaving periodic minor gaps. Within these gaps, hydroxyapatite (HAp) forms deposits. The mineralized collagen molecular chains are packed into fibrils of ≈500 nm in diameter, which further aggregate into collagen fiber bundles ranging from 1 to 10 µm.^[^ ^12^ ^]^ These collagen fiber bundles then compact into dense unidirectional lamellae of bone exterior and concentric lamellae of osteons of bone interior, which can range in size from 10–500 µm. Ascenzi and Bonucci observed this chiral nematic LC‐like ordered structure at multiple scales using a polarizing microscope as early as 1968.^[^ ^13^ ^]^

Given the high demand for bone transplantation worldwide,^[^ ^14^ ^]^ artificial bone has become a prominent area of research in bone engineering and clinical treatment for bone defect repair. Numerous methods have been developed to mimic the properties of bone, including the use of alloys^[^ ^15^ ^]^ or nonmetallic materials (inorganics,^[^ ^16^ ^]^ polymers,^[^ ^17^ ^]^ composites^[^ ^18^ ^]^) and cell‐laden scaffolds composed of stem cells and natural/synthetic polymers.^[^ ^19^ ^]^ Some of those approaches incorporate anisotropic structures within scaffolds to enhance mechanical stiffness and provide topographical cues for osteogenesis.^[^ ^20^ ^]^ Although these strategies have shown promising results in terms of inducing osteogenic gene expression and enhancing calcium deposition, there is still a lack of sufficient evidence to demonstrate the attainment of long‐range ordered calcium structures at multiscale, as observed in natural bone. The key factors involved in manipulating calcium secretion patterns during bone regeneration are not well understood. The random formation and distribution of calcium pose significant challenges for achieving more controllable bone repair and improved clinical outcomes. Moreover, various diseases involve alterations in tissue microstructure. For example, bone defect malunion/non‐union can occur due to abnormal bone remodeling and the inability to form well‐organized regenerative tissues.^[^ ^21^ ^]^ Osteoporosis leads to cortical bone depletion and degenerative “tabularization”, resulting in reduced structural integrity. In cardiovascular tissues, the unexpected osteogenic differentiation of highly aligned vascular cells can cause uncontrollable calcification.^[^ ^22^ ^]^ The underlying mechanisms that regulate the transition between ordered and disordered microstructures in these contexts are still poorly understood.

Indeed, there is growing evidence suggesting that the presence of an LC‐like ordered arrangement plays a critical role in guiding the proliferation, differentiation, and secretion of cells.^[^ ^23^ ^]^ However, the specific mechanisms underlying the formation of LC‐like collagen fibrils and HAp, particularly in the context of osteogenesis, remain elusive. Wingender et al.^[^ ^24^ ^]^ found that HAp nanocrystals were embedded within LC‐ordered collagen scaffolds in vitro precursor‐based mineralization and that the preferential (001) crystallographic c‐axis of HAp aligned with the collagen fibril axis, whereas the dense LC collagen matrix exhibited local orientation. The researchers hypothesized that bone‐like nanostructure is important for guiding cell behavior, although no confirmatory in vitro study has been conducted. Another study^[^ ^24^ ^]^ utilized long‐range ordered self‐assembled collagen‐like peptide amphiphiles (CLPAs) to induce directional growth of MC3T3‐E1 cells. Subsequently, calcium phosphate precipitation was performed on the collagen‐mimetic patterns although no lattice information on the mineral deposits was reported. Some pioneering work by Kato and colleagues found that HAp LCs are capable of guiding cell alignment^[^ ^25^ ^]^ and being used as vehicles for cancer cell treatment,^[^ ^23^ ^c]^ but exploration of their properties for bone tissue engineering remains lacking. Li et al. indicated that terbium‐doped HAp nanocrystals could be utilized to track their intracellular interact with osteoblasts and offered potential applications in bone tissue engineering.^[^ ^26^ ^]^

Here, we investigated the formation of directionally ordered calcium deposits at multiple scales during osteogenic differentiation of human bone marrow‐derived stem cells (hBMSCs) using a unidirectional LC structured template that was self‐assembled from fluorescently trackable HAp nanorods (NRs) colloid. Through RNA sequencing (RNA‐seq) and bioinformatic technologies, we explored the hub genes and signaling pathways involved in interactions between cells, LC structures, and HAp NRs. The results further elucidated the molecular mechanisms underlying anisotropic calcium deposition and revealed the principles controlling ordered bone regeneration. Our findings have implications for guiding the design and fabrication of biomaterials, regenerative artificial bone scaffolds and therapeutic approaches aimed at promoting ordered bone regeneration.

## 2 Results and Discussion

### 2.1 Liquid Crystalline HAp Nanorod (NR) Colloids and Characterization

**Figure 1:**
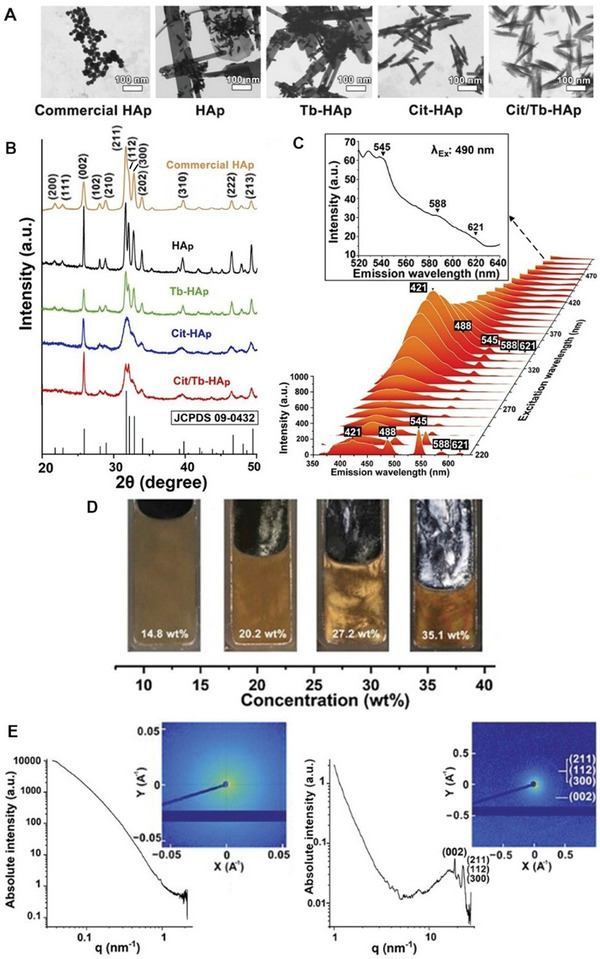
Formation and characterization of hydroxyapatite nanorod liquid crystals (Cit/Tb‐HAp NR LC). A) Low magnification STEM images (Scale bar = 100 nm) and B) XRD spectra of synthesized and commercial HAps nanoparticles. C) 3D waterfall plot of full excitation/emission spectra of Cit/Tb‐HAp. The excitation wavelength of 340 nm excites the highest emission peaks. The magnified Figure shows that even when the excitation wavelength moves to 488 nm (a frequently used excitation wavelength on fluorescence microscopes), the terbium peaks at 545, 588, and 621 nm are still recognizable. D) Formation of lyotropic liquid crystalline Cit/Tb‐HAp aqueous colloids. E) 1D spectra and 2D patterns of SAXS (left) and WAXS (right) of LC Cit/Tb‐HAp colloid in a capillary. No significant peaks corresponding to the long‐range ordered structure of Cit/Tb‐HAp LC are found at low q values apart from sharp peaks at high *q* values corresponding to HAp NR crystal structure.

To generate unidirectionally aligned LC structures, fluorescently trackable anisotropic HAp NRs with terbium doping, named Cit/Tb‐HAp, were synthesized via hydrothermal methods (**Figure** **1**A; Figure S1B, Supporting Information) described by Jin et al.^[^ ^27^ ^]^ and Yang et al.^[^ ^28^ ^]^ with minor revisions. The introduction of carboxyl bonds on the HAp surface enhanced the negative charge and repulsive force, which allowed for the formation of stable colloids of Cit/Tb‐HAp NRs in the LC phase at a suitable concentration. For comparison, pure HAp, Tb‐HAp without citrate, and Cit‐HAp samples were also synthesized. Commercial HAp was used as a positive control (see Supplementary Materials and Methods for details). The chemical and physical structures of the samples were characterized using various techniques. FTIR spectroscopy (Figure S2, Supporting Information) showed similar characteristic absorption peaks for the lyophilized powders of commercial HAp, HAp, Tb‐HAp, Cit‐HAp, and Cit/Tb‐HAp, corresponding to specific vibrations of chemical bonds in functional groups such as PO_4_^3−^, OH^−^, and CO_3_^2−^.

The crystal structure of the HAp samples was characterized using X‐ray diffraction (XRD). All the samples exhibited characteristic diffraction peaks corresponding to the HAp crystal structure (Figure 1B). Comparing the diffraction peaks to the standard data for hexagonal HAp (JCPDS No. 09–0432), peaks were observed at 2θ = 25.9°, 28.1°, 29.0°, 31.8°, 32.9°, 34.0°, 39.8°, 46.7°, and 49.5°, corresponding to the lattice planes (002), (102), (210), (211), (300), (202), (310), (222), and (213) respectively. The HAp sample showed sharp and well‐separated peaks, while the Tb‐HAp sample exhibited fewer but still well‐separated peaks, indicating increased lattice disorder due to Tb doping. The Cit‐HAp and Cit/Tb‐HAp samples displayed partially convolved peaks, especially for the (211), (112), and (300) planes, suggesting that citrate had a negative effect on the degree of crystallinity. The crystallinity index (CI) was calculated using the integral area method,^[^ ^29^ ^]^ and all synthesized HAp samples showed high crystallinity ranging from 88.5% to 92.7% (Table S1, Supporting Information). In comparison, the (CI)_XRD_ calculated using peak intensity^[^ ^30^ ^]^ was lower, with values of ≈0.64, 1.25, 0.91, 0.34, and 0.56, commercial HAp, HAp, Tb‐HAp, Cit‐HAp, and Cit/Tb‐HAp, respectively (Table S1, Supporting Information). From the JCPDS database, well‐crystallized HAp has a (CI)_XRD_ of 1.45. It is worthwhile to compare synthesized HAp with natural human bone. When using the same calculation of (CI)_XRD_ by peak intensity, the (CI)_XRD_ of human bone is reported with a wide range from 0.08 to 1.21^[^ ^30^, ^31^ ^]^ due to difficulties in fully removing organic components and differences among individuals, or types of bone. Even so, it is known that several forms of apatite (mostly HAp) comprise inorganics in fresh bone and have low crystallinity,^[^ ^32^ ^]^ which is similar to the HAp synthesized here. To assess the preferred orientation of HAp, the “distance” to R_hkl_ (DtR, see Methods for explanation) was calculated. The (200) and (300) planes correspond to the α‐axis while the (002) plane reflects the c‐axis of HAp. The DtR values for the (200), (300), and (002) planes were compared, and it was found that all synthesized HAp samples had significantly greater DtR_(002)_ values than DtR_(200)_ and DtR_(300)_. This indicates the growth direction (c‐axis) of HAp NRs (Figure S3, Supporting Information), regardless of terbium or trisodium citrate doping. It is of note that the commercial HAps have low DtR on all of the (200), (300), and (002) planes.

Scanning transmission electron microscopy (STEM) images (Figure 1A; Figure S4 and Table S2, Supporting Information) were used to measure the particle size. A rod‐like morphology of Cit/Tb‐HAp with a high aspect ratio (length/diameter, L/D) was observed. Successful Tb doping was confirmed through fluorescence tests (Figure 1C; Figure S5, Supporting Information) and EDX analysis and mapping (Figure S4 and Table S3, Supporting Information). It is noted that EDX may not precisely determine the composition of a bulk sample as it is a surface characterization technique with a scanning depth of ∼1 µm. Fluorescence spectroscopy of Cit/Tb‐HAp exhibited intense emission peaks at 421 or 430 nm, which can be attributed to the carbon dots (sourced from trisodium citrate) that are trapped within the Cit/Tb‐HAp crystals,^[^ ^33^ ^]^ and 4 characteristic fluorescence peaks of terbium were observed at 488, 545, 588, and 621 nm.

**Figure 2:**
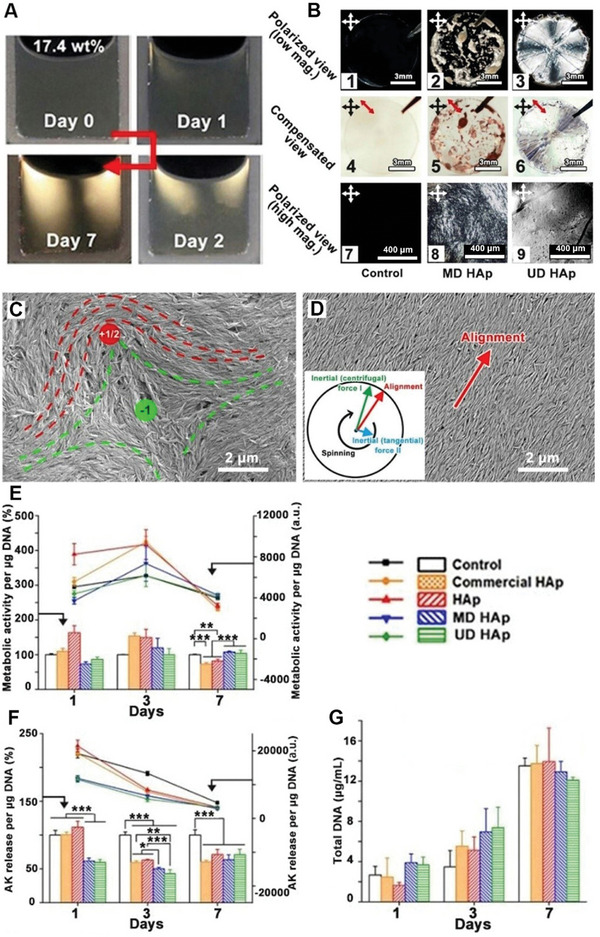
Control multiple to the unidirectional orientation of Cit/Tb‐HAp NR LC and cellular responses. **A**) Growth of birefringent domains of HAp NR colloid (17.4 wt.%) in a sealed cuvette over time. **B**) Cross‐polarized micrographs of HAp NR LCs with multi‐domains (MD) and radially unidirectional orientation (UD) (1 – 3) and compensated views (4–6) with a first red compensator (1/4λ wavelength, red arrows) at 45° to the analyzer at low magnification, and (7–9) at high magnification (7–9). **C**) The FE‐SEM image of MD HAp NRs. The dash lines highlight a typical +1/2 (red) and a −1 (green) disclinations. Scale bar = 2 µm. **D**) The FE‐SEM image of UD HAp NRs. The red arrow indicates the direction of alignment. An insert image at the left corner displays the alignment orientation (red) of Cit/Tb‐HAp NRs as the result of inertial (centrifugal) force I (green) and inertial (tangential) force II (blue) during spin coating. Scale bar = 2 µm. **E**) The metabolic activity of hBMSCs proliferation on HAp substrates. **F**) The concentration of adenylate kinase (AK) released into the medium. **G**) Total DNA of hBMSCs. All values are recorded on days 1, 3, and 7 of proliferation. In **E)** and **F)**, the bar charts and the left y‐axis represent the relative percentage of change compared to the control group, while the line charts and the right axis are averaged by the total DNA. Each condition *n* = 3. Statistical analysis: one‐way ANOVA. *: *p *< 0.05; **: *p *< 0.01; ***: *p *< 0.001.

As anticipated, both Cit‐HAp and Cit/Tb‐HAp NR colloids self‐assembled into a lyotropic liquid crystalline phase upon reaching a concentration of 19.8 and 20.2 wt.% in short‐term stationary conditions (one day), respectively. This mesophase arises from the anisotropic interactions of the uniform rod‐shaped particles. To characterize the LC state of the Cit/Tb‐HAp dispersion, a series of examinations were conducted using a cross‐polarized light set‐up. In Figure 1D, Cit/Tb‐HAp at low concentration (14.8 wt%) looked translucent with minimal birefringence texture. However, as the concentration increased to 20.2 wt%, weak birefringence became noticeable. The birefringence domains expanded significantly and intensified throughout the entire sample as the concentration reached 27.2 wt.%. Further increased concentration (above 35.1 wt.%) led to the organization of more colorful and denser birefringence domains. The observed phase transition is similar to the previous report for Cit‐HAp dispersion.^[^ ^34^ ^]^ This optical evidence, along with the observation of diffuse scattering in the small angle X‐ray scattering (SAXS) pattern and spectrum (Figure 1E left) and morphology revealed by field emission scanning electron microscopy (FE‐SEM) (Figure 2C,D), confirms that the Cit/Tb‐HAp NR LC (as observed WAXS in Figure 1E right) is in the nematic phase with 1D long‐range directional order, similar to the characteristic features of nematic phase of small molecular and polymer liquid crystals. It is worth noting that no distinct biphase birefringent texture of the isotropic‐nematic transient phase was apparent and that no significant peaks at lower *q* values of SAXS 1D spectra and 2D patterns corresponding to the interparticle distance in the long‐ and short‐axis distance were observed in Cit‐Tb‐HAp NR LC (Figure 1E), different from those reported in PAA/HAp hybrid LCs.[48,49] These observations prove that Cit‐Tb‐HAp NRs are capable of organizing themselves locally into nematic ordered structures along the long‐axis in a progressive manner but tend not to pack themselves as closely and uniformly as PAA/HAp hybrid LCs, which may be attributed to the higher aspect ratio and greater polydispersity in their length and diameter (Table S4 in Supporting Information).

### 2.2 Unidirectional HAp NR LCs and Biocompatibility

Like other nanoparticle LCs and small molecular and polymeric LCs, the nematic texture of HAp NR LCs under cross‐polarized light and microscope (Figure 1E and **Figure** **2**A) is an optical effect resulting from a locally ordered multi‐domain (MD) microstructure consisting of orientational singularities, known as disclinations.^[^ ^35^ ^]^ The density of disclinations tends to decrease over time due to their annihilation during self‐assembling or under external forces.^[^ ^36^ ^]^ In Figure 2A and Figure S6 (Supporting Information), the formation of large birefringence domains under surface tension in the cuvette was observed, leading to a decrease in dislocation density. When the Cit/Tb‐HAp was firmly sealed in a cuvette at 17.4 wt.%, although no typical nematic birefringent texture was initially observed during preparation, the growth of brighter birefringence domains was observed over time (7 days), while the colloid volume remained almost unchanged (Figure 2A). This indicates that more ordered nanorods (NRs) merged into the larger uniform domain. This slow growth of nematic ordered domains at a concentration lower than 20.2 wt.% suggests that the actual critical concentration of isotropic‐nematic transition may be lower than that was observed in the short‐term stationary condition. Figure S6 (Supporting Information) demonstrates the exchange of bright and dark domains when the sample is rotated every 45° to the crossed analyzers, indicating a large and uniform alignment.

It is also well known that LC phase can facilitate large‐scale alignment during processing, owing to its shear thinning effect. This effect allows for the uniform orientation of nanoparticles or molecules under relatively low shear stress, such as Kevlar fibers made from LC phase. To eliminate disclinations and achieve uni‐directional (UD) alignment of HAp NRs similar to the bone lamellar matrix, a spin coating technique was applied. In Figure 2B, under cross‐polarized light at both low and high magnifications, intense birefringent textures are observed only in the MD and UD HAp groups. The dense Schlieren texture of MD HAp NRs (Figure 2B2) indicates a random distribution of locally ordered polydomain. On the other hand, the UD HAp group (Figure 2B3) shows 4 extinction black “brushes” crossing each other, originating from the spin center. The presence of a single four‐brush Schlieren texture across the whole view of the UD HAp NRs sample, resembling a “Maltese cross” pattern of polymer spherulites under cross‐polarized light, indicates uniform alignment at the macro‐scale. The polarized views of commercial HAp and HAp groups are shown in Figure S7 (Supporting Information). By inserting a 1/4 λ compensator at 45°, the MD HAp NRs exhibit a random distribution of yellow and blue domains (Figure 2B5), while in the UD HAp group, the interference yellow color in the second and fourth quadrants and the blue color in the first and third quadrants are exhibited (Figure  2B6) which indicates that the refractive index of the UD HAp LC in the radial direction (fast ray vibration) is larger than that in the circumferential direction (slow ray vibration).

The FE‐SEM morphological image provides further insight into the organization of HAp NRs in the LC phase. In Figure 2C, the local orientation of HAp NRs ≈+1/2 and −1 disclinations, which are close to each other, is observed in the MD HAp group. The MD HAp groups are primarily composed of ±1/2 disclinations that are randomly distributed, contributing to a wide range of orientation distribution (Figure S8A, Supporting Information). In contrast, it is evident that the long axis of UD HAp NRs is well aligned radially out of the spin center, primarily influenced by the frictional drag induced by centrifugal force and the induced laminar shear flow during spin coating (Figure 2D insert). As a result, the spin coating technique led to a narrower angle distribution over a longer distance across the entire sample (Figure S8B, Supporting Information). This demonstrates the effectiveness of spin coating as a method for achieving particle alignment at the macro‐scale.

**Figure 3:**
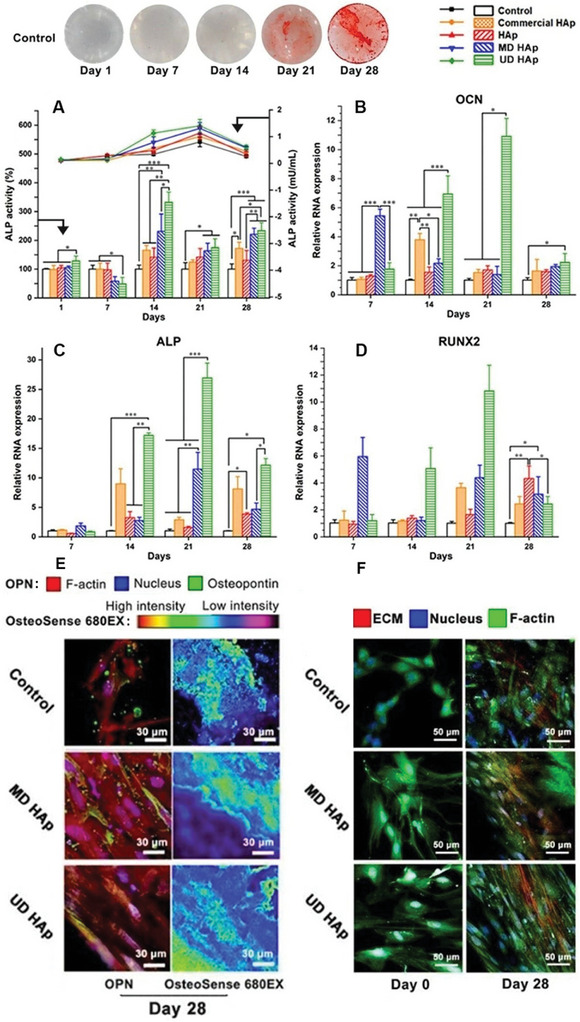
HAp liquid crystalline structure guiding osteogenic differentiation of hBMSCs. On the top is the increasing Alizarin Red S staining of calcium deposit, showing the control group underwent osteogenic differentiation. Round coverslip diameter = 9 mm. A) ALP activity of hBMSCs during osteogenic differentiation cultured on different HAp substrates on days 1, 7, 14, 21, and 28. The bar charts and the left y‐axis represent the relative percentage of change compared to the control group, while the line charts and the right axis represent absolute values. B–D) OCN, ALP, and RUNX2 gene expression in hBMSCs during osteogenic differentiation cultured on HAp substrates on days 7, 14, 21, and 28. Each condition *n* = 3. Statistical analysis: one‐way ANOVA. *: *p *< 0.05; **: *p *< 0.01; ***: *p *< 0.001. E) Confocal microscopy images of expression of osteopontin (OPN staining in green) and calcium deposits (scaled in the spectrum from red to black stained by OsteoSense 680EX) of hBMSCs on different HAp substrates on day 28. Captured using a 63× oil objective. Scale bar = 30 µm. F) Multiphoton microscopy visualization of hBMSCs and ECM during osteogenesis on different HAp substrates on days 0 and 28. Highly aligned ECM is observed in the UD HAp group. Red: ECM; blue: nucleus; green: F‐actin. Scale bar = 50 µm.

To ensure the suitability of HAp substrate for hBMSC differentiation, cell viability tests were conducted by seeding hBMSCs on HAp substrates and culturing them in the proliferation medium for 7 days. A metabolic test (Figure 2E) shows that the metabolism in all HAp‐coated groups underwent a significant surge in the early stages (day 3), followed by significantly higher metabolic activity in the HAp LC groups, both with MD or UD, compared to the non‐LC HAp and control groups at the later time point (day 7). Cytotoxicity testing (Figure 2F) demonstrated that hBMSCs in all HAp‐coated groups released significantly fewer apoptosis proteins at all time points compared with the control group. The total DNA test (Figure 2G) indicates that all groups exhibited a similar proliferation rate, with no statistical difference observed across the proliferation culture period. By the end of the 7‐day proliferation period, the total DNA content had increased four‐fold. The biocompatibility test suggests that HAp substrates have a negligible influence on the proliferation of hBMSCs. Consequently, all the tested groups are deemed suitable for subsequent differentiation testing. Both MD and UD HAp could induce elongated cell morphology. The main difference between them is the scale of the consistent orientation of elongation (Figure S9, Supporting Information) and osteogenic differentiation of hBMSCs in the following section (Figure 3).

### 2.3 Liquid Crystalline HAp NRs Guide Osteogenic Differentiation of hBMSCs

During the 28‐day osteogenic differentiation of hBMSCs (pluripotency tests see Figures S10 and S11, Supporting Information) on different HAp substrates, various methods were applied to assess the efficiency of osteogenesis at the gene and protein levels. ALP activity and real‐time PCR were performed to measure osteogenic markers. The results (**Figure** **3**A) showed that ALP activity peaked on day 21 in all groups, with the highest relative difference observed between the control and the UD HAp groups on day 14 (*p* < 0.001). The expression of osteogenic‐related genes (OCN, ALP, and RUNX2) in the MD HAp group is higher than the UD HAp group on day 7. This could be a result of more attachment points and a random local orientation of multi‐domain surface provided by MD HAp, thereby benefitting initial cell attachment and gene expression. On the other hand, more stresses involved in the attachment, migration, and alignment of the cells on UD HAP inhibited osteogenesis. However, with the passage of time, the unique topography of UD HAp led to significantly higher osteogenic gene expression than the other groups (Figure 3B–D), peaking on day 21 and decreasing thereafter. The calcium deposit was detected using OsteoSense 680EX on day 14, with the highest deposition on the UD HAp NR LC group (Figure 3E, Figure S12, Supporting Information). The intensity of calcium fluorescence and the staining of Osteopontin (OPN) increased in all groups throughout the osteogenic differentiation period. The growth trends of calcium deposition and OPN calcium fluorescence and the expressions of osteogenic‐related genes were consistent. The element ratio and kinetics of calcium deposits were also studied, revealing a dynamic pattern of how hBMSCs utilized Ca^2+^ from the medium and substrates. Chemical element analysis via EDX (Table S5, Supporting Information) showed that all groups had calcium‐defective deposits with Ca/P <1.67, similar to native bone. The Tb‐doped HAp groups (HAp, MD, and UD HAp NRs) exhibited enriched Tb element in deposits after osteogenic differentiation. Calcium ion kinetics (Figure S13A, Supporting Information) demonstrated that hBMSCs initially consumed soluble Ca^2+^ from the medium, (Figure S13B,C, Supporting Information), while the HAp substrates absorbed Ca^2+^ until saturation (Figure S13D–G, Supporting Information), becoming the major source of calcium after 21 days.

The morphology of hBMSCs and the distribution of focal adhesions (FAs) reflect tension on the cytoskeleton,^[^ ^37^ ^]^ and was used to investigate the cell‐LC interaction. In the UD HAp group, hBMSCs exhibited an elongated morphology and the ECM showed high alignment along the long axis of the cell body during osteogenesis in the MD and UD HAp groups (Figure 3F). X‐ray photoelectron spectroscopy (XPS) analysis indicated increased ECM adhesion to the HAp substrate and calcium deposits on day 28 (Figure S14, Supporting Information). The distribution of FA in the MD and UD HAp groups differed from the random patterns observed in other groups. FAs in these groups were predominantly located at the 2 ends of the elongated cell body (Figure S15, Supporting Information), suggesting that the hBMSCs on the MD and UD HAp NRs experienced directional high tension on the cytoskeleton. These findings confirm that the UD HAp NRs facilitate osteogenesis to the greatest extent, guiding cell growth in a specific morphology and direction, as well as promoting mineral and matrix secretion at the microscale.

### 2.4 Verification of Long‐Range Ordered Calcium Deposits

**Figure 4:**
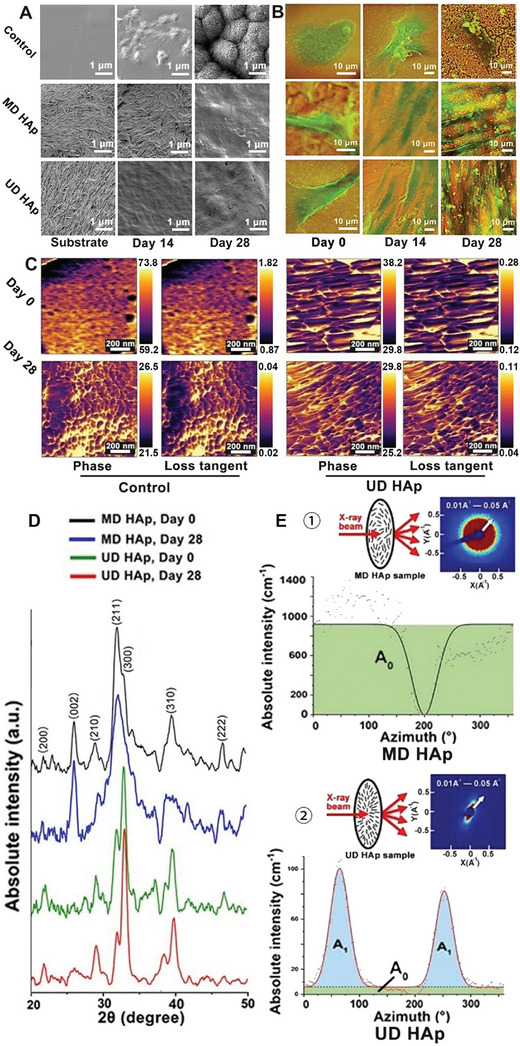
Characterization of the structure of calcium deposits at multiple scales. A) Field emission scanning electron micrographs (FE‐SEM) of the morphology of the HAp substrates and calcium deposits secreted by hBMSCs on days 0, 14, and 28. Scale bar = 1 µm. B) Density‐dependent color scanning electron micrographs (DDC‐SEM) of hBMSC morphology (green) gradually covered by dense calcium deposits (red) on PDMS (control) and different HAp substrates on days 0, 14, and 28. Scale bar = 10 µm. C) The phase and loss tangent images by AFM scanning (Scale bar = 200 nm), D) XRD spectra (days 0 and 28), and E) SAXS 2D patterns and analysis of calcium deposits in the MD and UD HAp groups (day 28) of osteogenic differentiation. ① and ② SAXS 2D patterns and corresponding 1D azimuth plots. The white arrows indicate the *q* range (*q* = 0.01 Å^−1^ to 0.05 Å^−1^). The upper schematic illustrates that the samples are positioned perpendicularly to the incident X‐ray beam. In the 1D azimuth plots, black curves: the MD HAp groups; red curves: the UD HAp group; the green area: is A_0_, the area below the baseline; the blue area: is A_1_, the area above the baseline.

The main objective of this study was to investigate whether UD HAp NR LC can induce the formation of an ordered calcium deposit structure from differentiated hBMSCs and to identify the associated signaling pathway. The morphology of the cells and calcium deposits was analyzed using FE‐SEM and density‐dependent color scanning electron micrograph (DDC‐SEM) techniques. FE‐SEM images (**Figure** **4**A; Figure S16, Supporting Information) show that in the control groups, nano‐particle‐like calcium deposits were initially observed from day 14. As the differentiation time increased, more calcium deposits formed individually on the uneven multi‐domain‐like cells, resulting in a rough grainy surface by day 28 in both the control and random HAp groups. However, in the MD and UD HAp groups, the presence of calcium deposit particles was scarcely observed. Instead, a smooth and continuous layer of calcium deposits covered the surface of the UD HAp NR LC on day 14, which transformed into densely packed lamellae aligned in a specific direction by day 28. On the MD HAp NR LC surface, the growth of the calcium deposit layer appeared relatively slower, eventually forming a densely packed surface with fine granules embedded in the ECM by day 28. These findings indicate that on the surface of the HAp NR LC, calcium deposits are guided to grow and integrate with cells and the newly formed ECM.

The composition and topology of the sample surface were further examined using DDC‐SEM micrographs, which integrate secondary and backscattered electrons in the z‐direction. In the DDC‐SEM images (Figure 4B; Figure S17, Supporting Information), the orange color represents calcium deposition (denser material), while the green color represents differentiated cells and the extracellular matrix generated (less dense material). The hBMSCs in the MD and UD HAp groups showed elongated morphology and large‐scale alignment starting from day 14, consistent with the immunohistochemical images of cell morphology shown in Figure 3E,F. In the MD HAp group, cell alignment was observed in local areas, while the hBMSCs in the UD HAp group, hBMSCs followed the global uniaxial orientation of HAp NRs. These alignments became more pronounced as the differentiation culture continued, eventually leading to highly aligned differentiated hBMSCs in both MD and UD HAp groups by day 28. In contrast, hBMSCs in other groups displayed randomly spread morphology throughout the entire culture period, similar to the control group. The DDC‐SEM images also revealed the distribution of calcium deposits. A uniform and dense layer of calcium deposit gradually formed underneath or between highly aligned hBMSCs from day 14 and it became more integrated and embedded with the aligned differentiated cells and ECM generated by day 28 in both MD HAp and UD HAp groups. This is consistent with the fluorescence images showing a continuous layer of calcium deposit in Figure 3E and fine and smooth morphology showed in FE‐SEM images in Figure 4A. In contrast, the control and non‐LC HAp groups exhibited separate clusters of calcium deposits scattered on the surface or bottom of the differentiated cells (Figure S17, Supporting Information), which correspond to the rough and grainy morphology observed in the FE‐SEM images in Figure 4A and fluorescence images in Figure 3E and Figure S12 (Supporting Information). This morphology of random clusters of calcium deposits may be the characteristic type in accordance with previous reports in the literature. For example, Bertazzo et al.^[^ ^38^ ^]^ described spherical calcium phosphate particles found on human calcific cardiovascular tissues.

An atomic force microscope (AFM) was used to analyze the surface structure of calcium deposits at the nanoscale. The height and phase images obtained from AFM (Figure 4C; Figure S18, Supporting Information) revealed aligned nanosheet‐like calcium deposits in both MD and UD groups on day 28. In contrast, the control and random HAp groups exhibited irregular granule‐like nanoparticles (50–100 nm). The phase image, together with dynamic measurement, generated the loss tangent image that showed bright boundaries between particles (particle gaps, day 0) or between calcium deposits, (ingrown ECM, day 28). The broad boundaries observed on day 0 indicated loose particle packing, while finer and aligned boundaries on day 28 indicated compact packing of calcium deposit nanosheet‐like minerals by the ECM in the UD HAp group, resembling the structure of natural bone. Notably, the grain‐grain distance in the UD HAp group on day 28 was calculated to be ≈36.8 nm, which strongly supports the lamellar distance of 36.4 nm measured by STEM (Figure S19C3, Supporting Information) (See caption of Figure S18 (Supporting Information) for detailed discussion about AFM results). The surface modulus of newly‐formed calcium deposits was also calculated, showing slightly softer than the HAp substrates. This could be attributable to the larger interface area between the nanosheets (smaller than HAp NRs) and the ingrowth of ECM as matrix, compared to the HAp NR substrate alone.

High‐resolution transmission electron microscopy (HRTEM) in Figure S20 (Supporting Information) provided more insight into the lattice structure of calcium deposits. In the control, MD HAp, and UD HAp groups, well‐crystallized polycrystals were observed. Interestingly, more lattice grain boundaries were found in the control and MD HAp groups, while the UD HAp group shows uniform lattice domains with fewer grain boundaries. These findings support the hypotheses of biosynthesized hydroxyapatite (BHAp). More discussion about HRTEM analyses can be seen in Note S1 (Supporting Information). Confocal Raman spectra and 2D microscopic images confirmed the characteristic scattering peak at 962 cm^−1^ corresponding to the symmetrical stretching vibration of phosphate calcium deposits. However, this peak was weaker and noisier compared to the HAp NR substrate reference (Figure S21A, Supporting Information). The combined results confirmed the occurrence of ordered mineralization and the generation of new calcium deposits that were assembled into elongated differentiated cells and aligned ECM on the UD HAp NRs during osteogenesis differentiation.

The alignment of calcium deposits at the macroscale was investigated through a comparison of XRD and SAXS analyses before and after osteogenesis in the presence of HAp NR substrate. XRD analysis (Figure 4D) revealed subtle changes in HAp crystal structure after calcium deposits formed on the HAp NR LC. In the MD HAp group, the relative intensity of peaks remains similar but the (211) and (200)/(300) peaks exhibited broadening on day 28, suggesting the presence of fine polycrystals. In the UD HAp group, the (002) plane was not detected on day 0, indicating that the spin‐coated HAp NRs were uniformly oriented, with the preferred (002) planes parallel to the incident beam. The (200)/(300) planes perpendicular to (002) and the incident beam exhibited higher I_(200)_: I_(211)_ and I_(300)_: I_(211)_ ratios (0.36 and 1.73, respectively) compared to the theoretic ratio (0.1 and 0.60, respectively). On day 28, the (002) plane is still not observed while I_(200)_: I_(211)_ and I_(300)_: I_(211)_ ratios further increased to 0.43 and 2.89, respectively. This indicates that the calcium deposits are also ordered crystals with the (002) planes perpendicular to that of the UD HAp and mainly stacked along the (300) direction. The different mineralization processes are depicted in Figure S22 (Supporting Information).

SAXS analysis of the UD HAp group (Figure 4E1,E2; Figure S23, Supporting Information) shows polarizing patterns on days 0 and 28. A broad shoulder peak at ≈0.28 nm^−1^ on day 0 corresponds to the distance (d‐spacing = 22.4 nm) between HAp NRs in the direction of the short axis. On day 28, the polarizing pattern remained strong and became sharper at both ends, indicating an increase in alignment from *φ* = 65.0% to 77.2% (Figure 4E2). In contrast, the MD HAp group exhibited random distribution with *φ* = 0.0% at all times (Figure 4E1; Figure S23A, Supporting Information). The 2D pattern and azimuth plot further confirmed the increased alignment in the UD HAp group after osteogenesis, likely due to the newly formed calcium deposits growing along the same orientation of the UD HAp NRs. It is worth mentioning that no new peak appeared in the 1D plot of the UD HAps NRs on day 28 (Figure S24, Supporting Information) as expected from the *d*‐spacing of repetitive lamellar structure (36.4 nm, measured by STEM image in Figure S19C3, corresponding to *q* = ≈0.18 nm^−1^). This could be due to the main broad peak from HAp NRs overshadowing any possible new peak generated by the calcium deposits.

In summary, the evidence strongly suggests that the UD HAp NR LC guides the formation of calcium deposits with anisotropically ordered structures from nano‐ to macro‐scales.

### 2.5 Molecular Mechanism

To understand the interaction between cells and the LC at the gene level, RNA sequencing (RNA‐Seq) and bioinformatic analysis (see Note S2, Supporting Information for details) were conducted, followed by verification through Western blot and PCR. The RNA‐Seq analysis identified a total of 259 and 220 Database of Essential Genes (DEGs) in the MD and UD HAp groups, respectively. The overlapped DEGs between the 2 groups were attributed to similarities in composition, stiffness, and roughness, while the difference in HAp alignment range contributed to distinct DEGs. For further analysis, the focus was placed on the UD HAp group.

**Figure 5:**
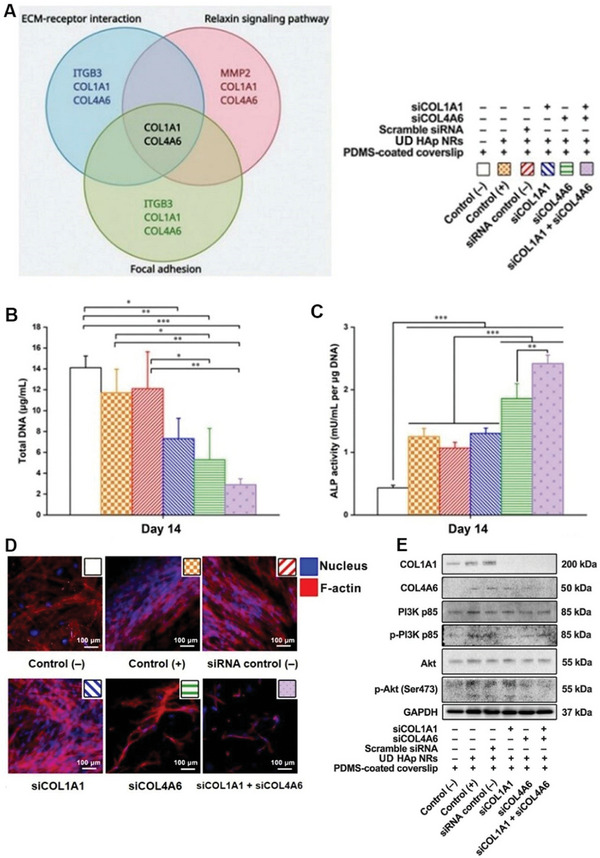
hBMSC‐LC‐structure interaction and the underlying molecular mechanism. A) Venn diagram of hub genes and signaling pathways. Notably, COL1A1 and COL4A6 are involved in all 3 enriched pathways. B) Total DNA of hBMSCs with different treatments on day 14 of osteogenic differentiation. C) Average ALP activity of hBMSCs with different treatments on day 14 of osteogenic differentiation. D) Morphology of hBMSCs with different treatments. Blue: nucleus; red: F‐actin. Scale bar = 100 µm. E) Western blot of COL1A1, COL4A6, PI3K, phospho‐PI3K (p‐PI3K), Akt, phospho‐Akt (p‐Akt), and GAPDH on day 21 of osteogenic differentiation. For uncropped membranes, see Figure S35 (Supporting Information). Each condition *n* = 3. Statistical analysis: one‐way ANOVA. *: *p* < 0.05; **: *p* < 0.01; ***: *p* < 0.001.

Using STRING, a protein‐protein interaction (PPI) network was generated based on the 220 DEGs in the UD HAp group, which identified 10 hub genes including COL1A1 and COL4A6. Combining the enrichment analysis of the overall DEGs and the 10 hub genes, it is strongly suggested that the organization of the ECM plays a major role in regulating cell behavior on the UD HAp NRs. This finding supports the observation of highly aligned ECM using multiphoton microscopy and its critical role in the formation of aligned calcium deposits. Three enriched Kyoto Encyclopedia of Genes and Genomes (KEGG) pathways, namely ECM‐receptor interaction, relaxin signaling pathway, and focal adhesion, were identified based on the 10 hub genes (**Figure** **5**A; Figure S31, Supporting Information). Interestingly, it was noted that COL1A1 (encoding α1 chain of COL1) and COL4A6 (coding α6 chain of COL4) were involved in all 3 enriched pathways. Further search in the KEGG database confirmed that COL1A1 and COL4A6 are also part of the PI3K‐Akt signaling pathway (Figure S32, Supporting Information), which is a subclass pathway within the focal adhesion pathway. Based on these findings, a hypothesis was proposed that UD HAp NRs regulate the expression of COL1A1 and COL4A6 in hBMSCs, leading to the formation of aligned ECM. The alignment of ECM then facilitates hBMSCs elongation, increases tension on the cytoskeleton, activates the PI3K‐Akt signaling pathway, upregulates osteogenesis‐related genes, enhances osteogenesis, and promotes the formation of ordered calcium deposits.

**Figure 6:**
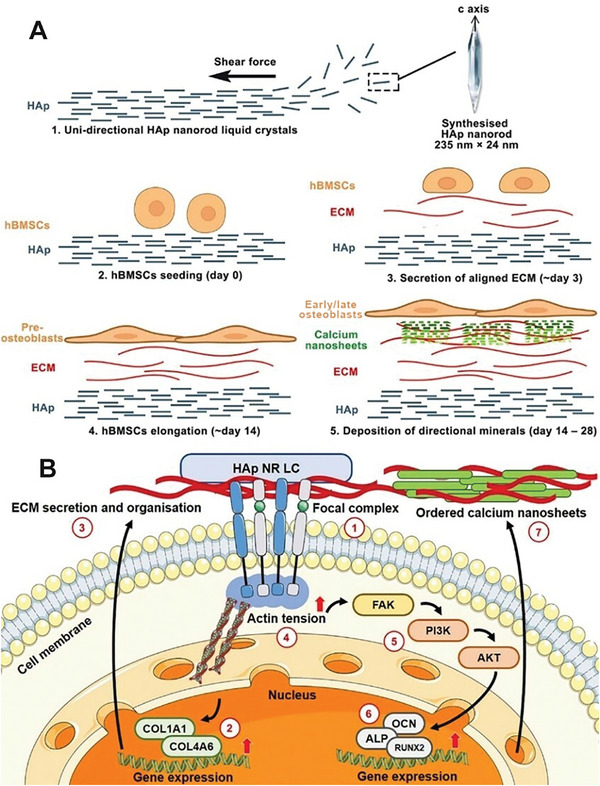
Synopsis of LC structure‐guided ordered mineralization and the associated molecular mechanisms. A) Overview of this study. 1) Biomimetic hydroxyapatite nanorod liquid crystals (HAp NR LC) are synthesized and aligned at a large scale, on which 2) the hBMSCs are seeded and underwent osteogenic differentiation. 3) Aligned ECM is first formed at early time points, followed by 4) elongation, differentiation, and maturation of committed preosteoblasts from day 14 of differentiation onward. 5) Then ordered calcium nanosheets are deposited and accumulated by early/late osteoblasts between days 14 and 28. B) Schematic of the signaling pathway of LC‐induced ordered calcium deposition based on the RNA sequencing, bioinformatics analyses, and validation experiments in this study. It is a cascade comprised of several steps: ① hBMSCs attach to, sense, and recognize unidirectional HAp (UD HAp) via focal complexes; ② The topographic information is transduced into the nucleus and activate the expression of hub genes COL1A1 and COL4A6; ③ Using the UD HAp as the primary template, aligned ECM is formed and results in the elongation of hBMSCs; ④ The tension of F‐actin increases due to elongation, which ⑤ activates the PI3k‐Akt signaling pathway, then ⑥ upregulates the gene expression of osteogenic genes ALP, OCN, and RUNX2. ⑦ Ordered calcium nanosheets are deposited using the aligned ECM as the secondary template.

To verify these findings, RNA interference (RNAi) was performed to interfere with the expression of COL1A1 and/or COL4A6 in hBMSCs cultured on UD HAp NRs. In the absence of either COL1A1 or COL4A6, hBMSCs lost their elongated morphology, detached from the substrate, and exhibited significant apoptosis (Figure 5D). The total DNA assay (Figure 5B) indicated reduced cell viability in the siCOL1A1 and/or siCOL4A6 groups. Interestingly, ALP activity (Figure 5C) increased in the absence of COL1A1 and/or COL4A6, which may indicate an osteoarthritis‐like development rather than improved early‐stage osteogenesis.^[^ ^39^ ^]^ This finding was supported by the significantly downregulated expression of osteogenic genes in the siCOL1A1 and/or siCOL4A6 groups (Figure S33, Supporting Information). Western blotting (Figure 5E) confirmed the activation of the PI3K‐Akt signaling pathway during hBMSC‐LC interaction and the involvement of the hub genes, COL1A1 and COL4A6. A full interpretation of western blot results is presented in Note S4 (Supporting Information). Additionally, the influence of RNAi on FAs was investigated (Figure S34, Supporting Information). Control hBMSCs displayed randomly distributed FAs, while elongated hBMSCs formed clusters of FAs at the ends of the cell body. However, after silencing COL1A1 and/or COL4A6, FAs contracted and localized around the nucleus. A synopsis of this study and an elaboration of the molecular mechanisms involved are shown in **Figure** **6** and Discussion.

### 2.6 Further Discussion

Despite decades of intensive research, hierarchically ordered bone regeneration in vitro and bone repair *in vivo* remains elusive, and the underlying mechanism is still not fully understood. To the best of our knowledge, none of the current attempts focusing on anisotropic structures have been able to achieve controlled calcium deposits at multiple scales. It is becoming increasingly evident that cells are noticeably influenced by their surrounding microenvironment.^[^ ^40^ ^]^ Thus, all current methods are primarily focused on regulating the behavior of cells by mimicking their native microenvironment. The exceptional mechanical properties are the defining characteristics of bone. Ultimately, the aligned mineralized collagen fibers that form anisotropic LC structures contribute the majority of the strength of bone.^[^ ^41^ ^]^ When designing our experiments, we did not introduce collagen to create a composite substrate, as it can complicate the microenvironment, and interfere with ECM visualization, and the structure and stiffness of the current collagen‐based composite do not match those of native bone. Therefore, we selected HAp as a bone‐like substrate. HAp is the primary inorganic component of bone, can be manipulated into a liquid crystalline state, and possesses sufficient stiffness to promote osteogenesis. The HAp NR LC substrate provides a simplified but unique model that isolates the most critical feature, i.e., the LC characteristics, from other intertwined factors in the microenvironment. Several foundational studies by Kato et al. have described the self‐assembly behavior of HAp NR LC^[^ ^42^ ^]^ and its potential biological applications.^[^ ^25^ ^]^ By contrast, we have focused on the novel application of HAp NR LC in guiding osteogenesis.

Our results demonstrate that Cit/Tb‐HAp maintains a nematic LC phase after Tb doping. By leveraging self‐assembled anisotropic LC flow, a macroscope unidirectional alignment of Cit/Tb‐HAp LC was achieved with the assistance of the drag force generated by spin‐coating. The HAp substrate acted as a calcium reservoir in the early stages and as a source of calcium later on. After confirming that the substrates did not significantly influence the proliferation of hBMSCs, the subsequent osteogenic differentiation test revealed that the UD HAp NRs induced the most significant osteogenesis at both the gene and protein levels. The morphology and FA distribution of hBMSCs provide some insights into the underlying mechanism. The distribution of FAs closely resembles the distribution of traction stress. The elongated morphology and two‐end distribution of FAs in the UD HAp group resulted in optimal cytoskeletal tension, leading to higher differential gene expression.^[^ ^43^ ^]^ In general, integrin‐mediated cell‐matrix adhesions connect the cytoskeleton to the extracellular matrix and allow cells to sense the substrate's features. Cells anchor to a surface through FAs and transmit the tension on the cytoskeleton by regulating FAs. When the expression of COL1A1 and/or COL4A6 was inhibited, ECM organization and FA formation were disrupted and the cells exhibited a less stressed morphology. This evidence suggests that ECM organization plays a crucial role as a mechanical transducer. The aligned ECM on the UD HAp NRs provides an ideal mechanotransduction substrate, resulting in enhanced osteogenesis with a hierarchically ordered structure.

In this study, we provide insight into the anisotropic growth of calcium deposits on the UD HAp NRs. Through various characterization techniques including SAXS, FE‐SEM, STEM, HR‐TEM, and AFM, we investigated the formation and growth of calcium deposits at multiple scales during osteogenesis. The calcium deposit exhibited guided and even distribution between the elongated differentiated cells and the highly aligned ECM generated on the UD HAp NRs. Calcium deposit comprised multi‐layer apatite crystal nanosheets with a lamellar distance of 36.4 nm. Within each layer, the slender crystal nanosheets tightly aggregated into an ordered secondary structure with a sheet‐sheet distance of 36.8 nm, showing a preferred orientation toward (002) plane. More structural details of the calcium nanosheets and mineral phase of calcium deposits will be further reviewed and confirmed at high resolution using advanced 3D TEM tomography and EDX at higher resolutions. RNA‐Seq and bioinformatics analysis revealed that the ECM‐related BP GO terms are highly enriched in overall differentially expressed genes (DEGs) and the 10 hub genes in the UD HAp group. This suggests that ECM organization may play a pivotal role in the interaction between cells and the LC phase, as well as in the formation of ordered calcium deposits. Further enrichment analysis of the hub genes identified 2 genes, COL1A1 and COL4A6, as well as the PI3k‐Akt signaling pathway, as potential key players in the aligned ECM and formation of ordered calcium deposits. These findings were subsequently verified by Western blotting and PCR techniques. Based on current knowledge, COL1 functions in the early event of osteogenic differentiation, while COL4 is a major component of basement membranes. We propose that the COL1A1 is directly linked to the formation of ECM, which is mainly composed of type I collagen, while COL4A6 mediates cell attachment to substrates, which is a fundamental activity prior to cell survival and mechanical signal transduction.

In the physiological process of bone development, longitudinal growth occurs at the metaphyseal areas of long bones, where cartilaginous tissue is first generated, followed by mineralization to form primary new bone (disordered hard tissues). Subsequently, biomechanical stimulus remodels the bone into more ordered structures, which relies on the resorption of disordered bone and the formation of ordered bone by osteoclasts or osteoblasts, respectively, in response to physiological forces. In bone regeneration after injury, there is a similar process but a few more stages (hematoma, inflammation, and resolution) will take place before fibrous and cartilaginous tissues can grow. In the end, the regenerated bone is reconstructed after recanalization of the medullary cavity and restoration of blood circulation. Osteoblasts play an important role in osteogenesis. They synthesize new collagenous organic matrix, then regulate mineralization of the matrix by concentrating calcium and phosphate, and suppress mineralization inhibitors such as pyrophosphate or proteoglycans. After being surrounded by and buried within the mineralized matrix, the osteoblasts become osteocytes. In native bone, the ECM exhibits a highly oriented and hierarchical structure across various length scales, ranging from the nano‐ to the macro‐scale. Cells are capable of sensing topological features as small as 5 nm on a substrate.^[^ ^44^ ^]^ Usually, the micro‐ and nanoscale ECM topography influences cells by organizing cell morphology, which, in turn, regulates the tension of the actin cytoskeleton and activates the intracellular signaling cascade mediated by integrins.^[^ ^45^ ^]^ These molecular events ultimately determine the fate commitment of stem cells.^[^ ^46^ ^]^ Despite decades of intensive research, bone regeneration in vitro still cannot be achieved, and the mechanism remains elusive. Factors linking ordered mineralization and stem cells/osteoblast lineage also remain elusive. In the context of bone formation, the aligned ECM has a significant impact on the orderly deposition of bone tissue. Evidence suggests that the ECM proteins can be detected as early as 3 days after cell seeding and gradually accumulate over time,^[^ ^47^ ^]^ while cell elongation and mineralization become evident ≈14 days of differentiation, as observed in our study. Based on those observations, we hypothesized that the aligned ECM serves as a template for subsequent calcium deposition, mirroring the in situ formation process of native bone. Specifically, the UD HAp NRs serve as the primary template that is sensed by hBMSCs, and then triggers the expression of COL1A1 and COL4A6. The genes, in turn, promote the formation of aligned ECM on the UD HAp NRs, serving as the secondary template for the subsequent in situ mineralization process. Concurrently, the aligned ECM induces a more elongated cell morphology. The increased tension exerted on the actin cytoskeleton activates the PI3k‐Akt signaling pathway, which further upregulates the expression of the osteogenic gene. Ultimately, a coordinated sequence of events culminates in the orderly deposition of calcium, leading to the formation of an ordered calcium deposit.

## 3 Conclusion

In summary, we developed effective methods to control the growth of calcium deposits and established a link between long‐range ordered mineralization and the PI3k‐Akt signaling pathway. Key regulatory genes, such as COL1A1 and COL4A6 have been identified and the central role of the aligned ECM in regulating ordered bone formation has been highlighted. These findings hold great promise for the future design and fabrication of large‐scale biomimetic artificial bone constructs in laboratory settings. Furthermore, the identified genes and pathways can potentially serve as targets for drug discovery and gene therapy for treating bone disorders. Conversely, by downregulating these genes and pathways, it may be possible to prevent cardiovascular calcification, which occurs when vascular cells undergo a phenotype switch to osteoblast‐like cells. It is also important to note that these findings may have broader applications beyond the scope of this specific study. The principle elucidated here regarding the interaction between cells and LC‐ordered ECM, as well as their regulatory mechanisms, may have implications for other types of biological LCs and similar applications. This progress significantly contributes to our fundamental understanding of how cells and tissues interact with LC‐ordered ECM, paving the way for further discoveries and applications in tissue engineering and regenerative medicine.

## 4 Experimental Section

### Synthesis of HAp with Different Doping

HAp nanorods with or without trisodium citrate (Cit) and/or Tb doping were synthesized utilizing a hydrothermal method described by Jin et al.^[^ ^27^ ^]^ and Yang et al.^[^ ^28^ ^]^ with minor revisions. Briefly, the synthesis followed an ultimate molar ratio of Ca/Tb = 96:4, (Ca + Tb)/Cit = 3:4 or Ca/Cit = 3:4 without Tb doping, and (Ca + Tb)/P = 1.67 or Ca/P = 1.67 without Tb doping. In a typical experiment, to obtain Cit/Tb‐HAp nanorods, 10 mL 0.006 mol C_6_H_5_Na_3_O_7_·2H_2_O solution was added to 10 mL 0.00432 mol Ca(NO_3_)_2_ solution with continuous stirring. Then, 10 mL 0.0027 mol (NH_4_)_2_HPO_4_ was added dropwise into the aforementioned mixture with vigorous stirring, followed by adding 5 mL 0.00018 mol Tb(NO_3_)_3_ into the mixture. After that, the pH was brought to 7.0 utilizing 1 M NaOH to obtain the final solution. Stirring was maintained at room temperature for another 30 min to allow complete mixing. The final solution was then transferred into a 100 mL Teflon‐lined autoclave reactor, placed into an oven, and subjected to hydrothermal treatment at 180 °C for 24 h to form Cit/Tb‐HAp NRs. After the hydrothermal treatment, the autoclave reactor was allowed to cool naturally to room temperature before being opened. The obtained products were washed through a three‐cycle centrifugation‐washing (10 000 rpm) process with deionized water and absolute ethanol. The HAp with different doping was synthesized following the same protocol but without adding the corresponding chemical(s). Finally, a portion of the products was dried in a freeze‐dryer under vacuum conditions for 24 h to obtain a lyophilized powder. Some Cit/Tb‐HAp nanorods were re‐dispersed in deionized water and then centrifuged to obtain a series concentration of LC samples for further characterization. Four groups of HAp samples were synthesized: HAp, Tb‐HAp Cit‐HAp, and Cit/Tb‐HAp. Formulas for different samples are listed in Table S6 (Supporting Information). To distinguish HAp synthesized utilizing different formulas, the trisodium citrate‐treated and terbium ions‐doped HAp was named Cit/Tb‐HAp; the trisodium citrate‐treated but not terbium ions‐doped HAp was named Cit‐HAp; the terbium ions‐doped but not trisodium citrate‐treated HAp was named Tb‐HAp; the nontreated and nondoped HAp was named HAp, while the commercially obtained HAp was named commercial HAp.

### Fourier Transform Infrared (FTIR) Characterization

All HAp samples were freeze‐dried for 48 h before FTIR scan. The functional groups of lyophilized HAp samples were identified by a Jasco FT/IR‐4100 Spectrometer (Jasco UK Ltd, Essex, UK) using attenuated total internal reflection (ATR). The IR spectrum was scanned at room temperature from 4000–400 cm^−1^ with a resolution of 2 cm^−1^.

### X‐Ray Diffraction (XRD) Analysis

The crystalline structure of the HAp samples was examined using XRD. Lyophilized colloids were sealed in quartz capillaries (Capillary Tube Supplies Ltd, Cornwall, UK) with a diameter of 0.7 mm. For coverslip samples, they were securely attached to the sample holder and positioned perpendicular to the incident beam. Scans were performed on an STOE STADI P Powder Diffractometer System (STOE & Cie GmbH, Darmstadt, Germany) equipped with Ni‐filtered Cu Kα1 radiation (*λ* = 1.54060 Å, U = 40 kV, I = 40 mA). The samples were scanned from 2θ = 2° to 65° at a scan rate of 6°/min. The obtained XRD spectra and patterns of the samples were analyzed and compared with the standard hydroxyapatite XRD data (Joint Committee on Powder Diffraction Standards, JCPDS No. 09–0432) from the International Centre for Diffraction Data (ICDD) database. The crystallinity index (*CI*) was used to evaluate the crystallinity of HAp, and the texture index *R* was used to infer the preferred orientation of HAp NRs. See Supporting Information for formulas.

### Macroscopic Fluorescence Imaging and Fluorescence Spectrum Scanning

Prior to fluorescence spectrum scanning, the HAp powders were illuminated by room light or a 365 nm UV light torch. The photos were captured using a Canon EOS M100 camera (Canon, Tokyo, Japan). The fluorescence emission spectra of the HAp samples were generated using a Perkin Elmer LS50‐B Fluorescence Spectrometer (Perkin Elmer, Beaconsfield, UK). The HAp powders were sealed in a 2 mm thick quartz cuvette and loaded onto a customized sample holder. The front side of the cuvette was set at 45° to the incident excitation beam. Excitation/emission mapping was first performed with an excitation wavelength = from 220 nm to 500 nm using a 10 nm resolution. The most efficient excitation wavelength was then located and applied to all HAp samples for emission spectrum acquisition.

### Liquid Crystal Phase Formation

Cit/Tb‐HAp NRs were utilized to study their LC phase. To determine the critical concentration of LC phase transition, 200 µL of Cit/Tb‐HAp NRs stable colloid (≈15 wt.%, in DI water) was dispersed by ultrasonication and filled into a series of 2 mm thick quartz cuvettes without lids. The cuvettes were then left open and placed in an oven at 40 °C to accelerate evaporation. At each regular interval, the cuvettes were cooled down to room temperature, then positioned between 2 homemade orthogonal polarized filters and illuminated with a white light source from one end. A camera was placed at the opposite end of the light source to capture photos of the LC phase transition. Once the phase transition became apparent, the cuvette was then transferred to another oven at 80 °C for 48 h to ensure thorough drying and facilitate the determination of the LC concentration. To observe the growth of Cit/Tb‐HAp LC domains, 100 µL of Cit/Tb‐HAp colloid at ≈17 wt.% was properly sealed in a 2 mm thick quartz cuvette and left stand still for 7 days. The cross‐polarized photos were captured on days 0, 1, 2, and 7. See Supporting Information for formulas of LC concentration and methods for LC phase observation at a higher resolution.

### Coating Substrate Preparation

Preferably, commercial HAp, synthesized HAp without doping (hereafter referred to as “HAp”), and Cit/Tb‐HAp were selected for substrate coatings on polydimethylsilaxane (PDMS). Five groups were set up: control group (PDMS), commercial HAp (PDMS + commercial HAp), HAp (PDMS + synthesized HAp without any doping), MD HAp (PDMS + Cit/Tb‐HAp), and UD HAp (PDMS + Cit/Tb‐HAp). Specifically, 15 µL of PDMS resin (Sylgard 184, Dow Corning, Michigan, USA) was spin‐coated (5000 rpm, steady time 15 s, acceleration rate 300 rpm ^−1^s) onto 9 mm coverslips utilizing the Navson NT12000 V1 Spin Coater (Navson Technologies Pvt Ltd., Tamil Nadu, India). For the control group, the obtained PDMS‐coated coverslips were put into an oven and incubated at 80 °C for 30 min to allow a thorough cure. For all the other groups, the PDMS‐coated coverslips were incubated at 80 °C for 5 min until reaching a sticky semi‐cured status. The 35 wt.% colloids of commercial HAp, HAp, and MD HAp groups were then prepared by dip‐coating a thin layer of corresponding colloids onto the top of the semi‐cured PDMS. In terms of the UD HAp group, 20 µL of Cit/Tb‐HAp suspension was spin‐coated (8000 rpm, steady time 15 s, acceleration rate 300 rpm ^−1^s) onto the top of semi‐cured PDMS. After a thorough PDMS cure, all the coverslips were soaked in DI water on a shaker for 24 h to wash off unattached nanoparticles thoroughly.

### Cell Proliferation on Different HAp‐Coated Surfaces

Disinfection for the aforementioned 5 groups of substrates was first carried out utilizing 3% hydrogen peroxide (Sigma‒Aldrich, Missouri, USA) soaking for 2 h, followed by repeated sterile phosphate‐buffered saline (PBS) rinsing to remove any residual hydrogen peroxide and conditioning in mesenchymal stem cell medium (MSCM) at 37 °C for 2 h before cell seeding. Three replicates were set in each group. An equal number of hBMSCs (2.5 × 10^3^) were seeded onto the surface of coverslips in 48‐well plates. Then, fresh MSCM was added up to 200 µL in each well. All cells were cultured at 37 °C and 5% CO_2,_ and fresh medium was replenished every 3 days for a total period of seven days. The PrestoBlue HS assay (Thermo Fisher Scientific, Massachusetts, USA), ToxiLight cytotoxicity assay (Lonza, Basel, Switzerland), and total DNA assay (Sigma‒Aldrich, Missouri, USA) were performed on days 1, 3, and 7 to comprehensively evaluate the proliferation of hBMSCs. For RNAi experiments, the total DNA assay was carried out on day 14 of osteogenic differentiation after RNAi on days 0, 7, and 12. The assays were carried out following the manufacturer's instructions. Briefly, first, the ToxiLight cytotoxicity assay was conducted by removing 20 µL of cell supernatant and transferring it to a luminescence 96‐well white plate with a clear bottom. Then, 100 µL AK detection reagent was added to each well, and luminescence intensity was detected on a Plate Reader Infinite M200 PRO (Tecan, Männedorf, Switzerland). In terms of the PrestoBlue HS assay, all old culture medium was disposed of and replenished with 180 µL fresh MSCM and 20 µL PrestoBlue HS reagent. Then, the cells were incubated at 37 °C and 5% CO_2_ for 2 h prior to measuring the fluorescence intensity at an excitation/emission wavelength of 560/590 nm on an Infinite M200 PRO plate reader. After that, the coverslips were washed with PBS 3 times, and 100 µL fresh PBS was added to each well for the purpose of performing the total DNA assay. Cells in PBS underwent a three‐cycle freeze‐thaw process to disrupt the cell membrane and release the DNA into the lysate. A standard curve with a range of 10–500 ng mL^−1^ DNA was established, followed by pipetting 10 µL lysate of each sample into 2 mL bisBenzimide H 33 258 Solution (0.1 mg mL^−1^). Then, 100 µL of each mixed solution was pipetted into a black 96‐well plate, and the fluorescence intensity was measured at an excitation/emission wavelength of 360/460 nm.

### Osteogenic Differentiation

Osteogenic differentiation culture was similar to proliferation culture, but the seeding density of hBMSCs was 2.5 × 10^4^ per well on coverslips in 48‐well plates. The seeded hBMSCs were cultured in MSCM at 37 °C and 5% CO_2_ for 24 h to ensure cell attachment. After that, the medium was replaced with mesenchymal stem cell osteogenic differentiation medium (MODM) to induce osteogenic differentiation in the following days. The day MSCM was replaced with MODM was counted as day 0 of osteogenic differentiation. Detailed grouping and treatment information are illustrated in Figure S36 (Supporting Information).

### Real‐Time PCR (RT‒PCR) Analysis

The osteogenic differentiation of hBMSCs was evaluated by examining the gene expression of osteogenic‐related genes, including osteocalcin (OCN), alkaline phosphatase (ALP), and runt‐related transcription factor 2 (RUNX2), and the housekeeper gene GAPDH on days 7, 14, 21, and 28. For RNAi experiments, PCR was carried out on day 21 of osteogenic differentiation after RNAi on days 0, 7, 14, and 19. The customized primers were manufactured by Invitrogen (Massachusetts, USA), and the full list of primer sequences is shown in Table S7 (Supporting Information). Real‐time PCR was carried out utilizing the Cells‐to‐CT 1‐Step Power SYBR Green Kit (Invitrogen) according to the manufacturer's instructions. In a typical experiment, hBMSCs were first rinsed with ice‐cold PBS, followed by the addition of lysis solution and DNase and incubation for 5 min at room temperature. Then, the stop solution was added to the wells to terminate cell lysis. Two microliters of lysate were then mixed with 18 µL of RT‒PCR Master Mix to obtain a 20 µL reaction system. Next, one‐step PCR was carried out on the StepOnePlus Real‐Time System (Thermo Fisher Scientific, Massachusetts, USA) with the program settings in Table S8 (Supporting Information). The housekeeping gene GAPDH was used as an internal control to normalize the reactions. The relative gene expression was evaluated by the 2^–ΔΔCt^ method.

### Verification of Aligned Calcium Deposits via Small Angle X‐Ray Scattering (SAXS)

The extreme small‐angle (ESAXS) and wide‐angle X‐ray scattering (WAXS) patterns of LC Cit/Tb‐HAp (≈28 wt.%) and the SAXS patterns of MD and UD HAp samples on days 0 and 28 of osteogenic differentiation were examined by a WAXS/SAXS instrument (Ganesha 300XL, SAXSLAB, Denmark). The capillary samples were appropriately sealed in 1.5 mm borosilicate capillaries (Capillary Tube Supplies Ltd., Cornwall, UK) and the coverslip samples were fixed using 4% PFA and then dehydrated through a graded series of ethanols prior to the scanning. Both capillary and coverslip samples were firmly attached to the sample holder and placed perpendicularly to the incident beam. An empty capillary or coverslip was used as a background reference for deduction where applicable. All X‐ray scattering measurements were carried out using a 2 mm beam stop in vacuum at room temperature with a wavelength of 1.5418 Å for the Cu anode. Silver behenate (peak at 0.1076 Å^−1^) was used for calibration prior to sample scanning. The scanning range was from 0.0035 to 0.18 Å^−1^ (ESAXS), or from 0.007 to 0.25 Å^−1^ (SAXS), or from 0.07 to 2.8 Å^−1^ (WAXS), and the beam size at the sample position was ≈ 0.4 × 0.4 mm^2^. See Supporting Information for detailed scanning parameters and formulas.

### Cell Morphology Imaging Using DDC‐SEM and FE‐SEM

Density‐Dependent Color Scanning Electron Micrograph (DDC‐SEM) imaging was carried out to observe the morphology and calcium deposits from hBMSCs on different substrates. All coverslip samples on days 0, 7, 14, 21, and 28 after osteogenic differentiation were fixed with 4% PFA and dehydrated utilizing a graded series of ethanols. Next, the uncoated samples were observed on a Zeiss Sigma 500 VP FE‐SEM operated at 12 kV in the Variable Pressure (VP) mode of 50 Pa. In high vacuum mode, the angle selective backscatter (AsB) detector was used to highlight cell morphology, and the VPSE G4 detector was used to acquire topographic information. ImageJ software was then used for image post‐processing. The false‐color images were generated by allocating AsB images into the Red channel and VPSE G4 images into the Green channel.

### Surface Information of Coatings and Calcium Deposits Characterized by Atomic Force Microscopy (AFM)

The surface information of samples on days 0 and 28 of osteogenic differentiation was acquired using the amplitude modulation‐frequency modulation (AM‐FM) method on an AFM (MFP‐3D Stand Alone AFM, Asylum Research, CA, USA). The AC160TS‐R2 cantilevers (Asylum Research, CA, USA) comprising silicon tips with a radius of 7 nm and a spring constant of 26 N/m were used. The deflection sensitivity and the spring constant of the cantilever were determined using the GetReal Automated Probe Calibration feature, followed by automatically tuning the primary resonance frequency (≈ 250 kHz) and the secondary resonance frequency (≈ 1.4 MHz). The scanning size was set to 1 × 1 µm with 256 pixels at 1.0 Hz. The primary resonance frequency was used to acquire topography (height and phase), while the secondary resonance frequency depicted images of the frequency, dissipation, and loss tangent of the samples. See Supporting Information for more details.

### Statistical Analysis

Data presentation and statistical analyses were performed in the SPSS Statistics (Version 22.0, IBM, Armonk, US). All descriptive data are presented as mean and standard deviation (SD). For statistics of biological experiments, each condition *n* = 3. For statistics of particle orientation, each condition *n* = 300. The one‐way analysis of variance (ANOVA) was used for assessing differences among 3 or more groups. Prior to conducting the one‐way ANOVA, the homogeneity of variances assumption was tested using Levene's test. LSD‐t test was applied if the variance was not the same, otherwise, the Tamhane's T2 test was used for further comparison. *p *< 0.05 was considered a statistically significant difference with a 95% confidence interval.

## Conflict of Interest

The authors declare no conflict of interest.

## Supporting information

Supporting Information

Supporting Information

## Data Availability

The data that support the findings of this study are available from the corresponding author upon reasonable request.
