# Supplementary material for: Liquid Crystalline Hydroxyapatite Nanorods Orchestrate Hierarchical Bone‐Like Mineralization
Source: Small. 2024 Aug 23;20(52):2310024. doi: 10.1002/smll.202310024 (PMC11673523; doi:10.1002/smll.202310024)
Supplement: Supplementary file 1 — Supporting Information [file SMLL-20-2310024-s001.docx]

Liquid Crystalline Hydroxyapatite Nanorods Orchestrate Hierarchical Bone-Like Mineralization

Jishizhan Chen^1^, Martin Birchall^2,3^, Alexander J. MacRobert^1^, Wenhui Song^1,*^

J. Chen, A. J. MacRobert, W. Song

UCL Centre for Biomaterials in Surgical Reconstruction and Regeneration, Department of Surgical Biotechnology, Division of Surgery & Interventional Science, University College London; Rowland Hill Street, London, NW3 2PF, United Kingdom.
E-mail: [w.song@ucl.ac.uk](mailto:w.song@ucl.ac.uk)

M. Birchall

UCL Ear Institute, University College London, 332 Grays Inn Road, London WC1X 8EE, United Kingdom.

Royal National Ear Nose and Throat and Eastman Dental Hospitals, University College London Hospitals; 47-49 Huntley Street, London, WC1E 6DG, United Kingdom.

Supporting Information
Materials and Methods

Materials for HAp synthesis

Calcium nitrate tetrahydrate (≥ 99.0%), diammonium hydrogen phosphate (≥ 99.0%), trisodium citrate dihydrate (abbreviated as Cit in the following context, ≥ 99.0%), terbium(III) nitrate hexahydrate (abbreviated as Tb in the following context, ≥ 99.9%), sodium hydroxide (1 M), and absolute ethanol (≥ 99.0%) were purchased from Fisher Scientific UK Ltd. (Leicestershire, UK). All chemicals were used as received without further purification. Deionized (DI) water was used throughout. Calcium nitrate tetrahydrate, Ca(NO_3_)_2_, and diammonium hydrogen phosphate, (NH_4_)_2_HPO_4_, served as sources of calcium and phosphate, respectively. Trisodium citrate, Na_3_C_6_H_5_O_7_, acted as a dispersant facilitating crystal growth along the c-axis and providing sufficient electrostatic repulsion for colloidal stability.^[1]^ To track the HAp contribution to osteogenesis of hBMSCs, terbium ions (Tb^3+^) were doped into HAp. Tb^3+^ ions can substitute for the ion sites of Ca^2+^ ions in the lattice,^[2]^ enabling identification of the calcium source of mineral secretion in cells and endows HAp with fluorescence. Nucleation and growth of crystals were maintained at 180℃ in an autoclave for 24 hrs.

X-ray diffraction (XRD) analysis

Formulas for calculating crystallinity index (*CI*): Here we utilized two popular methods (by peak intensity or area) to calculate the *CI*. The first method is described by Person et al.,^[3]^ given by measuring the height of peaks (202), (300), (211), and (112) using the equation

${(CI)}_{XRD}= \frac{I_{(112)}+I_{(300)}+ I_{(202)}}{I_{(211)}}$ (1)

where

${(CI)}_{XRD}$ = crystallinity of HAp calculated from XRD data.

$I_{(112)}$, $I_{(300)}$, and $I_{(202)}$ = relative height (percentage relative to $I_{(211)}$), measured from the top of peak (112), (300), or (202) to the ‘valley’ dividing them from the next peak.

$I_{(211)}$ = 100%. Height measured from the top of peak (211) to the baseline between *2θ* = 24°– 38° used as a reference to the other peaks.

For comparison, the second *(CI)_XRD_* calculation method was given by dividing the integrated area of all the crystalline peaks by the integrated area of all the crystalline and amorphous peaks,^[4]^ using the equation:

${(CI)}_{XRD}= \frac{Area of all the crystalline peaks}{Area of all the crystalline and amorphous peaks}$ (2)

The preferred orientation of HAp grains can be inferred using the texture index *R* proposed by Low *et al.*:^[5]^

*R_hkl_* = [*I*_(211)_/*I*_(_*_hkl_*_)_]/*K_hkl_* (3)

where

$I_{(211)}$ and $I_{(hkl)}$ = the measured intensity of (211) and the interested (*hkl*).

$K_{(hkl)}$ = the ratio of the standard intensity of the (211) to the interested (*hkl*) calculated from ICDD data.

When *R* = 1.0, the HAp grains are randomly distributed and have no preferred orientation (round shape), whereas *R* ＞ or ＜ 1.0 indicates that HAp grains have a preference to grow along the corresponding plane. The further the *R* value is from 1.0, the stronger the preference. In this study, we employed *K*_(200)_ = 10.0, *K*_(300)_ = 1.67, and *K*_(002)_ = 2.5 from a well-crystallized HAp PDF card No. 09-0432.

Based on Low’s equation, we propose a ‘distance’ to *R* = 1.0 (*DtR_hkl_*) of a (*hkl*), expressed as a percentage of how close the *R_hkl_* value is to 1.0. This provides a more intuitive judgement of the preferred orientation. The higher the percentage, the stronger the preference. The equation below is easy to understand:

*DtR_hkl_* = |1- *R_hkl_*| × 100% (4)

where *R_hkl_* can be calculated from Equation (3).

The average grain size (*D*) is calculated from three samples in the same group using the Scherrer equation:

$D= \frac{K\lambda}{\beta cos \theta}$ (5)

where

$D$ = the mean dimension (nm) of the homogeneous crystallites along an axis perpendicular to the *hkl* system considered.

$K$ = the Scherrer constant 0.89.

$\lambda$ = X-ray wavelength, 1.54060 Å for Cu source.

$\beta$ = full width at half maximum (FWHM) of the diffraction peak.

$\theta$= Diffraction angle.

Liquid crystal phase formation

The LC concentration at phase transition is calculated from the weight difference of cuvettes using the following formula:

$C= \frac{W_{dry}-W_{empty}}{W_{LC}-W_{empty}} \times100\%$ (6)

where

$C$ = LC concentration (wt%).

$W_{dry}$= weight (mg) of the dried HAp powder and cuvette.

$W_{LC}$ = weight (mg) of the HAp LC and cuvette.

$W_{empty}$ = weight (mg) of the empty cuvette.

To identify the type of LC phase of Cit/Tb-HAp at a higher resolution, a thick sample was prepared by filling 100 μL of approximately 35 wt% Cit/Tb-HAp LC into a 2 mm thick quartz cuvette and sealed appropriately. The sample was placed between cross-polarized light under a microscope (Olympus BX63) and imaged when the sample was rotated from 0° to 45° or 90° using a 10x lens.

HAp particle morphology, size, and elemental chemistry information

The HAp powder suspension (in DI water) was placed on top of copper grids with Lacey carbon films (Agar Scientific, Stansted, UK) and excess liquid was removed using triangular filter paper from the back side of the grid. The particle morphology, size distribution, and elemental chemistry information of HAp were examined using a Zeiss Sigma 500 VP field emission scanning electron microscope (FE-SEM, Zeiss, Germany) equipped with a STEM detector and an embedded SmartEDX, operating at 20 kV under vacuum conditions. In each group, the length and width of 300 particles were measured using ImageJ software (version 1.52a, National Institute of Health, US).

Evaluation of the alignment efficiency of spin coating

The MD and UD HAp NR samples without hBMSCs were mounted onto aluminum stubs with conductive carbon ribbons and coated with gold (sputtering time 60 s) using a Quorum Q150R Plus Gold Coater (Quorum, East Sussex, UK). The sample morphology images were taken using a Zeiss Sigma 500 VP FE-SEM with a Secondary Electrons (SE2) detector operated at 1 kV in vacuum. The orientation of 300 particles in each group was measured using ImageJ software. For MD HAp NRs, the horizontal direction was considered to be 0° in each view, while the direction of the shearing force was set to 0° for the UD HAp NRs. The angle (±90°) between the particle and the 0° direction was measured and calculated as the mean ± deviation.

Optical and section views of coated substrates

The gross polarizing view was obtained by placing substrates between the aforementioned homemade polarizing gadget and a 1/4 λ compensator (Leica Microsystems, CAT #11513570, Wetzlar, Germany). The compensator was set at 45° to the analyzer. Photos were taken with a Canon EOS M100 camera. For microscopic polarized views of the substrates, a Leica DM6 FS Microscope (Leica, Wetzlar, Germany) equipped with polarizing optics was used, and images were captured using the Leica LAS X software platform. To study the layer separation of the substrates, substrates were quick-frozen in liquid nitrogen for 1 min to become brittle and then broken up into pieces using forceps. The section sides were mounted upward on aluminum stubs, sputtered with gold (60 s), and imaged via a Zeiss Sigma 500 VP FE-SEM with an SE2 detector operated at 1 kV in vacuum.

Cell pre-processing

Human bone marrow-derived mesenchymal stem cells (hBMSCs, CAT #7500, ≥ 5 x 10^5^ cells/vial, P0), mesenchymal stem cell medium (MSCM, CAT #7501), mesenchymal stem cell osteogenic differentiation medium (MODM, CAT #7531), and corresponding supplements were purchased from ScienCell Research Laboratories (Carlsbad, CA, US). The hBMSCs were shipped on dry ice as received and then stored in liquid nitrogen until use. The detailed host information for hBMSCs can be found in **Table** S7. The primary hBMSCs in the cryopreservation vial were thawed, plated in a T75 flask (Corning, NY, USA), and cultured at 37°C, 5% CO_2_ in the MSCM with 5% (fetal bovine serum) FBS, 1% mesenchymal stem cell growth supplement, and 1% penicillin/streptomycin. The medium was replaced every 3 days until the cells reached 80% confluence. After that, the cells were trypsinized and split at a ratio of 1:2 into new T75 flasks and cultured until they reached 80% confluence again. The hBMSCs at passage 4 were then harvested and utilized for further experiments.

ALP activity assay

ALP is a protein that indicates the early stage of osteogenesis. The Alkaline Phosphatase Fluorometric Assay Kit (Abcam, Cambridge, UK) was utilized to evaluate ALP activity of each group after 0, 7, 14, 21, and 28 days of osteogenic induction. For RNAi experiments, the measurement was carried out on day 14 of osteogenic differentiation after RNAi on days 0, 7, and 12. In a typical experiment, a standard curve was created according to the manufacturer’s instructions. Then, hBMSCs were trypsinized and harvested from each group. After rinsing with cold PBS, the cells were resuspended in the assay buffer and centrifuged for 3 min at 4°C and 13000 × g. Next, the supernatant was collected and added to the assay buffer with the 4-methylumbelliferyl phosphate (MUP) substrate, followed by incubation at 25°C for 30 min while being protected from light. Finally, Stop Solution was added to stabilize the reaction, and the fluorescence intensity was measured at an excitation/emission wavelength of 360/440 nm on an Infinite M200 PRO plate reader.

Calcium ion kinetics

The calcium ion kinetics were studied by monitoring the change in free calcium ion quantity in the MODM, with or without substrate and hBMSCs. A Calcium Assay Kit (Abcam, Cambridge, UK) was used to detect calcium concentrations on days 0, 3, 7, 10, 14, 17, 21, 24, and 28. The calcium concentration before and 15 min after medium change at the same day point was both detected. Briefly, a standard curve of calcium concentration was set up. Before the medium was changed, 10 μL of old medium was removed and diluted to 50 μL with deionized water, followed by the addition of 90 μL of chromogenic reagent and 60 μL of calcium assay buffer o each well. The absorbance was read at 575 nm on an Infinite M200 PRO plate reader after 10 min of incubation in the dark at room temperature. Then, the old medium was discarded and replenished with fresh medium. After 15 min, 10 μL of replenished medium was removed and subjected to the same assay as above, which reset a ‘baseline’ for calcium consumption for the following days. Finally, a corresponding 10 μL of fresh medium was added after the assay to ensure that the total volume at each starting point was unchanged. To eliminate the calcium concentration increase due to evaporation over time, the average volume per well at each time point before and after medium change was calculated. Then, the total calcium quantity per well (mmol/well) was obtained for comparison by multiplying the detected calcium concentration with the average volume, from which the calcium consumption curves were generated using the formula:

$Calcium consumption (mmol/well)=C_{0}-C_{1}$ (7)

where

$C_{0}$ = total calcium (mmol/well) 15 min after medium change at the last time point.

$C_{1}$ = total calcium (mmol/well) at the latest time point before medium change.

Raman Spectra

Raman spectra of specimens were collected using Renishaw in Via Raman microscope (Renishaw, Wotton-under-Edge, UK) equipped with a Leica DM2700 M optical microscope with a Leica N PLAN EPI 100×/0.85 objective and a CCD camera. The 100× objective and the high confocality option in the software constrains the laser spot size and ensures a precise acquisition area of ≤1 μm. Wavelength and intensity calibration were performed on a silicon standard wafer via the WiRE software (version 5.5, Renishaw) according to the manufacturer's instructions. A monochromatic 785 nm near-infrared diode laser and 1200 l/mm grating combination were used to induce the Raman scattering effect. The single point scanning range was from 900 cm^−1^ to 1100 cm^−1^ to acquire the characteristic peak of HAp at around 962 cm^−1^ and protein (phenylalanine) at around 1004 cm^−1^. The exposure time was set for 10 s, and the laser power on the specimen was 100 mW, at which sample degradation was not observed. The Raman 2D mapping was performed in an 8 μm × 8 μm area with steps of 1 μm. Then 64 points of apatite deposit signal were collected at the range of 940 cm^−1^ to 980 cm^−1^. Baseline subtraction was performed before Gaussian peak fitting at around 962 cm^−1^. The peak intensity at 962.0 cm^−1^ was then plotted into heatmap matrixes using Origin (version 2021b, Originlab, Northampton, US).

Verification of aligned calcium deposits via small angle X-ray scattering (SAXS)

In the extreme small-angle (ESAXS) and wide-angle X-ray scattering (WAXS), the distance from the sample to the detector was approximately 1491 mm (ESAXS), or 1041 mm (SAXS), or 101 mm (WAXS). The scanning time was 7200 s (ESAXS), or 1200 s (SAXS), or 600 s (WAXS). The 2D SAXS patterns were generated from the electron density contrast in the sample, and the 1D SAXS patterns were converted from the 2D patterns through integration of the scattering vector (*q*) ranging from 0.0045 Å^-1^ to 0.18 Å^-1^ (ESAXS), or from 0.01 Å^-1^ to 0.05 Å^-1^ (SAXS), or from 0.1 Å^-1^ to 2.8 Å^-1^ (WAXS), to exclude the non-signal beam stop area and cover all available signals. The 1D azimuth plots were also obtained through integration at the same *q* range from 0° to 360°. Gaussian peak fitting was applied to the asobtained 1D plots and peaks were located using the Origin 2021b software. After that, the preferred orientation of the sample was determined by the two peak positions ±90° in the azimuthal plot. The degree of alignment ($\varphi$) was calculated by the ratio of the peak areas to the overall intensity in the azimuth plot, ranging from 0 to 100%, where 0 stands for completely disordered and 100% stands for perfectly aligned:

$\varphi$ = $\frac{A_{1}}{A_{1}+ A_{0}}$ (8)

where

*A_1_* = peak area above the background baseline;

*A_0_* = background area (from *y* = 0 to the baseline).

The characteristic separation distance (*d*-spacing) is calculated by Bragg’s law:

$d= \frac{2\pi}{q}$ (9)

where

$q$ = the scattering vector.

X-ray photoelectron spectra (XPS) analysis of chemical bonds on coating surfaces

XPS can detect the chemical bonds on the very surface (normally less than 10 nm). In this study, the interaction between hBMSCs and HAp/calcium deposits was analyzed on days 0 and 28 of osteogenic differentiation. All hBMSCs on coated coverslips were first fixed using 4% PFA and dehydrated by graded series ethanol. XPS was measured on a Thermo K-Alpha+ XPS system (Thermal Fisher Scientific, Massachusetts, US) using micro-focused, monochromatized Al Kα radiation (400 μm spot size) with a pass energy of 200 eV for survey and 50 eV for high-energy resolution spectra, with energy step sizes of 1 eV and 0.1 eV, respectively. The C1 s O1 s, N1 s, Ca2p, P2p, and Tb3d were examined. All spectra were referenced to the C1 s peak (C-C, C-H) of hydrocarbons at 285.0 eV binding energy controlled by means of the well-known photoelectron peaks of metallic Cu, Ag, and Au. Data analysis was performed in CasaXPS (version 2.3.19, Casa Software Ltd, Devon, UK) using a Shirley type background and Scofield cross sections.

Cell morphology and focal adhesion (FA) imaging using confocal microscopy

To investigate the distribution of cell FAs on different surface coatings, all coverslip samples on days 0 and 28 after osteogenic differentiation were subjected to staining prior to confocal microscopy scanning. For RNAi experiments, samples on day 21 of osteogenic differentiation, after RNAi on days 0, 7, 14, and 19, were observed. All conjugated antibodies were purchased from the Signalway Antibody Company (Maryland, US) unless otherwise specified. Paxillin was stained with the Alexa Fluor 488 Conjugated Paxillin Antibody (CAT #C48774-AF488), with an excitation/emission gate 488/(510 – 550) nm. Myosin IIA was stained with Alexa Fluor 555-conjugated MYH9 antibody (CAT #C45364-AF555), with an excitation/emission gate 552/(565 – 590) nm, and palladin was stained with Alexa Fluor 594-conjugated PALLD antibody (CAT #C46119-AF594), with an excitation/emission gate 552/(605 – 630) nm. F-actin was stained with Alexa Fluor™ 633 Phalloidin (Thermo Scientific, Waltham, US) with an excitation/emission gate of 633/(647 – 700) nm. The nucleus was stained with 2 μg/mL Hoechst 33258 (Abcam, Cambridge, UK) at an excitation/emission gate of 405/(420 – 500) nm. The stained samples were finally mounted between slides and coverslips in ProLong™ Diamond Antifade Mountant (Invitrogen). The scanning was performed with a Leica TCS SP8 Confocal Microscope on the Leica LAS X software platform. A 40× water objective or a 63× oil objective and the Lightning mode on the software were utilized for imaging.

Topography of calcium deposits and element ratio analysis

The topography of calcium deposits on days 0, 7, 14, 21, and 28 of osteogenic differentiation was monitored by SEM imaging. Briefly, hBMSCs were fixed and dehydrated as mentioned above. The samples were then sputtered with gold and imaged on a Zeiss Sigma 500 VP Field-emission SEM with an SE2 detector operated at 1 kV in vacuum. Samples for element ratio analysis were not gold-treated. The Ca/Tb and Tb/(Ca + Tb) ratios of HAp coatings on day 0 or calcium deposits on day 28 were analyzed using SmartEDX embedded in the same SEM instrument under identical operation conditions as those used for topography imaging.

Osteopontin (OPN) and calcium deposit staining

OPN of hBMSCs on days 0, 7, 14, 21, and 28 of osteogenic differentiation was visualized. After 4% PFA fixation, 0.1% Triton permeabilization, and 1% bovine serum albumin (BSA) blocking, OPN was stained with the OPN Rabbit Anti-human Primary Antibody (Proteintech, Manchester, UK) at 1:200, followed by staining with the Alexa Fluor 594 Conjugated Mouse anti-Rabbit IgG (H+L) Secondary Antibody (Invitrogen) at 1:200, with an excitation/emission gate 552/(605 – 635) nm. The F-actin was stained with Alexa Fluor 488 phalloidin at an excitation/emission gate of 488/(510 – 550) nm. The nuclei were stained with 2 μg/mL Hoechst 33258 (Abcam, Cambridge, UK) at an excitation/emission gate of 405/(420 – 500) nm. Calcium deposits were stained with the OsteoSense 680EX (Perkin Elmer, Beaconsfield, UK) at 0.08 nmol/mL, 4°C overnight. The stained samples were finally mounted between slides and coverslips in the ProLong™ Diamond Antifade Mountant, and imaged using a Leica TCS SP8 Confocal Microscope with a 63× oil objective lens.

Surface information of coatings and calcium deposits characterized by atomic force microscopy (AFM)

To acquire the surface modulus, adhesion force, and roughness, the AC240TS-R2 cantilevers (Asylum Research, CA, USA) with silicon tips that have a radius of 25 nm and a spring constant of 2.8 N/m were used. On the Contact Mode Force, the imaging mode of AC contact was first used for topographical imaging. A 20 μm × 20 μm area of each coating sample was scanned at 0.5 Hz, with a set point of 1 V, and a resolution of 256 pixels. Three different topographical areas were obtained from each sample. The surface roughness (*Rq)* was defined as the root mean square average of the heights within the selected area. Three 12 μm^2^ areas from the aforementioned height maps were selected for calculating the average surface roughness (*Rq)*. After that, to obtain surface mechanical data, the imaging mode was switched to the contact mode. The same areas used in topography scanning were utilized for force mapping of 1024 points (32 × 32 points). A trigger point of 20 nN was set for all samples. Three random points from each force map (three force maps, nine points in total) were then selected for calculating the average surface modulus and adhesion force. The Derjaguin-Muller-Toporov (DMT) model was adopted for calculation. All imaging and calculations were carried out using the software MFP3D 15.09.112 (Asylum Research, CA, USA) integrated with Igor Pro 6.37 (WaveMetrics, Lake Oswego, USA).

Calcium deposit section and visualization using STEM and high-resolution transmission electron microscopy (HRTEM)

To visualize the inner structure of calcium deposits, the samples were embedded in resin and sectioned. The calcium deposit section view and the crystal lattice were observed using either STEM (500 VP FE-SEM, Zeiss, Germany) or HRTEM (JEM-2100F, JEOL, Tokyo, Japan) with an EDX detector. Briefly, after 28 days of osteogenic differentiation, the hBMSCs were fixed and dehydrated as described above. The cell-laden substrates were peeled off from coverslips and vertically placed at the bottom of 0.2 mL microcentrifuge tubes, embedded in Epon (Sigma‒Aldrich, Missouri, USA). After polymerization, the samples were then sectioned utilizing a Leica Reichert Ultracut S Ultra microtome (Leica, Wetzlar, Germany) with an ultra 45 diamond knife (DiATOME, Nidau Switzerland) at a speed of 1.0 mm/s and a thickness of 80 nm. Sections were collected onto carbon film-coated 200 mesh copper grids (Agar, Essex, UK). A gross section view of calcium deposits was visualized using a STEM detector at 30 kV. The crystal lattice was then visualized on the HRTEM at 200 kV and fast Fourier transform (FFT) diffractograms were generated. The element mapping of calcium deposits was conducted through an integrated EDX detector at 20 kV. ImageJ software was utilized to confirm lattice planes by measuring *d*-spacing after FFT and IFFT processing.

Visualization of extracellular matrix (ECM) via multiphoton (MP) microscopy

To visualize the arrangement of ECM on different coatings, a Leica TCS SP8 MP system was utilized to image type I collagen. The hBMSCs in the control, MD, and UD HAp groups on day 28 of osteogenic differentiation were fixed and dehydrated as described above, followed by F-actin staining using Alexa Fluor 488 and nuclear staining using Hoechst 33258. A pump laser of the MP microscope with a wavelength of 910 nm was used to excite the second harmonic generation (SHG) signal of ECM, which was collected by the PMT Trans detector. At the same time, the signal of F-actin (Alexa Fluor 488) was collected by the HyD SMD 2 detector. Then, the pump laser was switched to a wavelength of 710 nm, and signals of the nucleus (Hoechst 33258) were collected by the HyD SMD 2 detector.

Bulk RNA extraction, sequencing (RNA-Seq), and bioinformatic analysis

See Supplementary **Note** 3 for elaboration.

siRNA transfection

hBMSCs at passage 6 were seeded on coverslips with different coatings at a density of 2.5 × 10^4^ per well in 48-well plates. Six groups were set up: control (–), control (+), siRNA control (–), siCOL1A1, siCOL4A6, and siCOL1A1 + siCOL4A6. These groups represent PDMS-coated coverslips, UD HAp NRs on PDMS, UD HAp NRs on PDMS and scramble siRNA/siCOL1A1/siCOL4A6/siCOL1A1 + siCOL4A6 applied. Cells were cultured in 300 μL MSCM without antibiotics for the first day to ensure attachment. On the transfection day, 20 pmol siRNA (COL1A1, COL4A6, COL1A1+ COL4A6, or scramble control siRNA) and 1 μL Lipofectamine 2000 (Invitrogen) were separately diluted in 50 μL Gibco Opti-MEM I Reduced Serum Medium (Fisher Scientific) without serum and then mixed and added into each well, according to the manufacturer’s instructions. After incubation at 37°C and 5% CO2 for 24 h, the oligomer-Lipofectamine 2000 complexes were replaced with 300 μL MODM without antibiotics and cultured until the next transfection or the endpoint. The siRNA transfection was carried out on days 0, 7, 14, and 19 of osteogenic differentiation for western blot, PCR analysis, and fluorescence staining (performed on day 21) or on days 0, 7, and 12 of osteogenic differentiation for the ALP activity assay and total DNA measurement (performed on day 14). The siRNAs were fabricated by Invitrogen, and the sequences used in this experiment were as follows: siRNA targeting COL1A1 (siCOL1A1, forward 5’-GCGAUGACGUGAUCUGUGAtt-3’ and reverse 5’-UCACAGAUCACGUCAUCGCac-3’), siRNA targeting COL4A6 (siCOL4A6, forward 5’-AGGAUCUAGUGGUCCAUAUtt-3’ and reverse 5’-AUAUGGACCACUAGAUCCUgg-3’), and Silencer Select Negative Control No. 1 siRNA (scramble control).

Western blot after RNAi

Western blotting was performed to evaluate the effectiveness and impact of RNAi on hBMSCs on the UD HAp NRs by verifying the expression levels of COL1A1 and COL4A6 and the activation/deactivation of the PI3K-Akt signaling pathway. The XCell SureLock Mini-Cell Electroblotting Unit, anti-COL1A1, anti-COL4A6, anti-PI3k p85α, anti-phospho-PI3k p85α, anti-Akt, anti-phospho-Akt, and anti-GAPDH (all rabbit anti-human), and all other reagents and consumables for western blotting were purchased from Thermo Fisher Scientific (Waltham, US) unless otherwise specified. The cells were lysed using a cell lysis buffer (Invitrogen, Waltham, US) supplemented with protease and phosphatase inhibitor mini tablets, following the manufacturer’s instructions. The total protein concentration of lysates was then measured using a Rapid Gold BCA protein assay kit, following the manufacturer’s instructions. After that, a loading of 2 μg total protein was mixed with the NuPAGE LDS Sample Buffer (4X) with supplementary distilled water to reach a final volume of 10 μL. After incubation at 70°C for 10 min to denature proteins, 10 μL of each sample was loaded into wells of NuPAGE 10-well 4 to 12% Bis-Tris Mini Protein Gels and underwent electrophoresis in NuPAGE MES SDS Running Buffer. The electrophoresis program was set at 200 V for 35 min. When finished, the gel was transferred to a PVDF membrane sandwich in Blot Buffer and blotted at 20 V for 60 min. After that, the membrane was thoroughly washed using TBST and then incubated in blocking buffer with primary antibodies at 4°C overnight. The membrane was then incubated with HRP-linked goat anti-rabbit IgG (H+L) secondary antibody and the SuperSignal West Pico PLUS Chemiluminescent Substrate for imaging. The imaging was carried out on the ChemiDoc XRS+ Gel Imaging System (Bio-Rad Laboratories, California, USA).

Supplementary Text

**Note** S1:

It is known that amorphous calcium phosphate (ACP) and ordered nanocrystalline HAp coexist in bone, and the amorphous phase is regarded as a transient precursor of nanocrystalline HAp^[6]^. Some hypotheses of biosynthesized hydroxyapatite (BHAp) formation have been proposed: ACP precursor clusters agglomerate and recrystallize into BHAp^[7]^ or transform into BHAp via the octacalcium phosphate (OCP)^[8]^ pathway. In this study, EDX analysis confirms that the deposits are rich in Ca, P, and O elements (**Figure** S20A). Then, HRTEM was performed to reveal the crystal lattice structure of calcium deposits. The distance (*d*-spacing) between periodic lattice fringes helps in comparison with the JCPDS standard data and the identification of lattice planes. The employment of fast Fourier transform (FFT) on all periodic lattice fringes resulted in diffractograms of the entire HRTEM image with sharp spots, on which the inverse FFT (IFFT) was applied to regenerate clearer lattice fringes of the selected lattice plane. By using this method, lattice fringes belonging to different planes can be isolated, and their location, intensity, orientation, and *d*-spacing in the original HRTEM images can be confirmed. Calcium deposits on day 28 in the control, MD and UD HAp groups were investigated. In **Figure** S20B2, S20B9, and S20B16, lattice fringes can be observed in most of the image areas in all groups. Notably, a number of dense granules were found in the control and MD HAp groups. The grain size of the calcium deposit was about 10 **–** 20 nm in the control and MD HAp groups. The crystal grains consisting of defect grain boundaries normally have more complicated diffractograms than those of individual crystal grains, nevertheless larger uniform lattice planes of well-crystallized domains were identified in the MD HAp group (**Figure** S20B12 and S20B13) than in the control group (**Figure** S20B5 and S17B6). One lattice plane in the control and MD HAp groups also has multiple orientations, evidenced by more sharp spots on the same diffraction ring (**Figure** S20B3 and S20B10). Specifically, the orientation of the (002) plane in the control and MD HAp groups shows no preference, and there are dispersed weak (002) domains which can be identified (**Figure** S20B4 and S20B11). Combining the IFFTs showing deformed lattice of other planes, for example, (212) and (301) planes (**Figure** S20B5, S20B12), it is evident that the calcium deposits in both control and MD HAp groups are BHAp polycrystals with significant deformation. In the UD HAp group, there are no significant dense granules, and fewer crystal grain defect boundaries are observed (**Figure** S20B16). Figure S20B17 demonstrates that the orientation of (002) plane largely follows that of UD HAp NRs substrate. The IFFT of (002) shows uniform and interconnected fringes in a broad area (**Figure** S20B18). Although the deposits in the UD HAp group are still polycrystals as more than one orientation can be observed on (002) and other planes (**Figure** S20B18 – S20B20), there is a less lattice deformation and more consistent orientation. Importantly, the anisotropic diffraction arcs and fringes of (101) plane exhibit high uniformity on a large scale (**Figure** S20B17 and S20B21). In the HRTEM of UD HAp group (electron beam was horizontal to the sample surface), (002) plane can be identified but neither (200) nor (300) plane can be clearly seen. In contrast, (200) and (300) planes can be found in the UD HAp group with increased intensity in day 28 XRD data (electron beam was perpendicular to the sample surface) but not the (002) plane (**Figure** S21A). These findings strongly suggest that the deposit has a preferred growth direction along the same direction of the c-axis of the UD HAp NRs substrate. Based on the results above, three conclusions can be drawn: 1) the deposits in UD HAp group are directional and follow the orientation of UD HAp NRs substrate; 2) The deposits in different groups are all well-crystallized polycrystalline BHAp. In addition, 3) the grains in all groups display some interconnected domains and an aggregating trend. This agrees with previous reports of the nonclassical crystallization of biominerals, which describe the growth of secondary particles at the expense of primary particles via oriented attachment or self-assembly^[9]^. Our findings support the hypothesis that BHAp is formed via precursor cluster agglomeration and recrystallisation, although more work is needed to verify the OCP pathway hypothesis. Our work does not include observation at multiple time points to further clarify the agglomeration and recrystallization behavior. A dynamic observation with more time points is planned.

**Note** S2:

This note elaborates the methods and results of RNA-seq and bioinformatics analysis. RNA extraction was performed on day 21 of osteogenic differentiation of the control, MD, and UD HAp groups (three biological replicates for each condition) using a Monarch Total RNA Miniprep Kit (New England Biolabs, Ipswich, US) according to the manufacturer’s protocol. RNA-Seq was carried out at the UCL Genomics facility. The RNA concentration was measured on a Nanodrop spectrometer, and 500 ng of RNA was used for cDNA synthesis and library preparation using the Kapa mRNA HyperPrep assay (Roche, Basel, Switzerland) and xGen UDI-UMI adapters (Integrated DNA Technology, Shanghai, China). Sequencing of these libraries was performed on a NextSeq 2000 (Illumina, San Diego, US) P2 flow-cell with 56 bp paired-end reads. The as-obtained RAW FASTQ files were used for further bioinformatic analysis. The flowchart of RNA-Seq and bioinformatic analyses is shown in **Figure** S25.

*3.1 Preprocessing of RAW data*

The RAW FASTQ files were uploaded onto the Galaxy platform (https://usegalaxy.org/), which allows simplified coupling of external data resources with hundreds of popular data analysis tools^[10]^. A series of tools integrated into the Galaxy platform were utilized for preprocessing. Briefly, sequencing quality control was carried out with FastQC v.0.11.9, and then the adapters and poorly expressed or non-expressed genes were trimmed via Trimmomatic v.0.38.0 with the following parameters: PE; ILLUMINACLIP: TruSeq3-PE.fa: 2:30:10; LEADING: 3; TRAILING: 3; SLIDINGWINDOW: 4:15; MINLEN: 50. After that, HISAT2 v.2.2.1 was utilized to map sequences to the UCSC Homo sapiens GRCh38 reference with an average mapping percentage of 90.42%. The table of read counts with Entrez gene IDs was generated using featureCounts v.2.0.1. Ultimately, 18457 out of the original set of 28396 genes remained after pre-processing. They were used for downstream bioinformatic analysis.

*3.2 Identification of DEGs*

The pre-processed data matrix was uploaded to NetworkAnalyst 3.0 (<https://www.networkanalyst.ca>) for DEG screening. This platform provides visual analytics for comprehensive gene expression profiling and meta-analysis^[11]^. A total of 18233 (98%) out of 18457 pre-processed genes were successfully matched with the built-in Homo sapiens Entrez ID of NetworkAnalyst. The log2 counts per million method was used for data normalization. **Figure** S26A shows the normalization results. The medians are aligned on a line, which indicates good quality and suitability of the data for the following analysis. **Figure** S26B demonstrates that the samples are distributed into three well-separated clusters, suggesting that the substrate surface treatments were applied successfully. The MD and UD HAp NR groups were closer to each other than to the control group, which indicates that they underwent a more similar gene expression pattern but were still not identical. The upregulated and downregulated DEGs between the UD HAp group and the control group were identified by the Limma package. The p value was corrected using the Benjamini‒Hochberg test, and the threshold of DEGs was set as log2-fold change |log2FC| > 2.0 and adjusted P < 0.05. A total of 220 DEGs, including 135 (61.4%) downregulated genes and 85 (38.6%) upregulated genes (**Figure** S27A and S27B), were identified in the UD HAp group. The obtained table containing identified DEGs was downloaded for the following analysis. A Venn diagram of DEGs (**Figure** S27C) was plotted using the online Venn diagram creator from Creately (https://creately.com/) to identify the shared genes between groups.

*3.3 GO and pathway enrichment analyses*

The g:Profiler (<http://biit.cs.ut.ee/gprofiler/>) is an open-access web server for characterizing and manipulating gene lists resulting from mining high-throughput genomic data^[12]^. In this study, the list of DEGs was uploaded onto g:Profiler, and the GO terms and KEGG pathway enrichment analyses of DEGs were carried out via g:GOSt on g:Profiler. The optimized g:SCS algorithm^[12]^ was adopted as the threshold since it considers the hierarchical structure of terms of each organism and therefore gives a tighter threshold to significant results. Additionally, P < 0.05 was applied to the cut-off criteria. The GO term enrichment describes the DEG set from three aspects, including biological processes (BP), cellular component (CC), and molecular function (MF). The significantly enriched signaling pathways were based on the KEGG database. Irrelevant disease pathways in the KEGG pathway enrichment analysis were removed before analysis. **Figure** S28 and **Table** S10 summarize the top enrichment terms of DEGs of the UD HAp group.

*3.4 PPI network construction, hub gene identification, and enrichment analysis*

The PPI network recapitulates the interaction between proteins and thus depicts the relationship between their encoding genes. To understand the mechanism of directional osteogenesis on the UD HAp NRs, the DEG list was first uploaded onto the STRING (<https://string-db.org/>) database^[13]^ to generate an interaction map of proteins coded by DEGs. A confidence score of >0.4 was defined as significant. After that, the interaction data were downloaded and utilized for PPI network visualization via Cytoscape software (version 3.8.0, Cytoscape Consortium, San Diego, CA, USA). **Figure** S28 demonstrates that the PPI network consisted of 84 nodes and 167 edges. Degree was defined as the number of connections between different proteins in the network and utilized for ranking the top 10 hub genes by the plug-in cytoHubba. Based on this, the genes MMP2, COL1A1, CD4, COL11A1, ELN, ITGB3, BGN, SPARC, NID2, and COL4A6 were identified as hub genes (**Table** S11). The 10 hub genes then underwent GO and signaling pathway enrichment analysis to reveal their functions and involved pathways through g:Profiler. Similarly, irrelevant disease pathways in the KEGG pathway enrichment analysis were not considered in the analysis. **Figure** S30 and **Table** S12 summarize the top enrichment terms of 10 hub genes of the UD HAp group. As a result, the ECM-related BP GO terms and three pathways (ECM-receptor interaction, Relaxin signaling pathway, and Focal adhesion) were found to be highly enriched. Venn diagrams of hub genes involved in signaling pathways were plotted using Creately (<https://creately.com/>). The shared hub genes among the above three enriched pathways were identified as the pivotal genes involved in the signaling pathways. Ultimately, COL1A1 and COL4A6 were selected as targets for the following verification experiments (**Figure** S32).

**Note** S4:

In western blot, weakened or eliminated bands in siCOL1A1 and siCOL4A6 groups confirm effective RNAi of COL1A1 and COL4A6. The hBMSCs on UD HAp NRs show enhanced expression of p-PI3K and p-Akt. In comparison, reduced expression of these two phosphorylated proteins and poor cytoskeleton tension was observed in the siCOL1A1 and/or siCOL4A6 groups, especially the siCOL4A6 group and the double-silenced group. COL4A6 seemed to be synergistic with but play a more critical role than COL1A1, as the siCOL4A6 group had more significant adverse conditions than the siCOL1A1 group. This effect was enhanced when both COL1A1 and COL4A6 were silenced. The COL family comprises many types of molecules and is the most abundant protein in the ECM^[14]^. However, to date, it remains elusive which COL is relevant to the formation of ordered bone structure. Based on current knowledge, COL4 is a major component of basement membranes. In the bone marrow, it is found in the endosteal region as well as the central marrow^[15]^. The basement membranes regulate cell adhesion, proliferation and differentiation. Additionally, it can also regulate cell polarity and directed migration via their effect on the cytoskeleton^[16]^. COL1 functions in the early event of stem cell osteogenic differentiation ^[17]^ and is also the most abundant type of collagen in the bone ECM. From disordered callus to ordered healthy bone structure, bone remodeling is the process responsible for replacing disordered old bony tissues with ordered new bone. The resorption-deposition balance highly depends on the activity of osteoblasts and osteoclasts, and it is evident that ECM as a network of signaling has a key role in regulating bioactivities of the osteoblasts and osteoclasts^[18]^. Our bioinformatic analyses on the RNA-Seq support that COL1A1 and COL4A6 may be the key genes associated with the formation of ordered calcium deposits. The COL1A1 was upregulated while the COL4A6 was downregulated in the UD HAp group. In comparison, the Western blot showed conflicting results of both increased COL1A1 and COL4A6 proteins in the UD HAp group. This discrepancy between the transcriptome and proteome of COL4A6 can be explained by the detection time point. The COL4A6 mRNA in the UD HAp group may reach a quantity of accumulation peak and translate the protein earlier than the other conditions, and then the COL4A6 mRNA may have already degraded when detected on day 21. Alternatively, there might be some unknown posttranscriptional processing that can lead to similar results, which needs further investigation. COL1A1 and COL4A6 are genes involved in the PI3k-Akt signaling pathway, which is a subclass of the Focal adhesion pathway. The PI3k-Akt signaling pathway is known to be critical in osteogenesis^[19]^ and can be activated by many factors, such as the aforementioned ECM/integrin-mediated transmembrane signaling regulation^[20]^, microRNAs (miRNAs)^[21]^ and mechanical stress^[22]^. Our findings support the hypothesis of UD HAp NR-induced ordered calcium deposition via the COL1A1- and COL4A6-mediated PI3k-Akt signaling pathways.


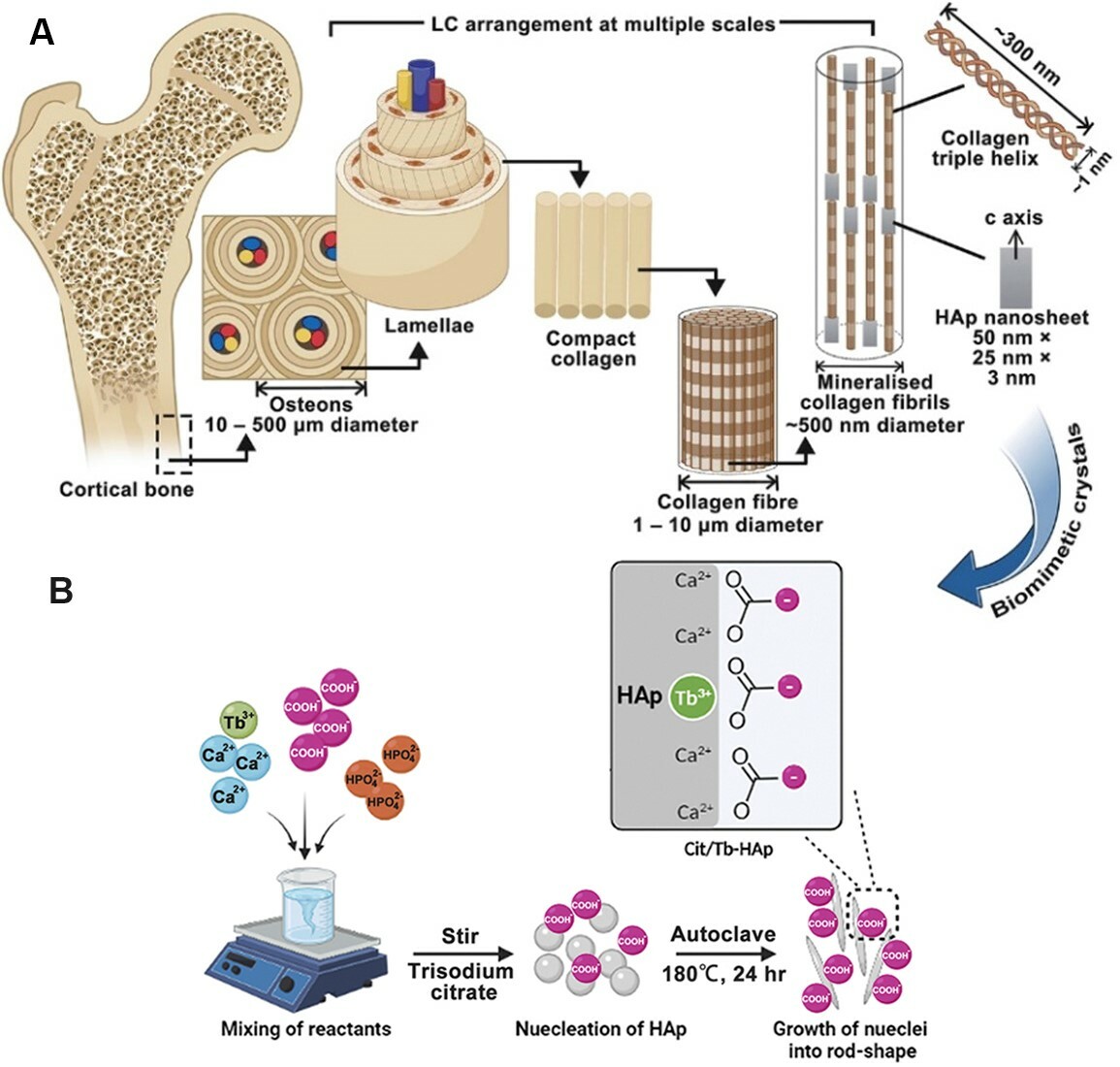


Figure S1. Schematic of liquid crystalline structures in bone at multiple scales and synthesis of biomimetic crystals. (A) Macro- to nano-structure of native bone shows liquid crystalline (LC) arrangement at multiple scales. (B) The typical synthesis process of Cit/Tb-HAp NRs. The magnified figure illustrates that Tb^3+^ substitutes the ion site of Ca^2+^ in the lattice, and trisodium citrate acts as template to facilitatecrystal growth into nanorods and the carboxyl from citrate bonds onto the surface of HAp.^[23]^


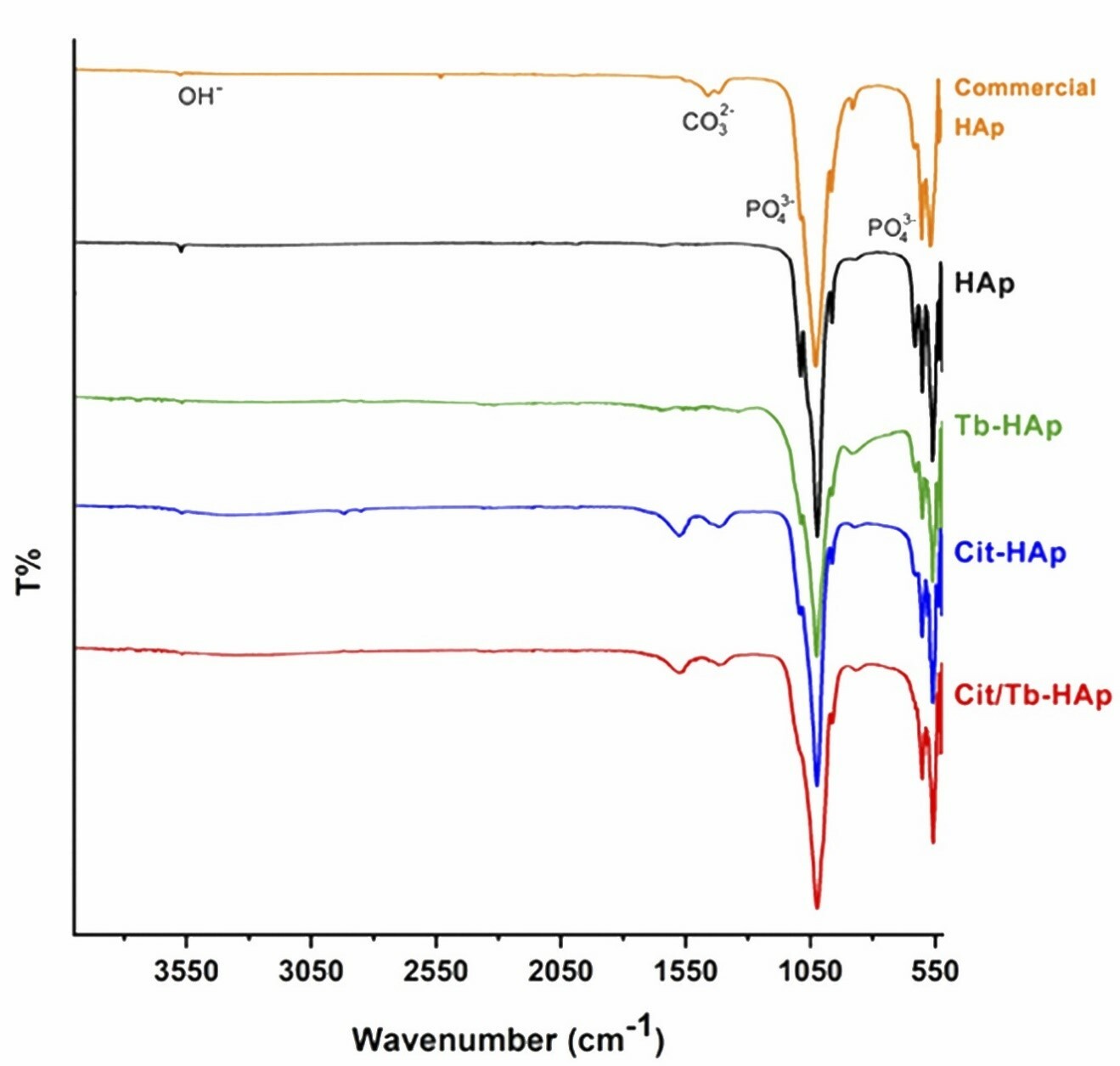


Figure S2. Fourier-transform infrared spectroscopy (FTIR) of HAp samples.

The FTIR spectrum of HAps. In all samples, the most significant peaks are observed at 1084 and 1025 cm^-1,^ indicating ν_3_(PO_4_^3−^) asymmetric stretching vibrations. The peak at 961 cm^-1^ matches ν_1_(PO_4_^3−^) symmetric stretching vibrations, and the peaks observed at 600 and 566 cm^-1^ confirm the presence of ν_4_(PO_4_^3−^) asymmetric bending vibrations. Small peaks corresponding to the stretching and bending vibrations of CO_3_^2−^ groups at 1455, 1415 and 877 cm^−1^ indicate a trace amount of CO_3_^2−^ groups resulting from the absorption of carbon dioxide from the atmosphere during synthesis^[24]^. The narrow peak at 3570 cm^−1^ indicates the existence of characteristic OH^-^ groups of HAp. Apart from the above functional groups existing in all samples, in Cit-HAp and Cit/Tb-HAp samples, the peak at 1576 cm^−1^ is attributed to the addition of Cit^3−^ ions^[24]^.


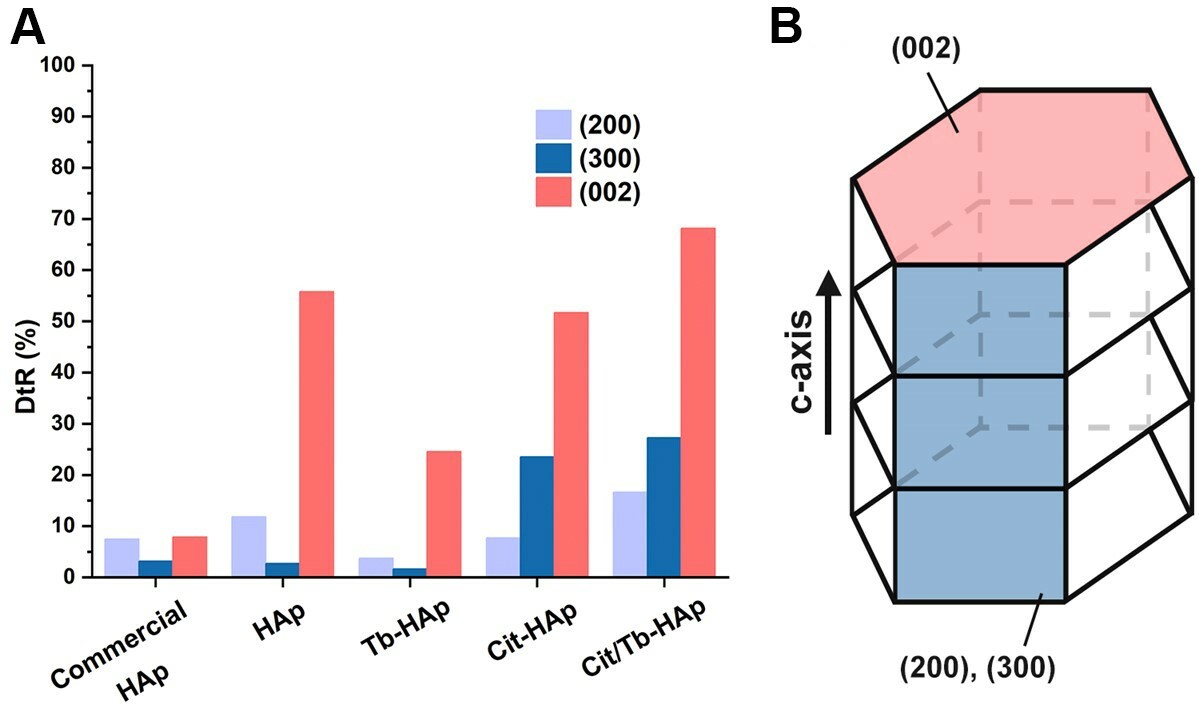


Figure S3. Growth Preference of different HAp samples.

**
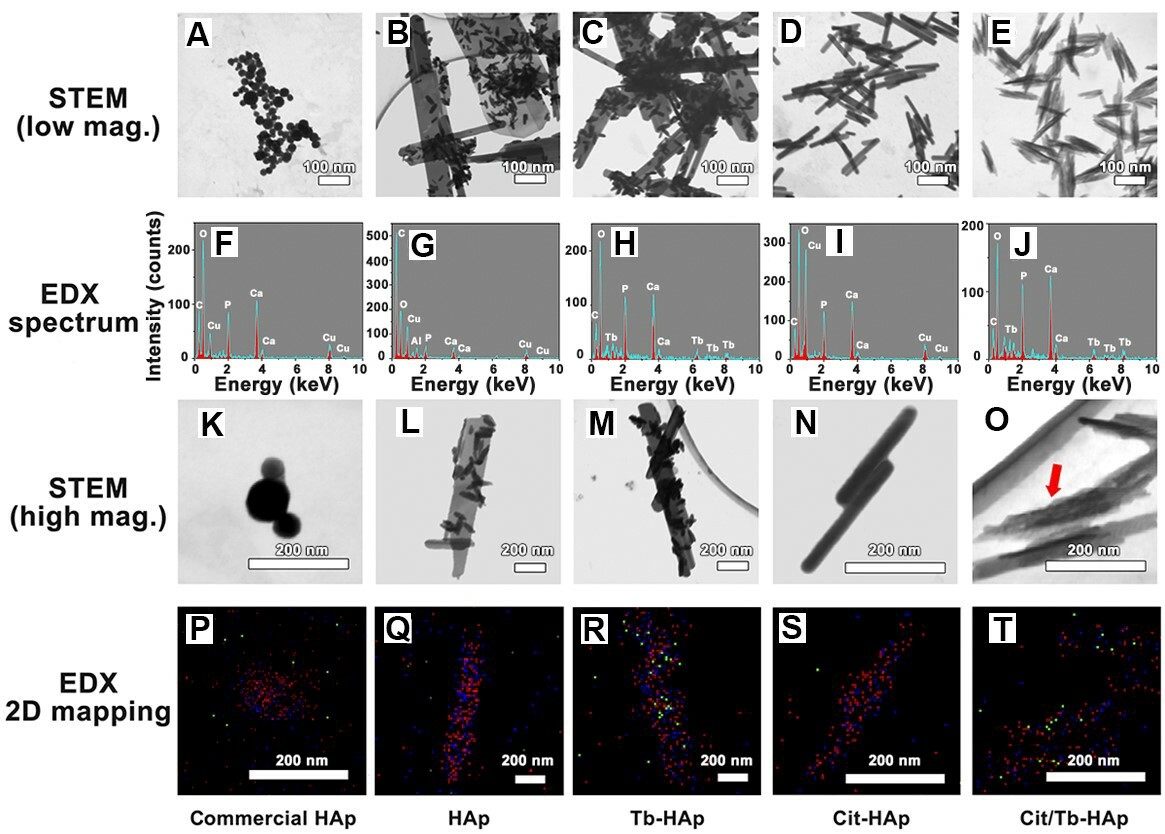
**(**A**) The *DtR* quantification analysis shows that all synthesized Haps have overwhelmingly higher *DtR*(002) than *DtR*(200) and *DtR*(300), despite different formulas. (**B**) Correlation between HAp NR growth direction (c-axis) and lattice planes (200), (300) and (002).

**Figure S4. STEM imaging and EDX analysis of HAp samples.**

(**A** – **E**) Low magnification STEM view of HAp samples. Scale bar = 100 nm. (**F** – **J**) EDX spectrum analysis of HAp samples. (**K** – **O**) High magnification STEM view of HAp samples. The red arrow indicates the surface defects of Cit/Tb-HAp. Scale bar = 200 nm, (**P** – **T**) EDX 2D element mapping of HAp samples in the high magnification STEM view. Scale bar = 200 nm.

**
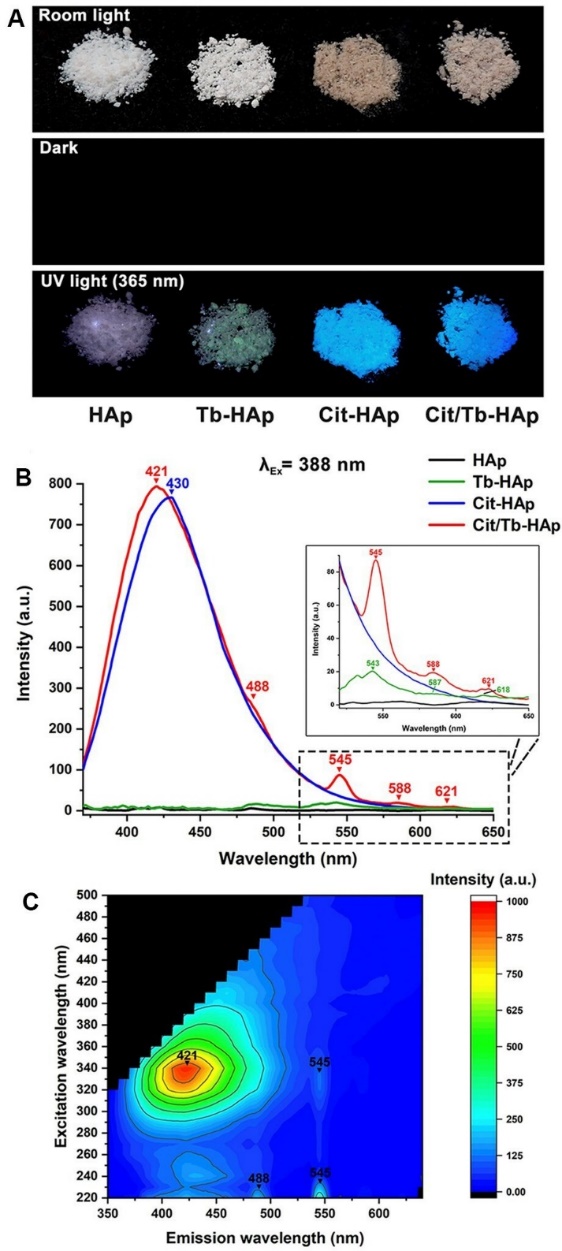
Figure S5. Fluorescence analysis of HAp samples.**

(**A**) Macroscopic view of HAp powders under room light, in the dark, and under 365 nm UV light. (**B**) Full emission spectra of HAp samples excited by 388 nm wavelength. The magnified view shows the fluorescence peaks attributed to terbium. (**C**) 2D heatmap of the full excitation/emission spectra of Cit/Tb-HAp.


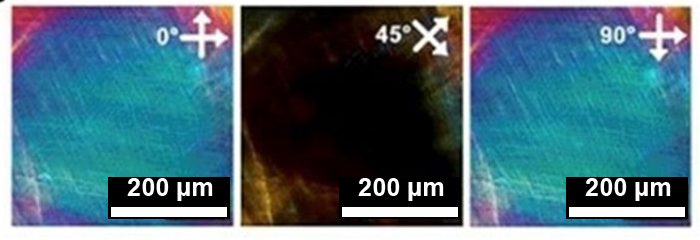


**Figure S6. High magnification crossed polarized view of Cit/Tb-HAp NR LC colloid (35 wt%).** Exchange of bright and dark domains of a 35 wt% HAp NR LC sample in cuvette rotated every 45° to the analyser. Scale bar = 100 μm.


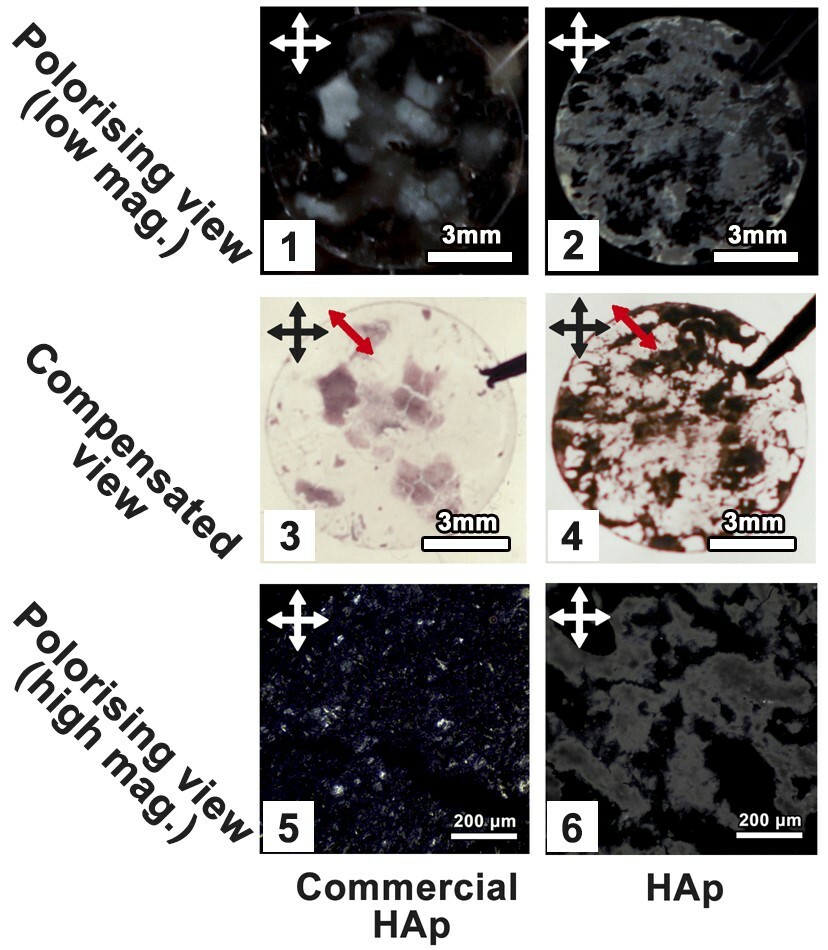


**Figure S7**. Cross-polarized view of coated commercial HAp and HAp coverslips.

Low magnification polarizing (**1** and **2**, scale bar = 3 mm) and compensated views (**3** and **4**, scale bar = 3 mm) were taken between cross polarizers (directions of black/white crossed arrows), with or without a 1/4λ wavelength compensator (direction of red arrows) that was 45° to the analyzer. The polarizing view at a higher magnification (**5** and **6,** scale bar = 200 μm) was taken using a cross-polarized microscope. The weak birefringence is attributed to the crystal structures of HAp aggregates but not the long-range orientational structure of Hap LC. The dark areas represent no sample areas due to nonuniform coating and remained the same when cross polarizers rotated.


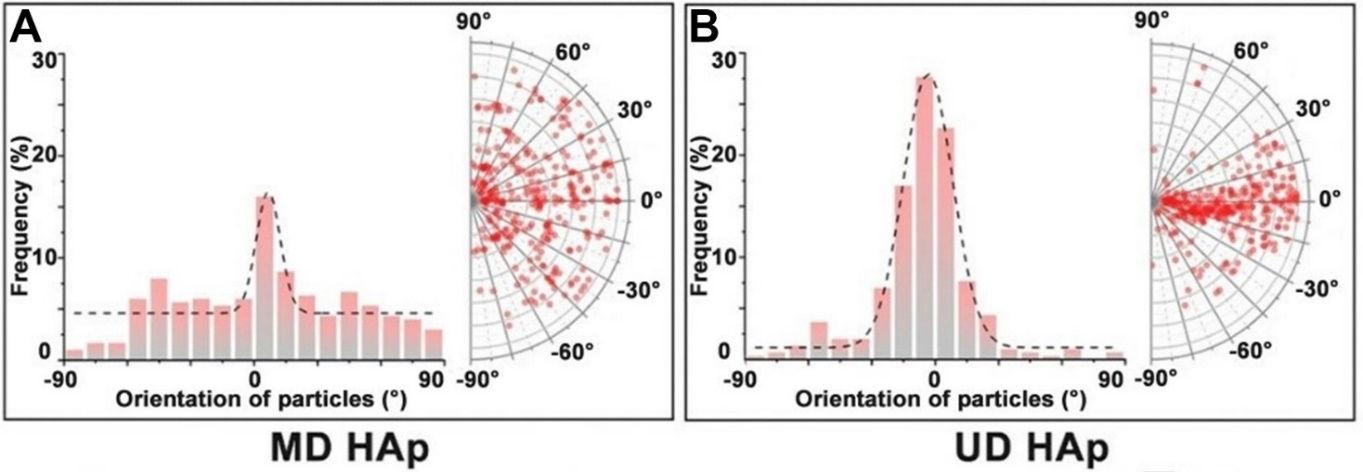


**Figure S8.** **The statistics of particle orientation.** (**A**) MD HAp NRs and (**B**) UD HAp NRs. Each dot represents a particle, *n* = 300.


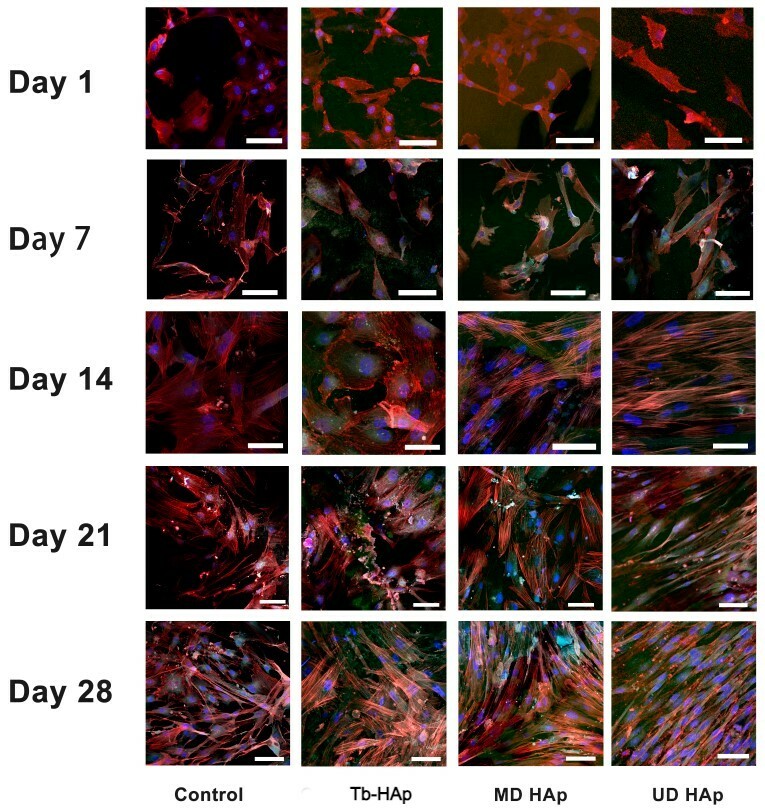


Figure S9. Immunocytochemistry (ICC) images of hBMSCs in different substrate groups during osteogenic differentiation by confocal microscopy.

On the MD HAp, cells can only maintain the same orientation of elongation within tens or hundreds of microns, then alter their preferred orientation induced by the local alignment of HAp NR within randomly oriented multi-domain structures. Their local preference is not controllable. In contrast, the orientation of elongated cells on UD HAp is more controllable, stretching at a larger scale over several millimeters and above, as long as the orientation of UD HAp substrate is maintained. Red = F-actin; Blue = nucleus; Green = HAp substrates. Scale bar = 50 μm.


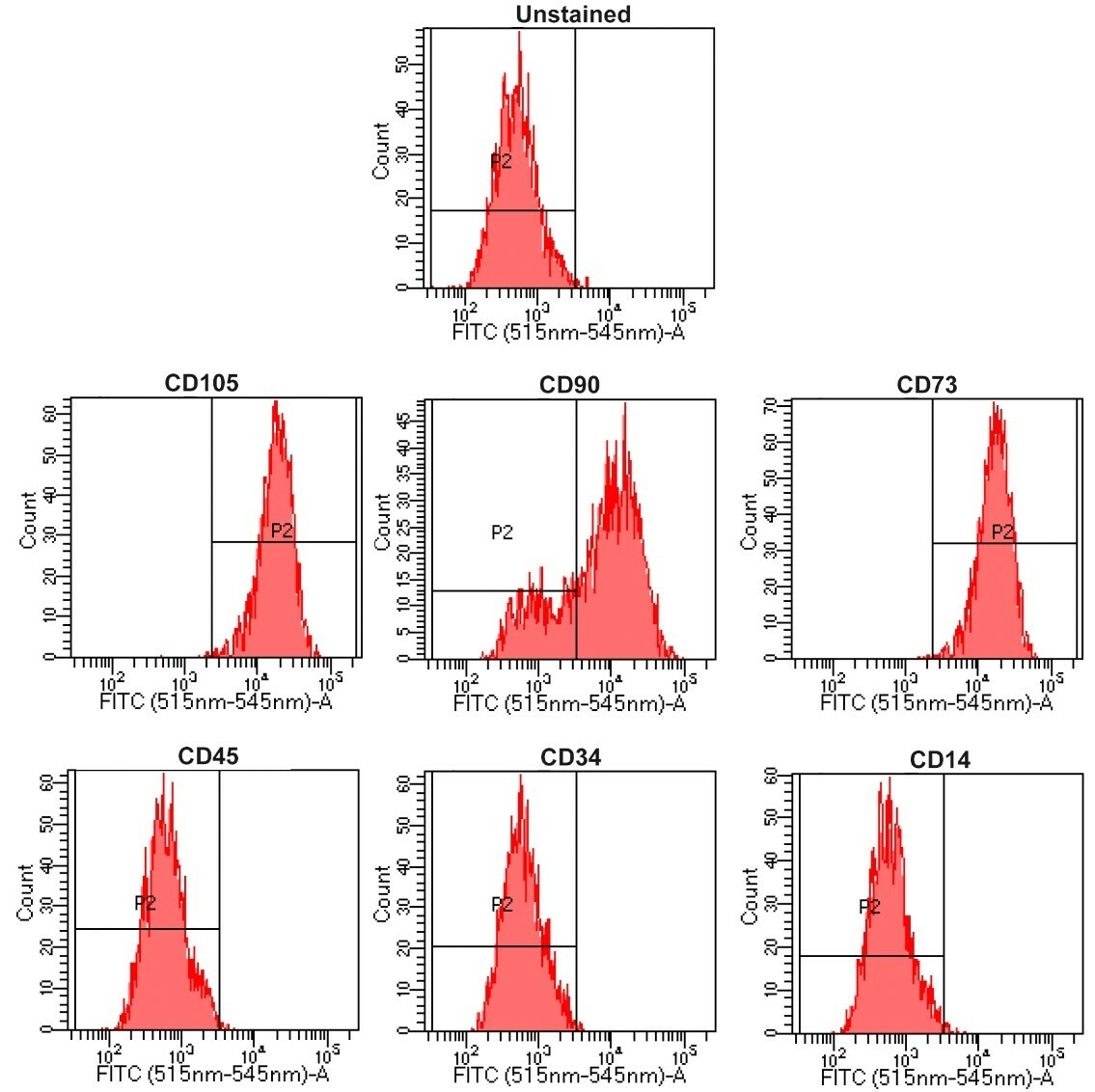


**Figure S10. Verification of pluripotency of hBMSCs via surface marker presence analysis.**

Briefly, cells at passage 4 and 80% confluence were harvested and resuspended in Hank's balanced salt solution (HBSS) with 2% FBS. Cells were then distributed into seven tubes at a density of 1 × 106 and stained with FITC-conjugated anti-human CD34, CD73, CD105, CD14, CD90, and CD45 antibodies (Biolegend, San Diego, CA, US) respectively according to manufacturer’s instruction, adding one unstained control sample. Triplicate samples were prepared for each antibody including unstained control. After incubation on ice for 30 min in the dark, cells were washed with 2 mL HBSS with 2% FBS and centrifuged at 1500 rpm for 5 min, followed by resuspension in 200 μL HBSS with 2% FBS. After that, cells were analyzed by flow cytometry utilizing a BD LSR II flow cytometer (BD Biosciences, NJ, US) with a BD FACSDiva Software. The cytometer was set to acquire 20,000 events for each tube. The cells were plotted using forward scatter (FSC) and side scatter (SSC) and gated to analyze single cells. The data on the cell surface markers was then collected. The results of flow cytometry confirm the presence of CD105, CD90, and CD73, while lacking expression of CD45, CD34, and CD14 in the hBMSCs which is in line with the definition of MSC by the Mesenchymal and Tissue Stem Cell Committee of the International Society for Cellular Therapy (ISCT).


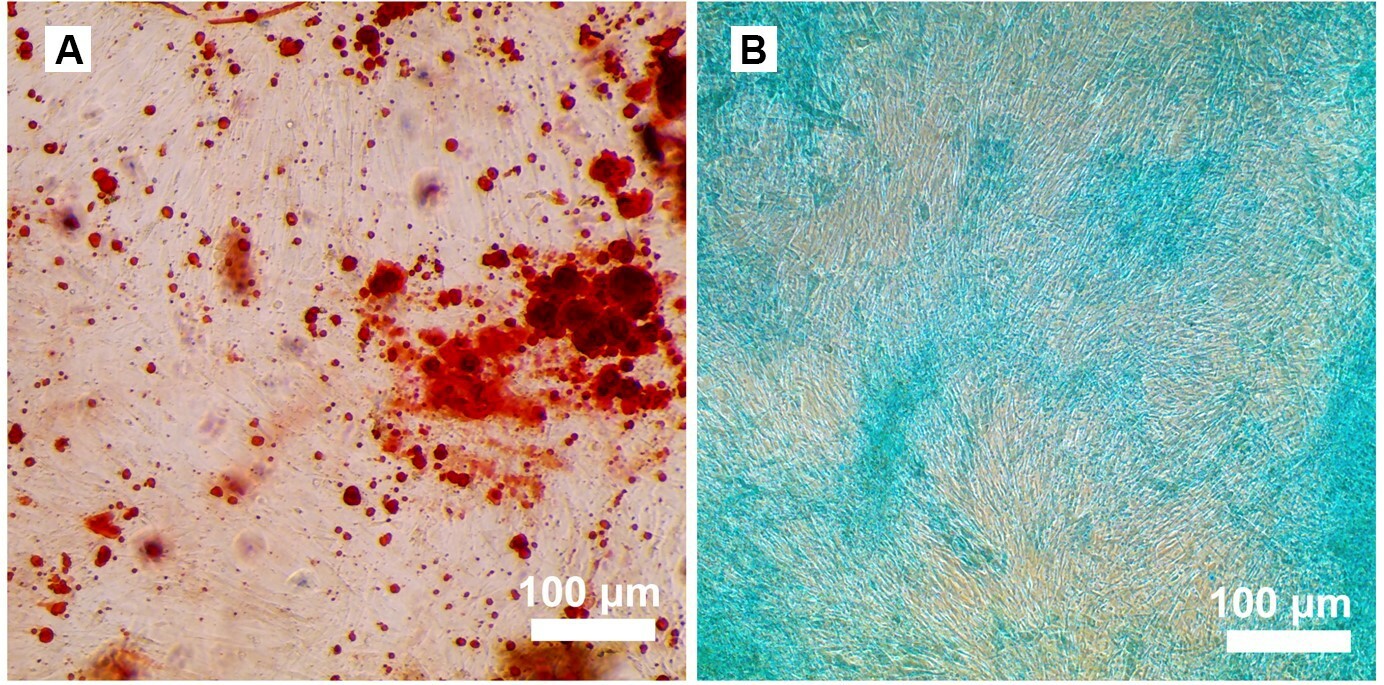
Figure S11. Verification of pluripotency of hBMSCs via bilineage differentiation (osteogenic and chondrogenic).

The hBMSCs at passage 4 and 80% confluence were harvested and seeded into T25 flasks at a density of 5 × 10^5^. Then they were culture in the MSCM for proliferation until reached 80% confluence again, followed by replacing medium with Mesenchymal Stem Cell Osteogenic Differentiation medium (MODM, CAT #7531) or Mesenchymal Stem Cell Chondrogenic Differentiation Medium (MCDM, CAT #7551). After 28-day differentiation, all the cells were washed with PBS and fixed with 4% paraformaldehyde (PFA) for 15 min at room temperature. The 2% Alizarin Red S Staining Kit (Catalogue #0223, Sciencell, Carlsbad, CA, US) and 1% the Alcian Blue 8GX (Sigma-Aldrich, Missouri, USA) acetic acid solution were utilized for visualizing calcium nodes (osteogenic differentiation) or glycosaminoglycans (chondrogenic differentiation), respectively. After 30-min staining, the flasks were rinsed with PBS for 5 times to wash off non-binding excessive reagents. The stained flasks were then imaged using an EVOS XL Core Imaging System with 20 × lens. The bilineage differentiation tests prove that the hBMSCs have the pluripotent capabilities of differentiating into either osteogenic or chondrogenic lineages, shown by (A) red staining of calcium deposit in osteogenic medium or (B) blue staining of glycosaminoglycans in chondrogenic medium. Scale bar = 100 μm.


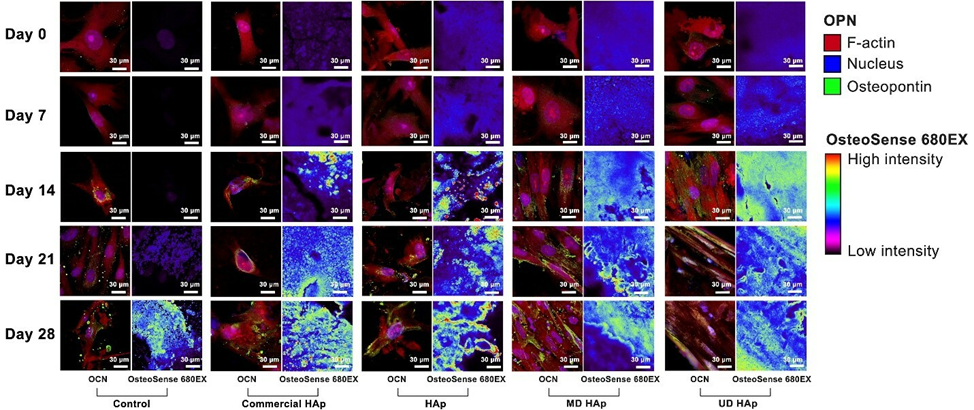


Figure S12. Confocal microscope imaging of osteopontin and calcium deposits.

During the 28-day osteogenic differentiation of hBMSCs, osteopontin and calcium deposits were stained for confocal microscopy imaging on days 0, 7, 14, 21, and 28. The results show that OPN appears from day 14 of differentiation in all groups and is consistently present until the end of osteogenic differentiation. The presence of OPN indicates initiation and maturation of osteogenic differentiation of the hBMSCs at different time points. More importantly, the calcium deposit is initially found using OsteoSense 680EX on day 14. The calcium fluorescence intensity steadily increases until aligned intensive calcium staining is observed on day 21 and 28 in the UD HAp group. For OPN staining, red: F-actin; blue: nucleus; green: OPN. For calcium deposit staining using the OsteoSense 680EX, red indicates high fluorescence intensity, while black stands for nonfluorescence in the spectrum. Captured using a 63× oil objective. Scale bar = 30 μm.

**
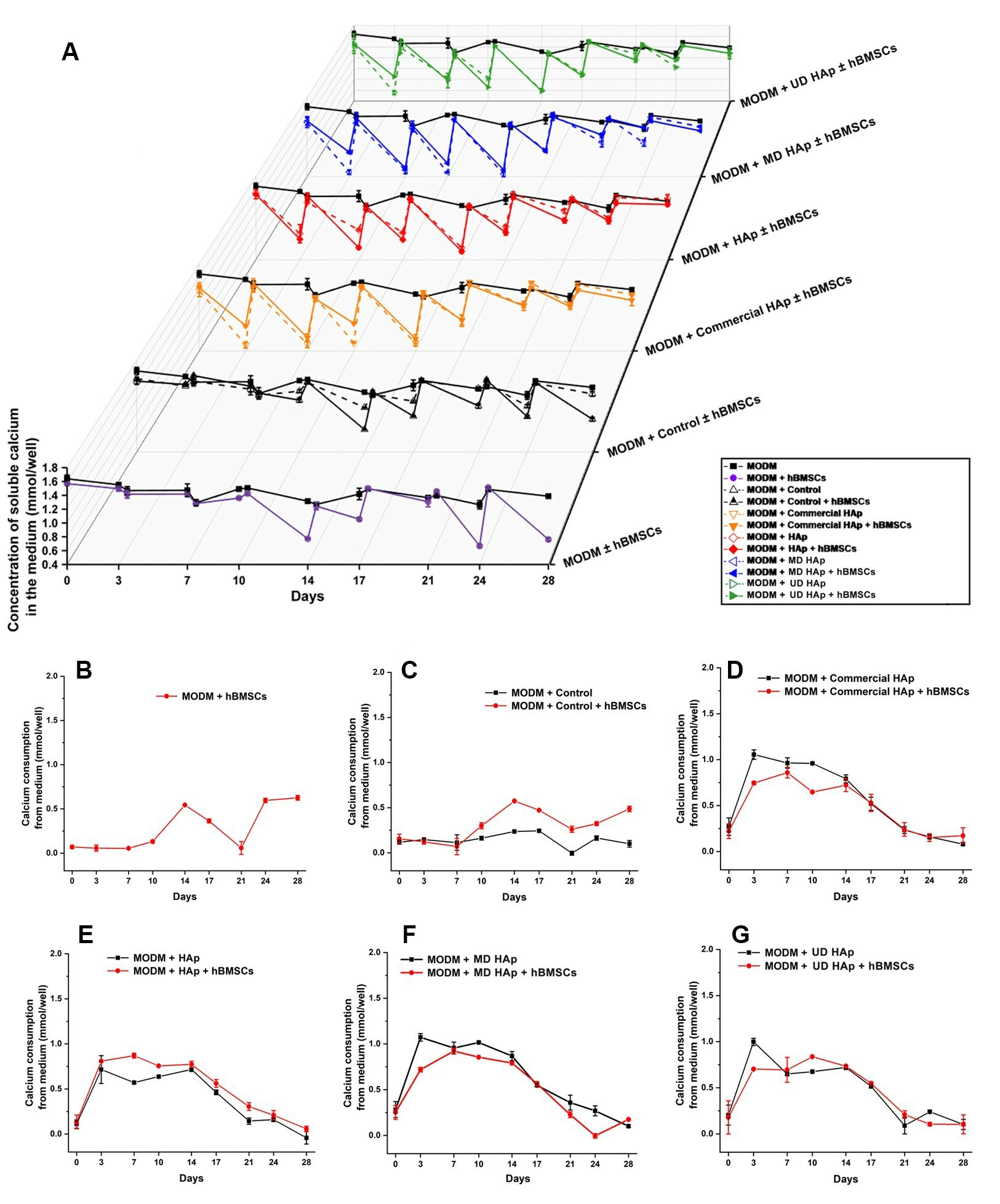
**

Figure S13. Free calcium ion kinetics in osteogenic differentiation medium.

**(A)** Overall concentration of soluble calcium ions in the medium throughout 28-day osteogenic differentiation culture. (**B – G)** Calcium consumption of each group from the medium throughout the 28-day culture, converted from (**A)**. Each condition *n* = 3.


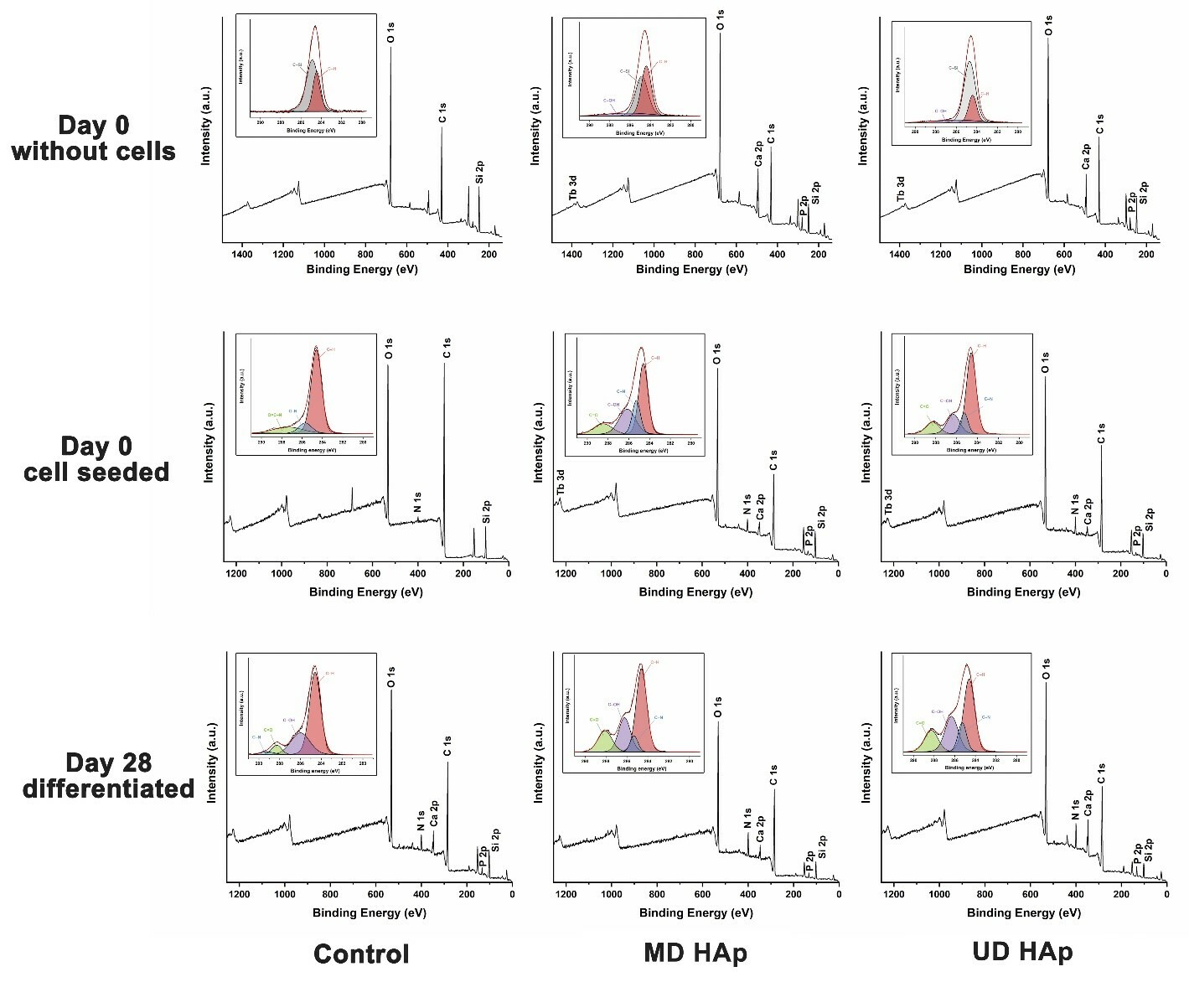


Figure S14. XPS analysis of chemical bonds of substrates with or without hBMSCs.

The survey spectrum and the high-resolution spectrum of C are investigated. Before cell seeding, C, O, and Si are the major elements in the control group, which corresponds to PDMS. In the high-resolution spectra, C–Si (284.8 eV) and C–H (284.6 eV) bonds are identified. In comparison, Ca, P, and Tb elements and C–OH (286.2 eV) bonds are found in the MD and UD HAp NR groups. These results are attributed to the Cit/Tb–HAp coating and the good binding to PDMS, despite the coating methods. Significantly, cell seeding brings proteins and results in the appearance of N elements in the samples. The high-resolution spectra show C=O (288.3 eV), O=C–N (287.4 eV), and C-N (285.3 eV and 289.2 eV), which suggests a strong attachment of hBMSCs to the coatings. After differentiation, because most hBMSCs secreted and interacted with calcium deposits, a stronger C–OH (286.2 eV) peak was observed in all groups. The increase in cell number also led to a more significant N peak in all day 28 spectra.

**
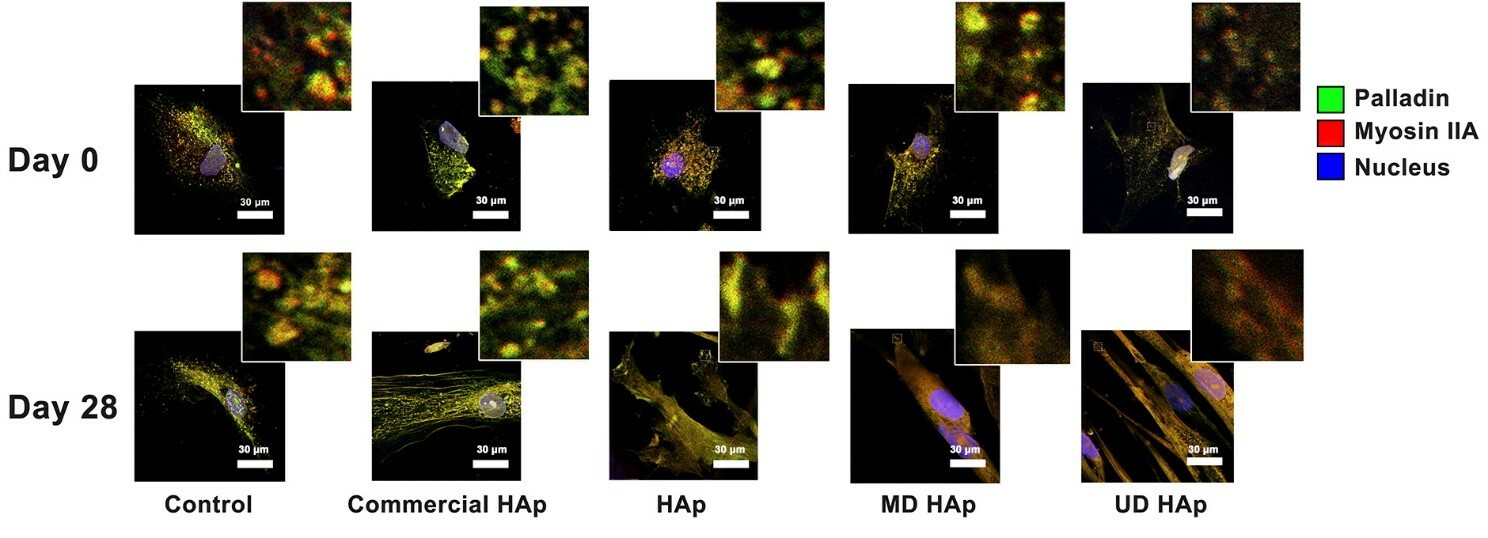
Figure S15. Confocal microscope visualization of hBMSC FA distribution on different surfaces on days 0 and 28 of osteogenic differentiation**. Green: palladin; red: myosin IIA; blue: nucleus. Captured using a 63× oil objective. Scale bar = 30 μm.


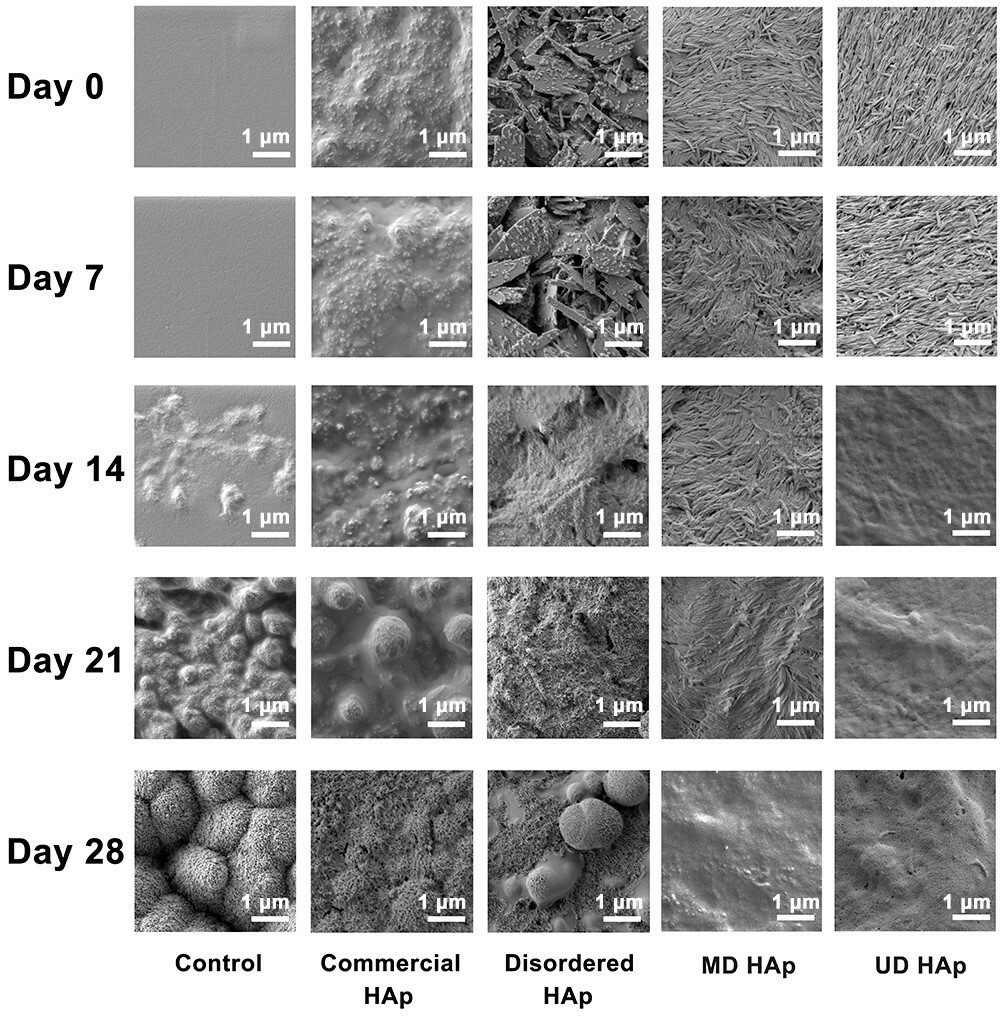


**Figure S16. FE-SEM imaging of the HAp substrate and calcium deposit topography secreted by hBMSCs on days 0, 7, 14, 21, and 28**. Scale bar = 1 μm.


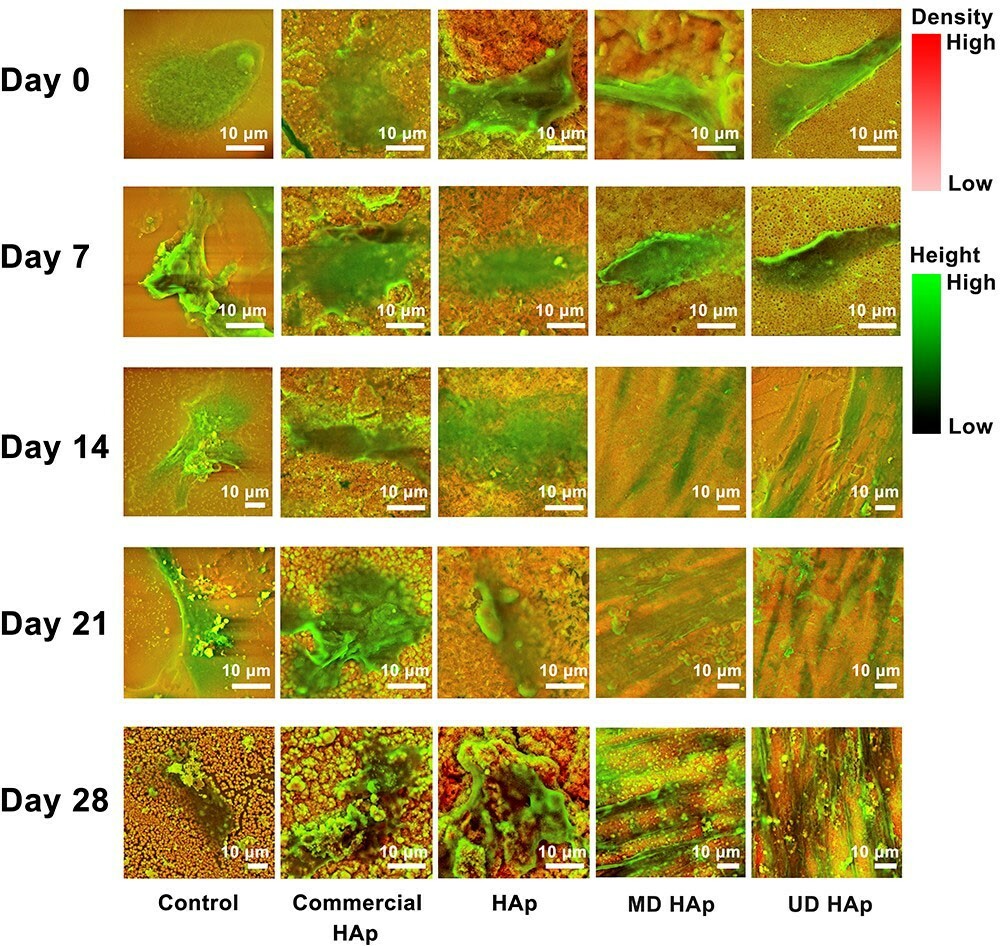


Figure S17. Density-Dependent Color Scanning Electron Micrograph SEM (DDC-SEM) imaging for hBMSCs morphology. hBMSCs morphology on control surface (PDMS) and different HAp coatings on day 0, 7, 14,21, and 28. Red channel: AsB detector in high vacuum (HV) mode, giving density information. Bright red indicates high density components; green channel: VPSE G4 detector in variable pressure (VP) mode of 50 Pa, giving height information. Bright green represents high topography. Scale bar = 10 μm.


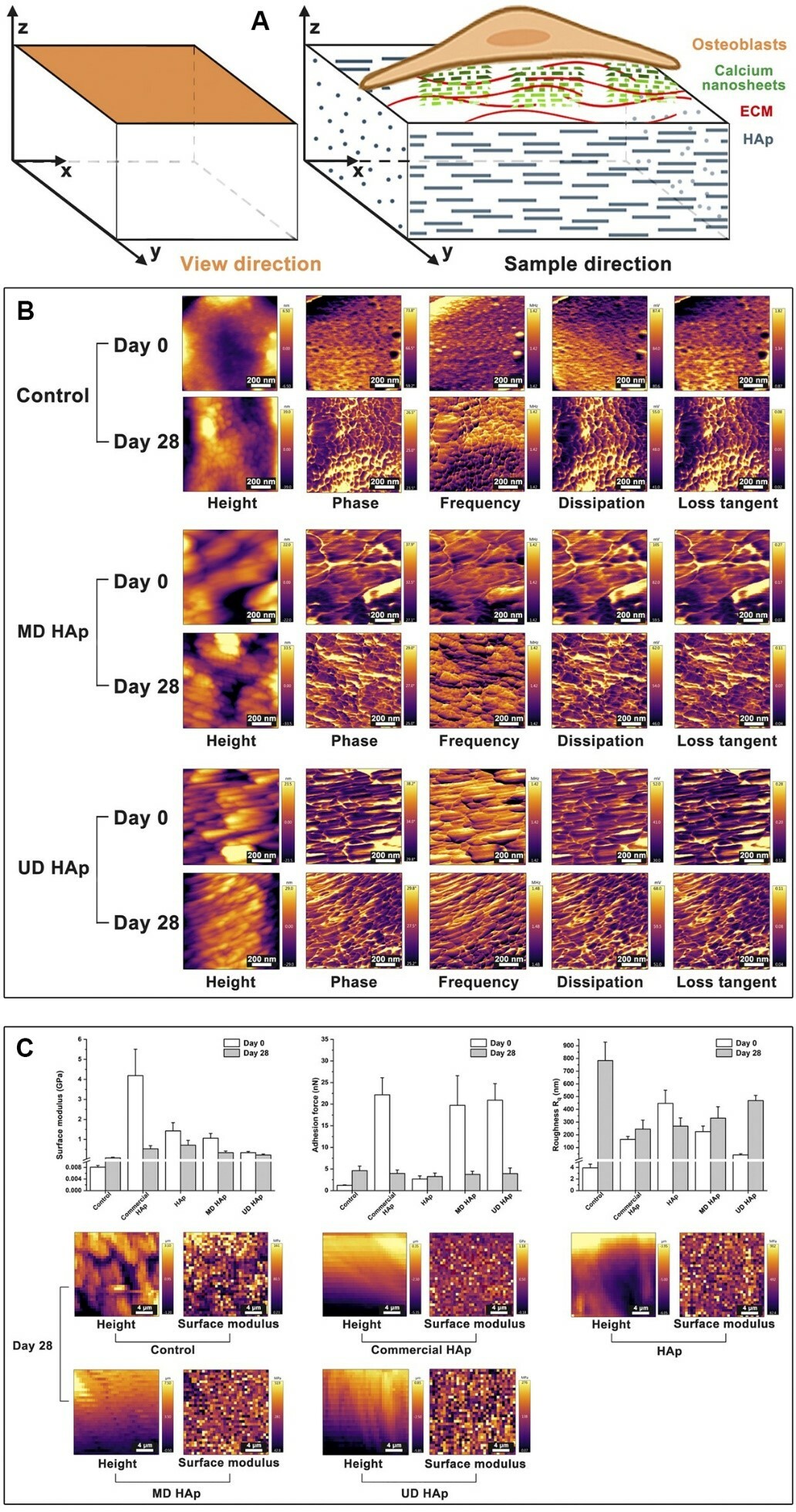


Figure S18. AFM surface characteristics of HAp samples.

**(A)** Schematic of sample and view direction. In these tests, the samples were measured at their surface. (**B)** AM-FM mode of AFM for surface characterization. Control, MD, and UD HAp groups on days 0 and 28 of osteogenic differentiation. Both the MD and UD HAp groups exhibited aligned topography in height on day 28. Differences in Phase (static measurement) are utilized to calculate surface modulus, adhesion force, and roughness in (**C)**. It is shown that the surface modulus and adhesion force of calcium deposits on day 28 in both MD and UD HAp groups are lower than their HAp substrates on day 0. This may be a result of the ingrowth of organic ECM components (or mineralized ECM). The change in the median phase value further proves that the HAp substrates have been covered with a new layer of secretion. The dissipation (dynamic measurement) reflects energy loss during the contact between the cantilever and material surface. A higher dissipation will be on a soft or viscous surface. On day 28, the median dissipation in the control group dropped to 48.0 mV, and the MD and UD HAp NR groups changed to 54.0 mV **–** 59.5 mV. The loss tangent (dynamic measurement) reflects the ratio of the loss modulus to the storage modulus (tan δ = E’’/E’), which can be used to characterize viscoelastic materials. All groups displayed lower median loss tangent (ranging from 0.05 to 0.08) on day 28, compared to higher values on day 0. In both MD and UD groups, broad and non-uniform areas with high loss tangent can be found at the boundaries of HAp NRs due to the grain-grain gaps, indicating a loose packing of the HAp NRs. To be noted, the lower right area of MD HAp group on day 0 is excluded for analysis as its high loss tangent value may be an artificial effect of signal loss due to the non-uniform height of the dip-coated sample. After 28 days, fine, uniform, and aligned boundaries with high loss tangent appear in the UD HAp group, which can be interpreted as the in-grown ECM components tightly packing up the calcium deposit nanocrystals. The grain-grain distance is about 36.8 nm, strongly correlates the lamellar distance of 36.4 nm measured by STEM (**Figure** S17C3). Scanning area = 1 μm × 1 μm. Scale bar = 200 nm. (**C)** AFM contact mode for substrate mechanical property characterization. Control, MD, and UD HAp groups on days 0 and 28 of osteogenic differentiation. The commercial HAp had the highest surface modulus at approximately 4 GPa, followed by HAp and MD HAp NRs. On day 28, all groups reached a lower and similar surface modulus lower than 1 GPa. In terms of adhesion force, the commercial, MD, and UD HAp NRs had a higher adhesion force on day 0 but reached a lower but close level of approximately 4 nN. All groups witnessed an increase in roughness except for the HAp group. Scanning area = 20 μm × 20 μm. Scale bar = 4 μm.


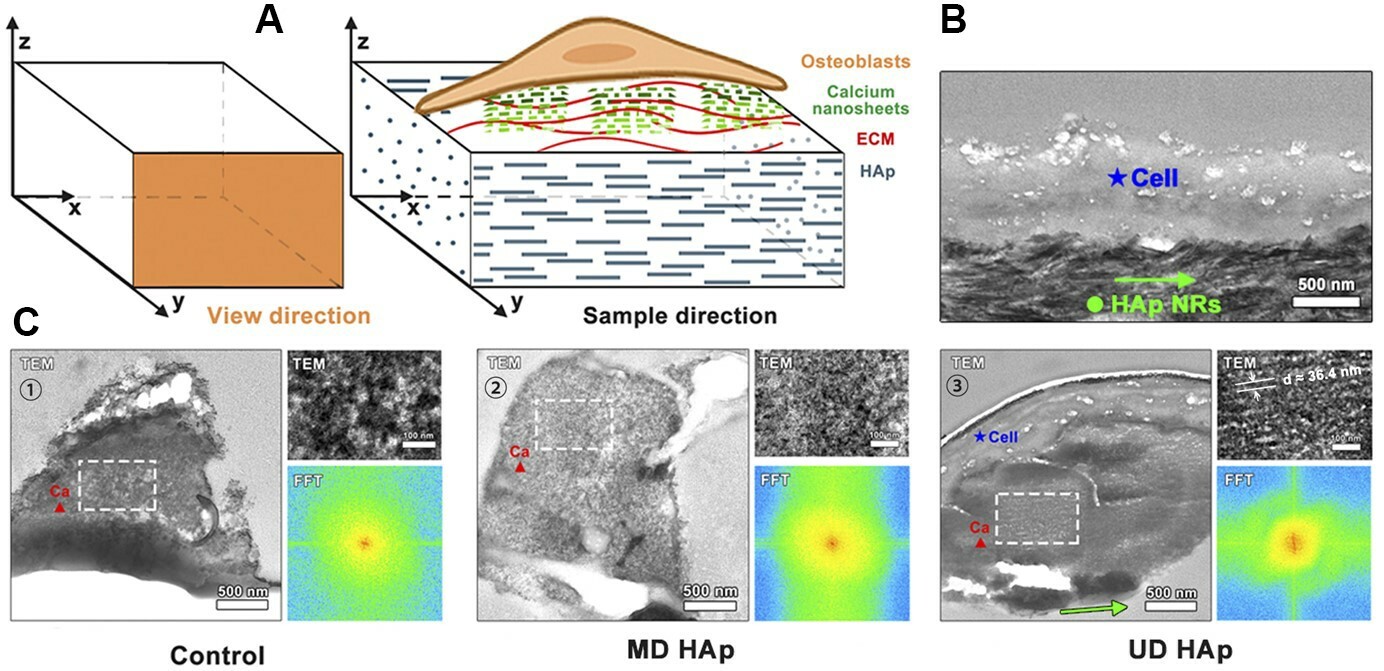


Figure S19. Visualization of calcium deposits using STEM.

**(A)** Schematic of sample and view direction. In these STEM scanning, the samples were measured at their cross-section. (**B)** The section shows horizontally aligned HAp NRs, proving samples were sectioned along the direction of HAp NRs. **(C) ①–③** Section views give direct evidence of the inner structure of calcium deposits from different groups. On day 28, both control and MD HAp groups show irregular patterns, while the mineral size in the MD HAp group is finer. Interestingly, the UD HAp group forms calcium deposit with parallel sheet-like structure comprised of nanocrystals. The distance between sheets is approximately 36.4 nm, corresponding to near q = 0.18 nm^-1^ in the SAXS 1D plot. The STEM images were then converted into FFT for anisotropy analysis. In the UD HAp group, the anisotropic arrangement of nanosheets is confirmed by the polarizing 2D pattern of FFT. Blue star: cell; red triangle: calcium deposit; green circle: HAp NRs; green arrow: direction of HAp NRs. Scale bar = 500 nm.

**
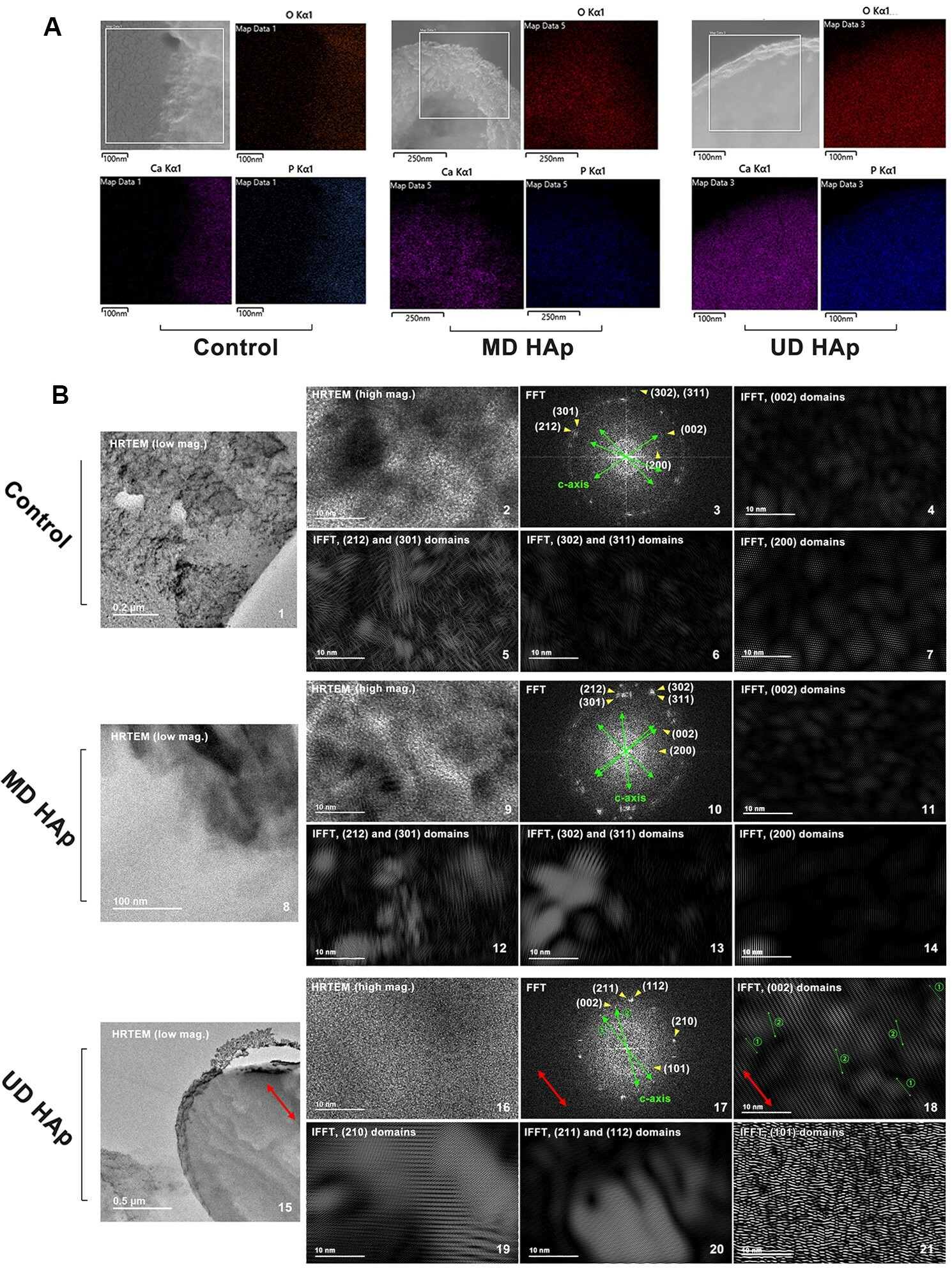
**

Figure S20. EDX and HRTEM imaging of calcium deposits on day 28.

**(A)** The Ca, P, and O element mapping of deposits in different groups using EDX. **B1, B8, and B15,** HRTEM low and **B2, B9,** and **B16,** high magnification views of calcium deposits of control, MD HAp, and UD HAp groups, respectively. Red arrows indicate the orientation of UD HAp NRs. **B3, B10, and B17,** resulting FFT images of the entire high magnification views. Red arrows indicate the orientation of UD HAp NRs while green arrows represent the identified orientation of (002) plane (c-axis). Specially, green arrows **B17①** and **B17②** label two orientations of (002) plane in the UD HAp group. Yellow arrow heads indicate other identified lattice planes. **B4** **– B7, B11 – B14, and B18 – B21,** The IFFT images of the corresponding lattice plane, showing their domains located in **B2**, B**9**, or **B16**, respectively. Specially, green arrows **B18①** and **B18②** distinguish the two corresponding orientations, **B17①** and **B17②,** of (002) domains. Scale bar = 10 nm.


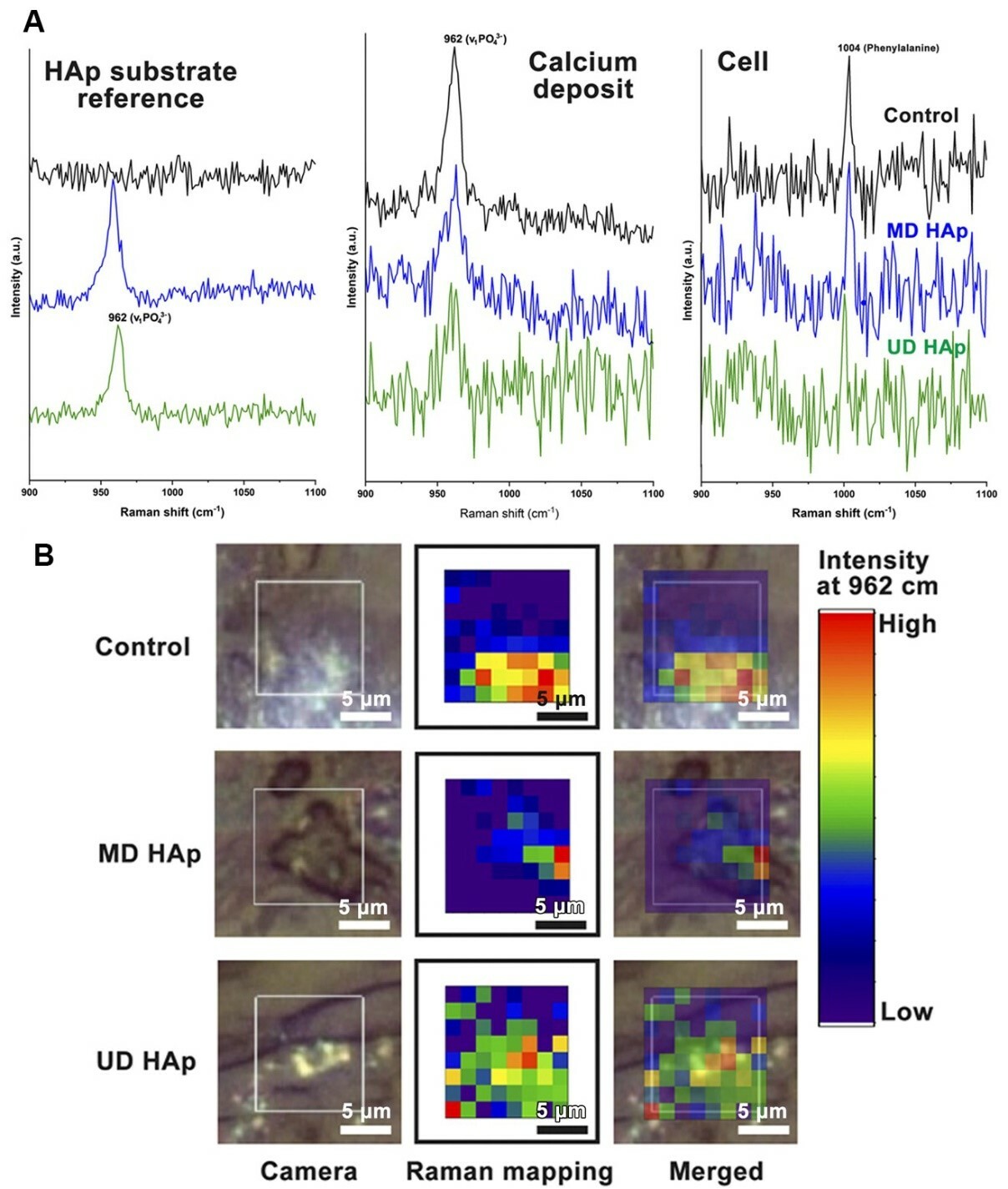


Figure S21. Raman spectra analysis of calcium deposits.

**(A)** Raman spectra of calcium deposit on different substrates. Single measurement was performed on different component to acquire characteristic peaks, followed by 2D mapping of ν_1_(PO_4_^3-^) at 962 cm^-1^. The single point measurement clearly shows that the calcium deposits have symmetrical stretching vibration of phosphate near 962 cm^−1^, similar but much weaker (noisier) than the HAp substrate reference. The peak at around 1004 cm−1 represents the phenylalanine from cells. **(B)** The Raman 2D mappings at 962 cm^−1^ show that the calcium deposits have distinguishable boundaries and are denser at their center. Scale bar = 5 μm.


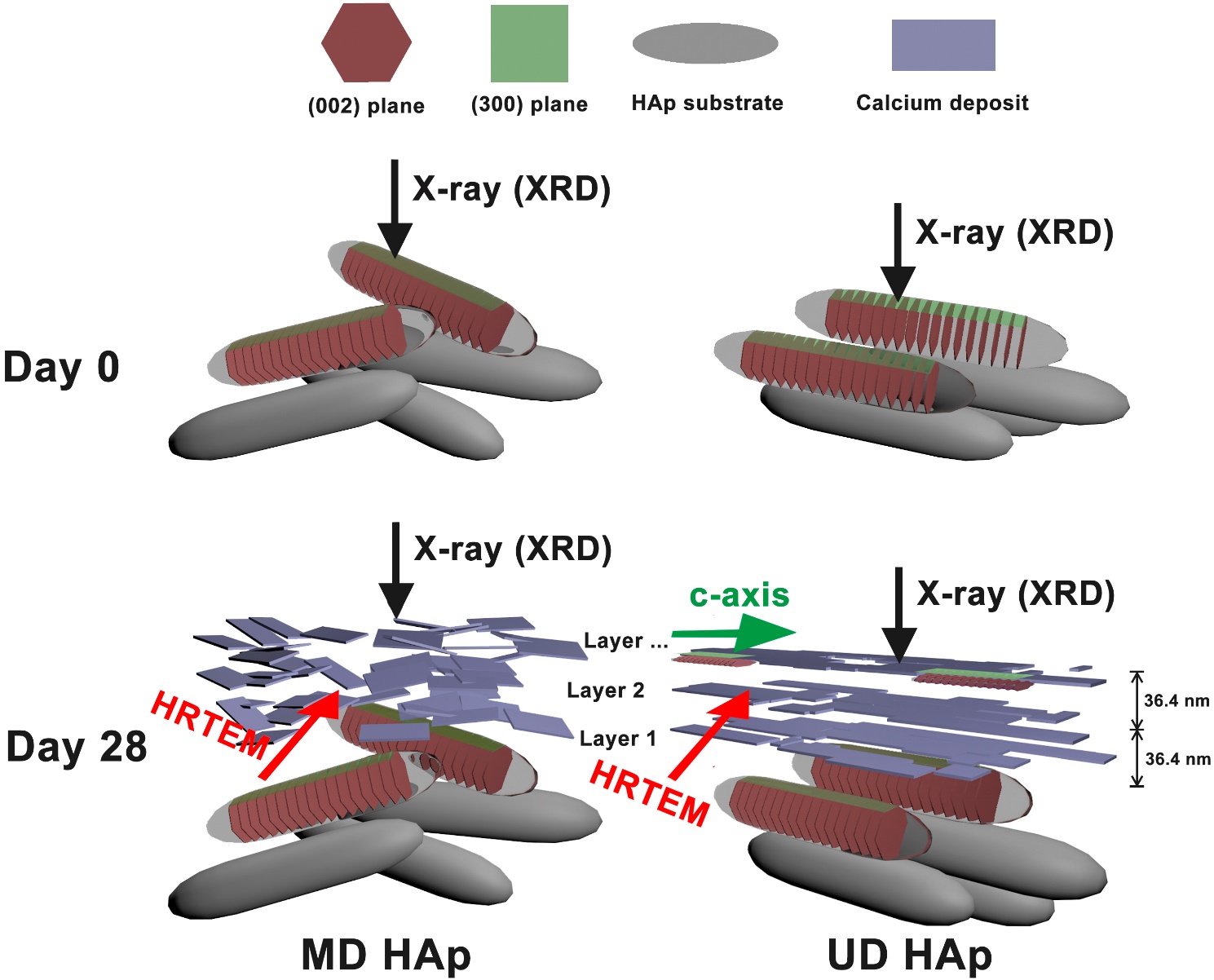


Figure S22. Schematic of different mineralization process on MD or UD HAp NRs.

On day 0, MD HAp NRs show alignment after dip-coating but inevitably form aggregations which lead to non-uniform orientation of (002) plane. UD HAp NRs are uniformly aligned along (002) direction via spin coating. The (200)/(300) are the planes perpendicular to (002). On day 28, apatite polycrystal domains with random (200)/(300) and (002) orientation are generated on the MD HAp NRs. In comparison, apatite nanosheets on UD HAp NRs have preferred growth direction on (002) plane (c-axis), following that of UD HAp NRs. The nanosheets then assemble into a secondary lamellar structure, about 36.4 nm between layers. Black arrow: direction of XRD incident beam, perpendicular to the sample surface; Red arrow: direction of HRTEM beam, horizontal to the sample surface; Green arrows: direction of c-axis of the calcium deposits on UD HAp NRs.


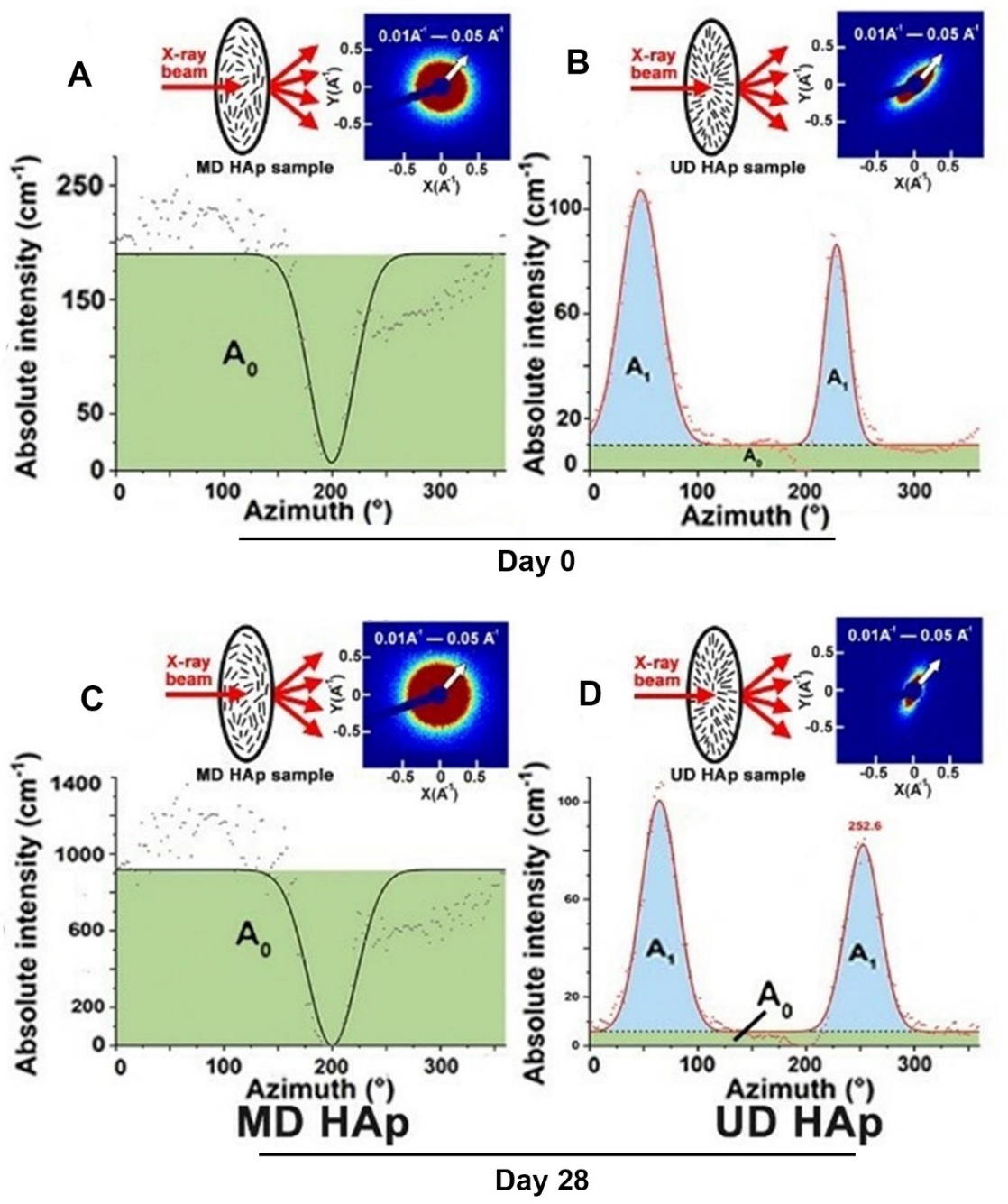


Figure S23. SAXS 2D patterns and analysis. Converted SAXS 2D patterns and analysis of calcium deposits in the MD and UD HAp groups (on days 0 and 28) of osteogenic differentiation. (A) – (D) SAXS 2D patterns and corresponding 1D azimuth plots.


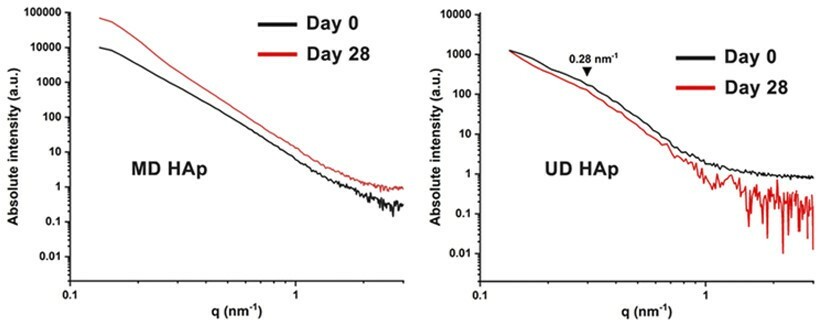


Figure S24. The 1D SAXS plots of MD and UD HAp groups on day 0 (black curves) and 28 (red curves). Converted from the 2D SAXS pattern in Figure 4E1 – 4E4 by integration of the scattering vector (q) ranging from 0.01 Å^-1^ to 0.05 Å^-1^. In UD HAp, it is noted that there is potential inaccuracy in determining the peak position due to the relative insignificance of the broad peak.


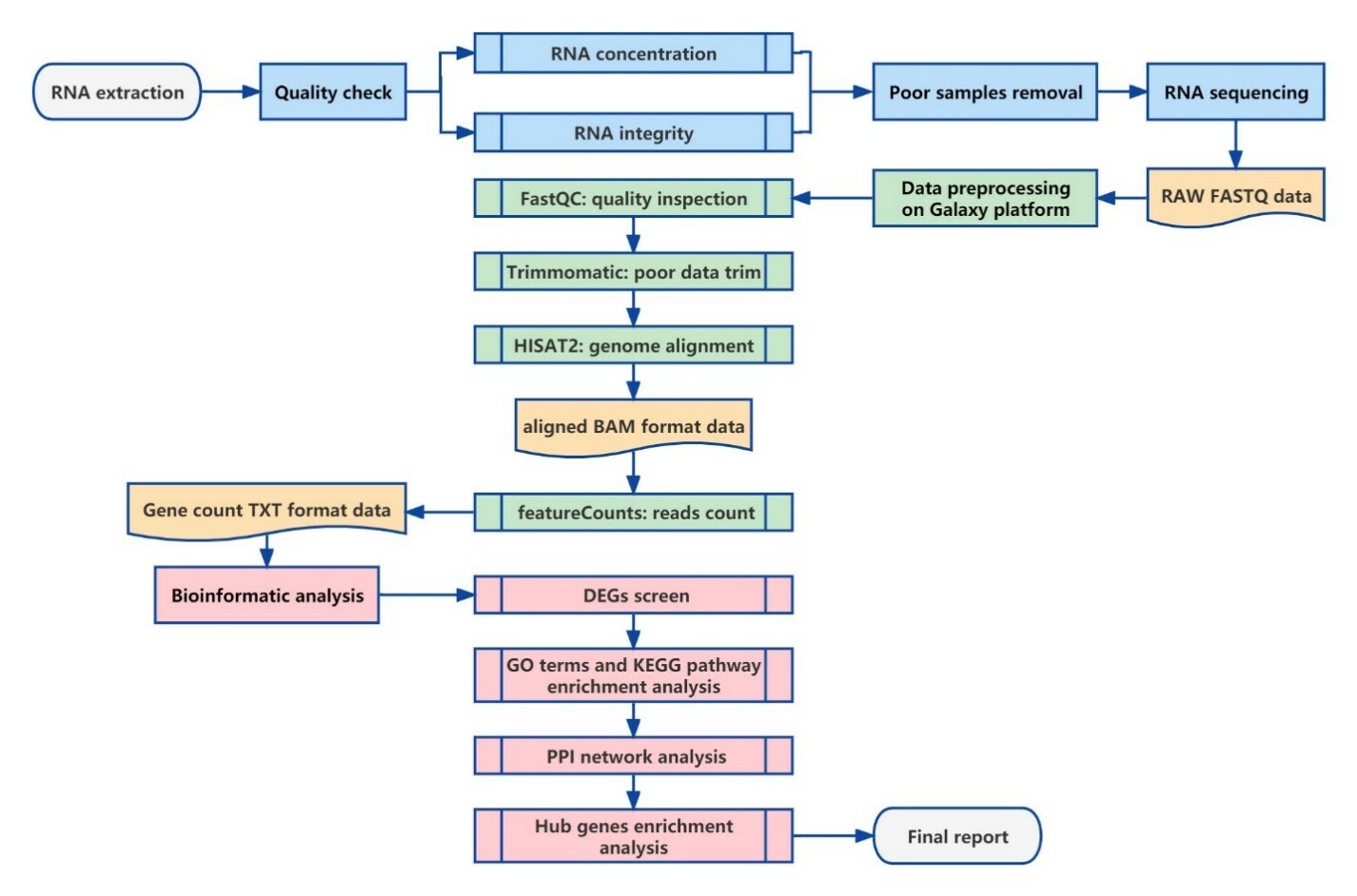


Figure S25. Flowchart of RNA-Seq.


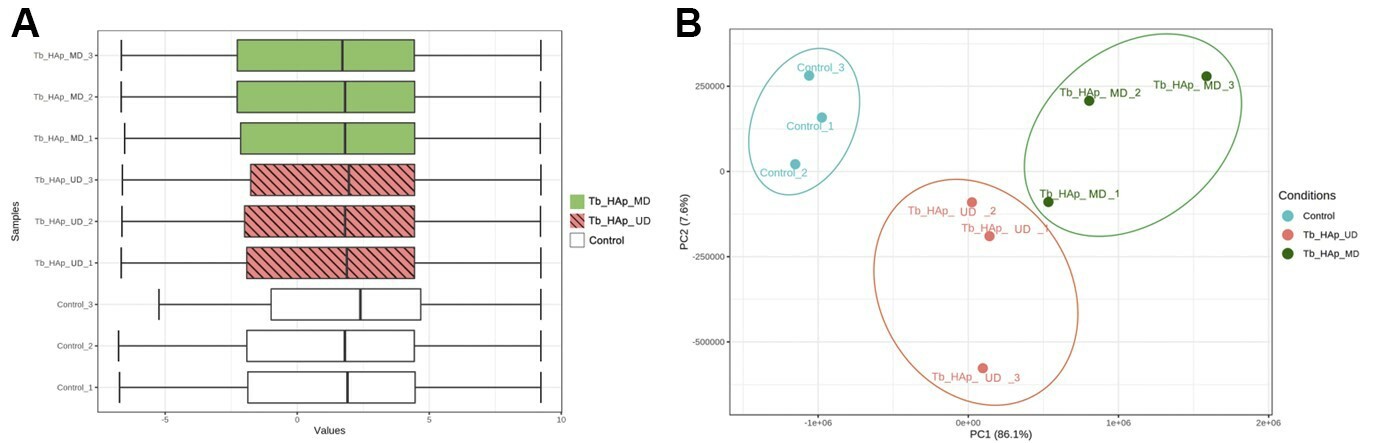
**Figure S26. Normalization and principal component analysis of gene datasets.**

**(A)** Boxplot of quantile normalized data. Vertical black lines in the boxes represent medians. (**B)** In the principal component analysis (PCA), one dot is a sample. The distance between samples represents similarity. Each condition *n* = 3.


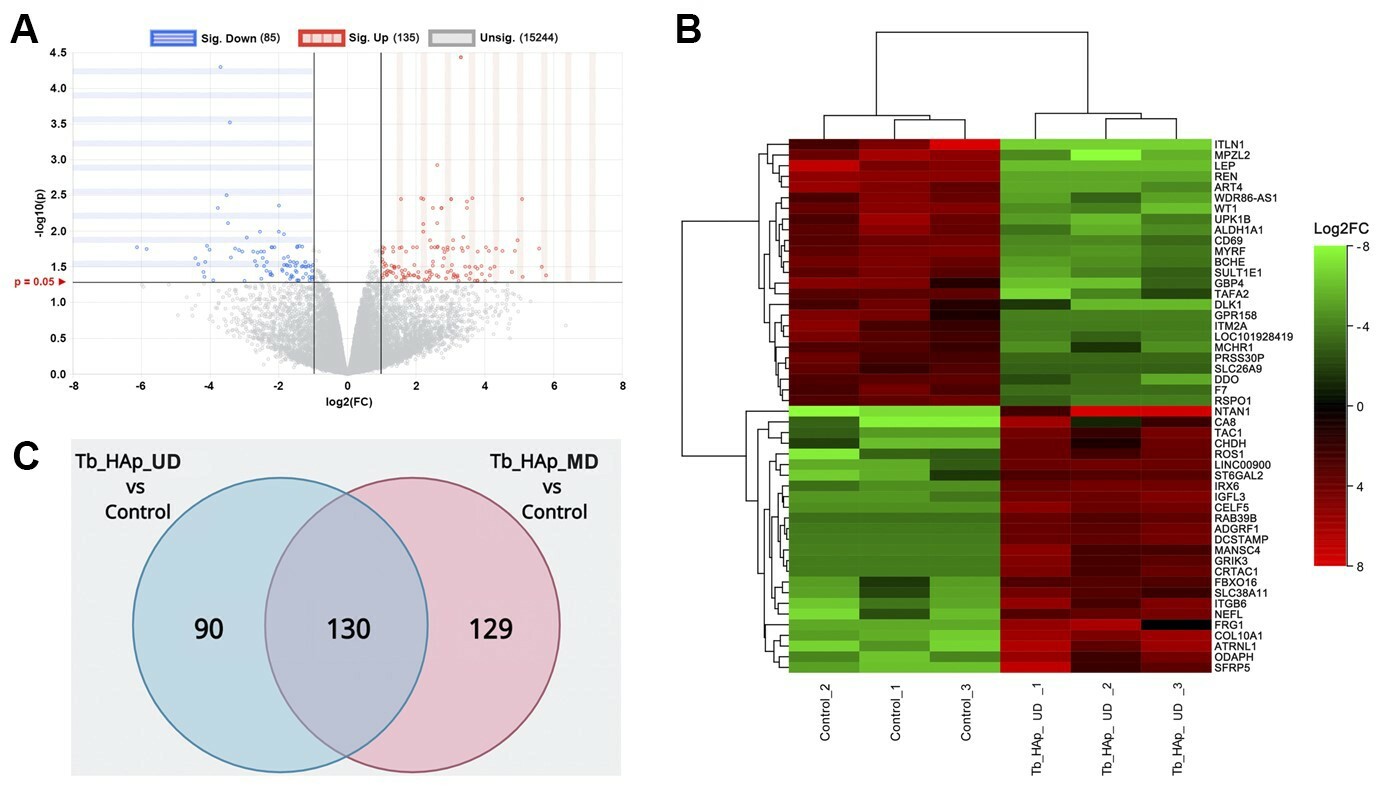


Figure S27. Visualization of differentially expressed genes.

**(A)** Volcano plot of all analyzed genes of the UD HAp group. Each dot represents a gene. Vertical blue or horizontal red lines divide areas of down- or upregulated genes. The X-axis is the log2-base fold change, and the Y-axis is the −log10-base adjusted p value. (**B)** The clustered heatmap demonstrates the top 50 DEGs with the greatest absolute fold change of the UD HAp group and the control group. Each row is a gene, and each column is a sample. Red indicates upregulation, while green represents downregulation. (**C)** Venn diagram of DEGs of the MD (259 DEGs) and UD HAp (220 DEGs) groups compared to the control group. A total of 130 DEGs overlapped between the MD and UD HAp groups.


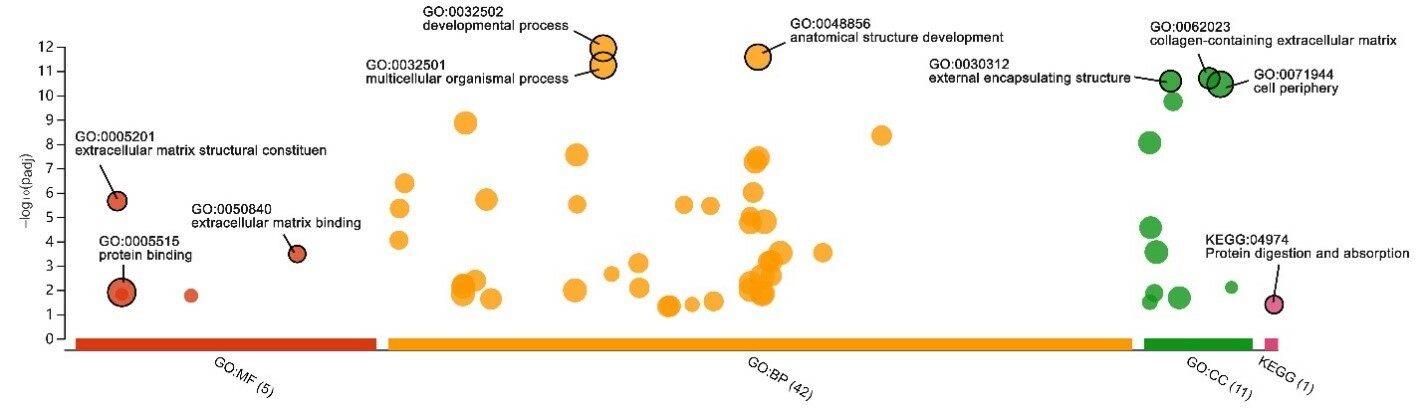


Figure S28. GO enrichment and KEGG pathway enrichment analysis of 220 DEGs in the UD HAp group. On the y-axis, higher values correspond to more significantly enriched terms. The horizontal distance indicates similarity between terms. Terms from the same GO subtree are located closer to each other. Bubble size stands for term size. Different colors distinguish terms from different GO categories.


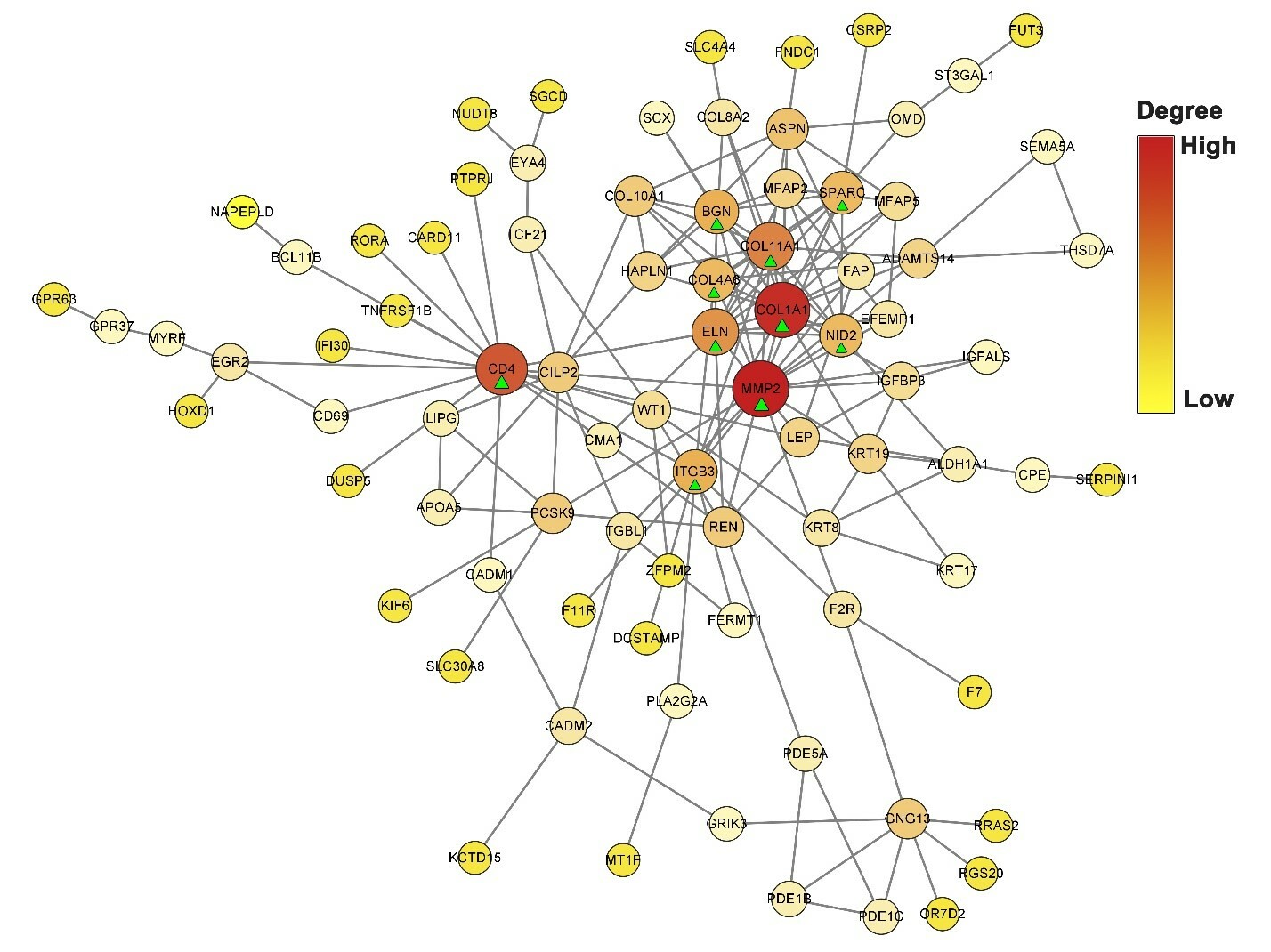


Figure S29. PPI network of all DEGs of the UD HAp group. Red indicates a high connection degree, while yellow represents a low connection degree. Nodes with higher degrees also have larger sizes. Nodes with bright green triangles represent hub genes analyzed by cytoHubba.


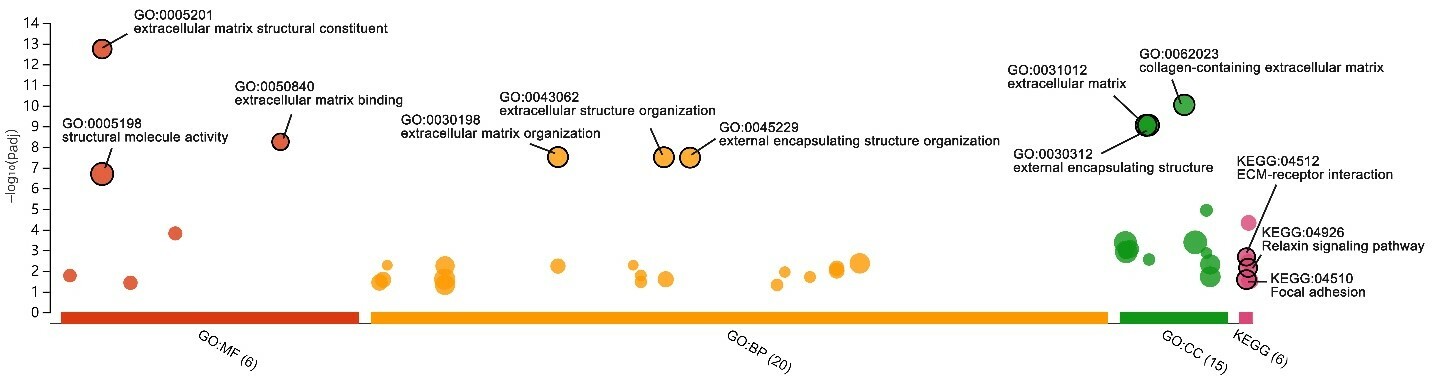


Figure S30. GO enrichment and KEGG pathway enrichment analysis of 10 hub genes of the UD HAp group. On the y-axis, higher values correspond to more significantly enriched terms. The horizontal distance indicates similarity between terms. Terms from the same GO subtree are located closer to each other. Bubble size stands for term size. Different colors distinguish terms from different GO categories.


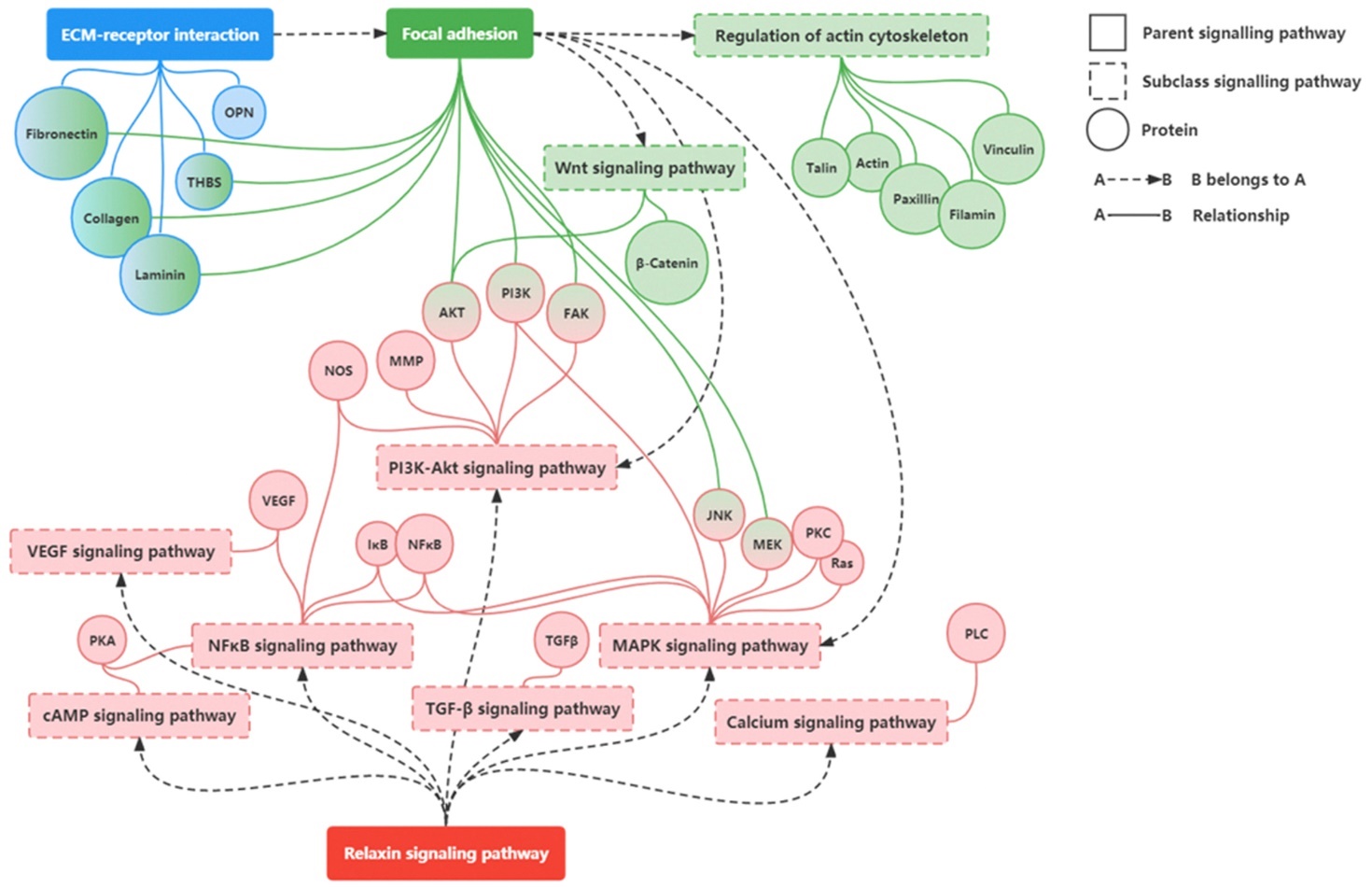


**Figure S31. The crosstalk network of enriched signaling pathways and related major proteins in the UD HAp group.**


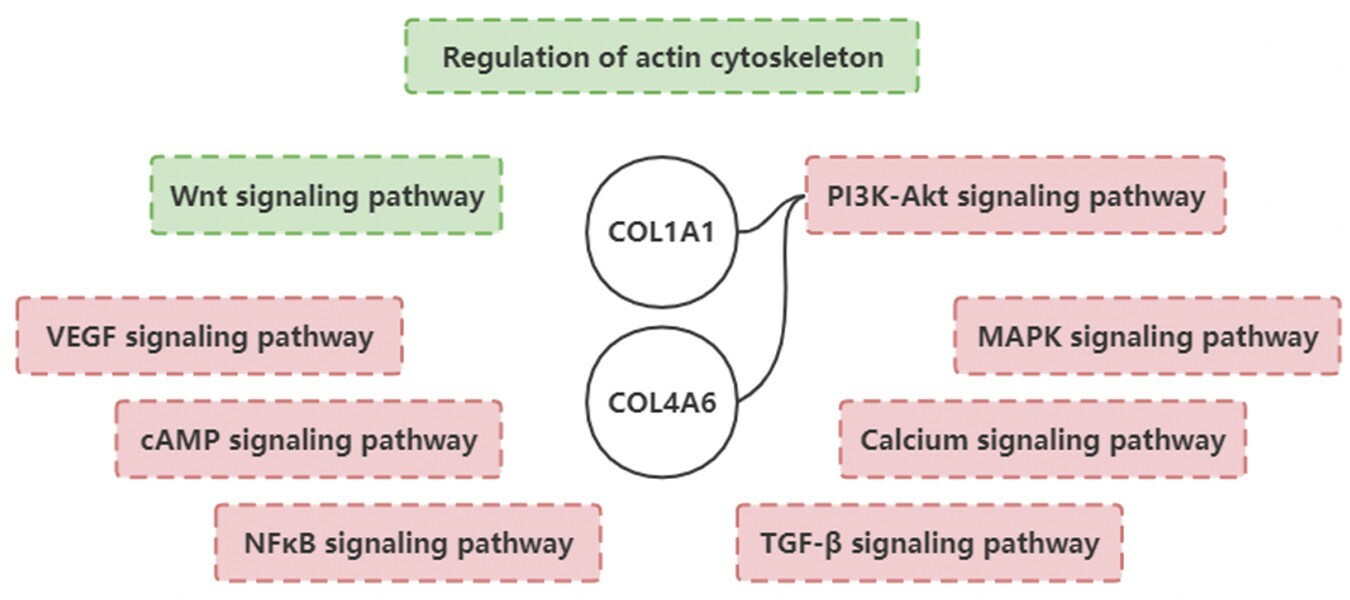


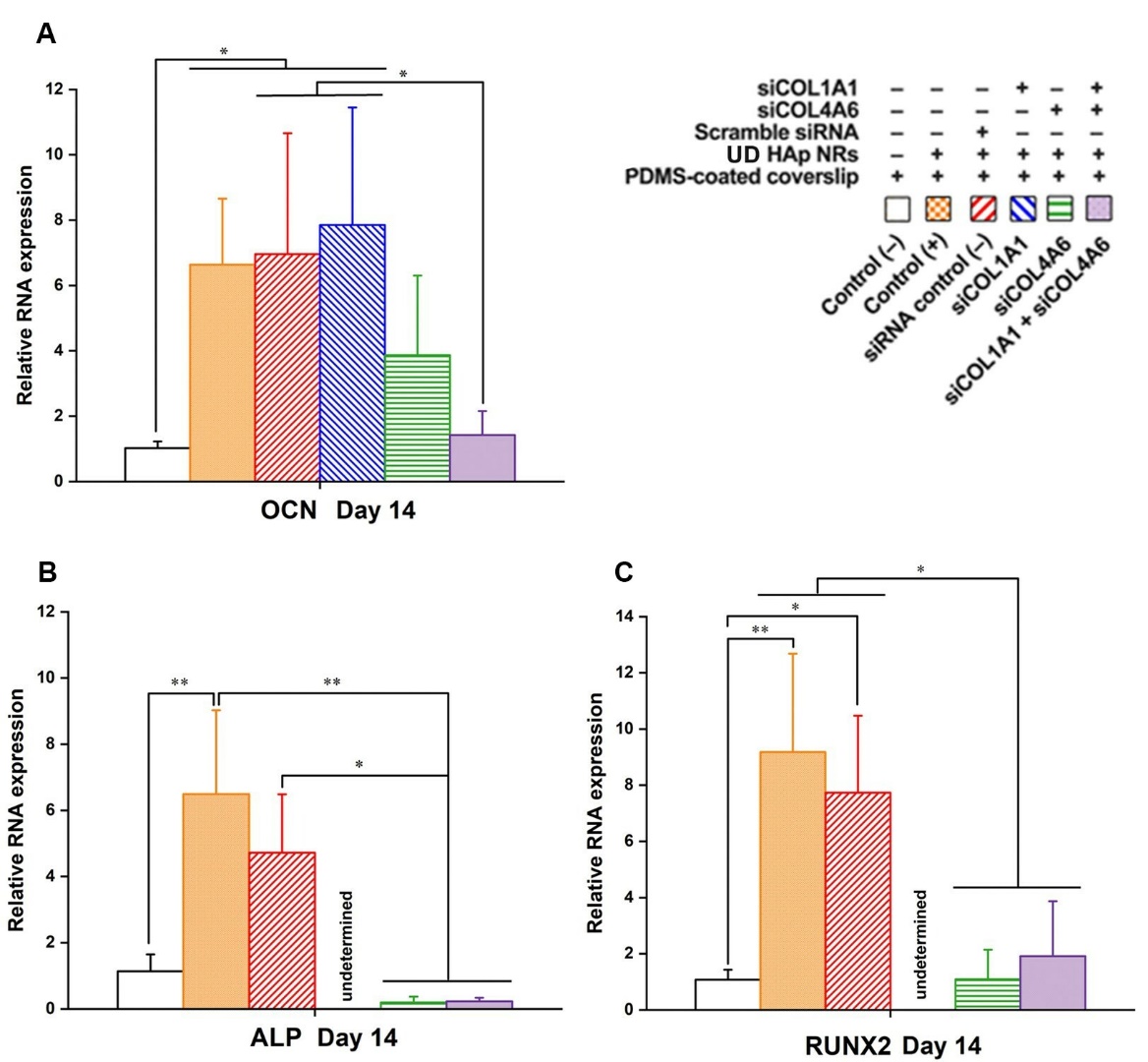
Figure S32. COL1A1 and COL4A6 were found only to be involved in the PI3K-Akt signaling pathway.

**Figure S33. PCR results of osteogenic genes after RNAi.** The expression of the osteogenic genes A, OCN, B, ALP, and C, RUNX2 was evaluated by PCR. The control (+) and siRNA control (–) show significantly higher expression of all three genes than the control (–). After RNAi of COL1A1 and/or COL4A6, the expression of these genes is significantly hampered. Each condition *n* = 3. Statistical analysis: one-way ANOVA. *: P <0.05; **: P <0.01; ***: P <0.001.


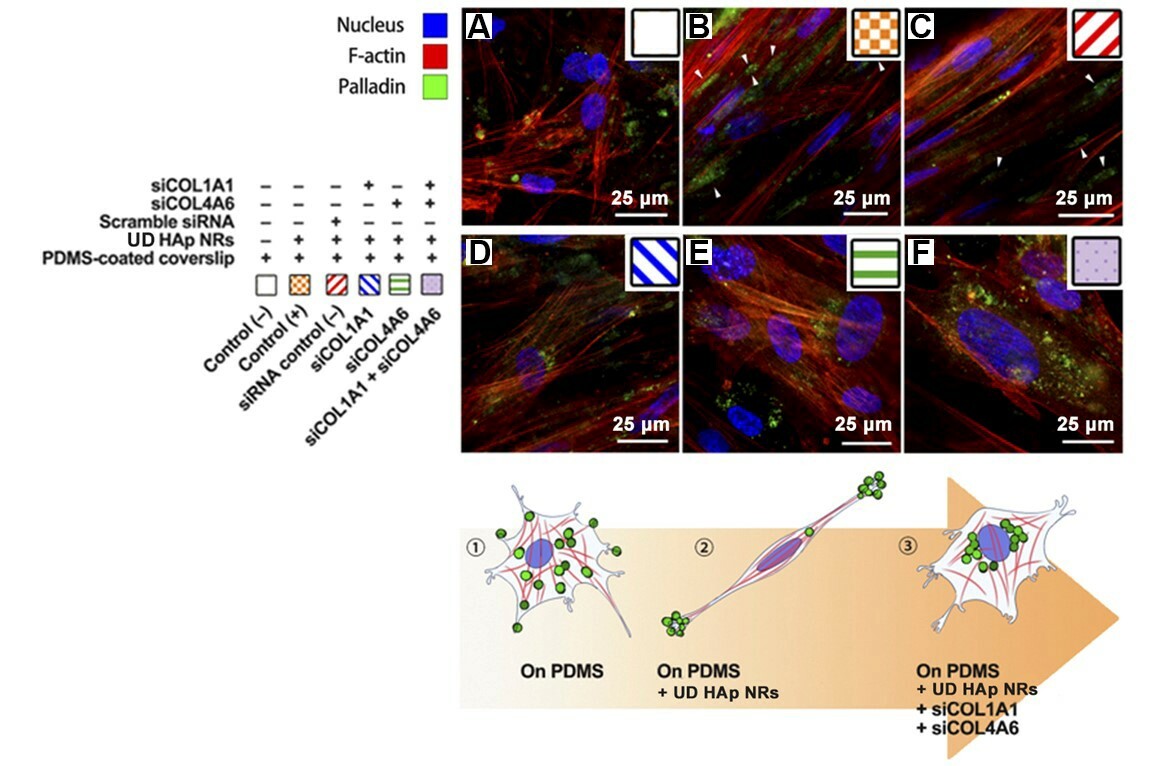


Figure S34. Visualization of focal adhesions after RNAi using confocal microscopy and schematic of the focal adhesion distribution pattern.

**(A – F)** Focal adhesions (FAs) can be visualized using a confocal microscope. Their distribution shows typical patterns. In the control (–), FAs are randomly distributed within the cell body. On UD HAp NRs, hBMSCs display an elongated morphology, and FAs gather into clusters at two ends of the cell body. After RNAi, cells lose elongated morphology, and FAs shrink to the region surrounding the nucleus. Scale bar = 25 μm. ①-③**,** Schematic of three typical types of FA distribution patterns.


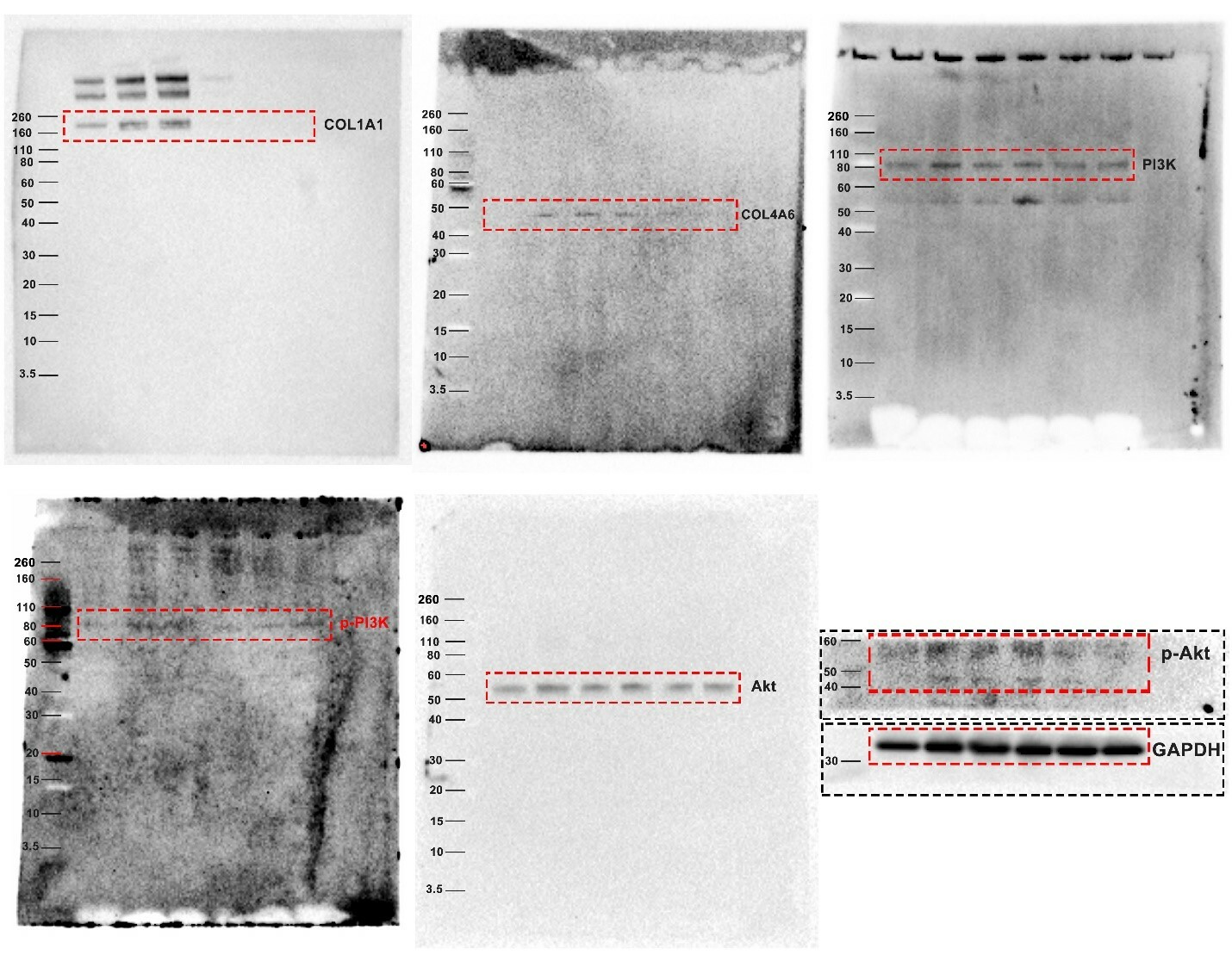


Figure S35. Uncropped western blot membranes. Black dashed lines indicate where the membranes were cut in the case of incubating multiple antibodies simultaneously. Red dashed lines indicate where membranes were selected to display.


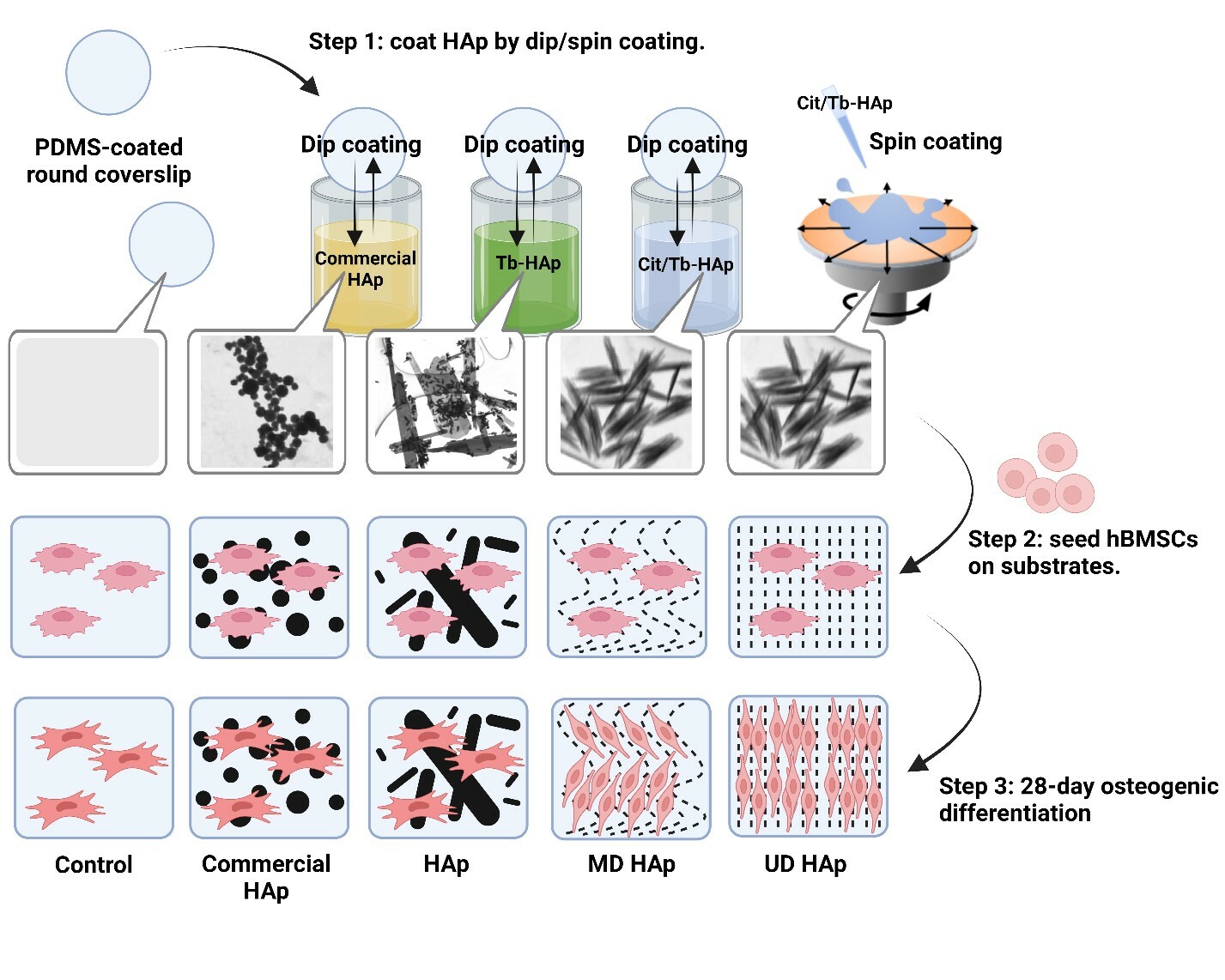


Figure S36. Schematic of the grouping and treatment methodology for the osteogenic differentiation experiment.

Five groups were set up: control group, commercial HAp, HAp, MD HAp NRs, and UD HAp NRs, prepared as previously described. Then, a density of 2.5 × 10^4^ hBMSCs per well was seeded on top of the substrates, followed by 28 days of osteogenic differentiation culture.

Table S1. Crystalline measurements calculated from XRD. Note: FWHM: full width half maximum; CI: crystallinity index; R: texture index.

|  | **2θ_(002)_ (°)** | **FWHM_(002)_ (Δ°)** | **2θ_(300)_ (°)** | **FWHM_(300)_ (Δ°)** | **(CI)_XRD_ by intensity** | **(CI)_XRD_ by area (%)** | **R_(200)_** | **R_(300)_** | **R_(002)_** |
| --- | --- | --- | --- | --- | --- | --- | --- | --- | --- |
| **Commercial HAp** | 25.84 | 0.46 | 33.04 | 0.48 | 0.64 | 96.3 | 1.08 | 1.03 | 0.92 |
| **HAp** | 25.88 | 0.12 | 32.87 | 0.33 | 1.25 | 92.7 | 1.12 | 1.03 | 0.44 |
| **Tb-HAp** | 25.88 | 0.22 | 32.90 | 0.34 | 0.91 | 88.5 | 1.04 | 1.02 | 0.75 |
| **Cit-HAp** | 25.88 | 0.26 | 32.71 | 1.03 | 0.34 | 92.4 | 1.08 | 1.24 | 0.48 |
| **Cit/Tb-HAp** | 25.90 | 0.18 | 32.62 | 1.13 | 0.56 | 90.9 | 1.17 | 1.27 | 0.32 |

**Table S2. Particle size of HAp samples calculated from STEM images (n = 300).**

| **Sample** | **Length (nm)** | **Diameter (nm)** | **Aspect ratio, L/D** |
| --- | --- | --- | --- |
| Commercial HAp | 40 ± 10 | 40 ± 10 | 1 ± 0 |
| HAp | 992 ± 699 | 136 ± 70 | 9 ± 6 |
| Tb-HAp | 1023 ± 413 | 184 ± 81 | 7 ± 4 |
| Cit-HAp | 267 ± 63 | 27 ± 3 | 10 ± 2 |
| Cit/Tb-HAp | 235 ± 38 | 24 ± 4 | 10 ± 3 |

Table S3. Element ratio of HAp samples analyzed by EDX.

| **Sample** | **Ca/P (mol%)** | **Tb/(Ca + Tb) (mol%)** |
| --- | --- | --- |
| Theoretical value | 1.67 | 4 |
| Commercial HAp | 1.68 ± 0.03 | - |
| HAp | 1.28 ± 0.13 | - |
| Tb-HAp | 1.25 ± 0.02 | 5.24 ± 0.23 |
| Cit-HAp | 1.51 ± 0.17 | - |
| Cit/Tb-HAp | 1.36 ± 0.04 | 4.77 ± 0.03 |

**Table S4.** Comparison of particle size and interparticle distance (*d_//_* and *d_⊥_*) corresponding to SAXS *q* values (*q_//_* and *q_⊥_*) measured in different HAp LCs reported.^[25-27]^

| **HAp Liquid Crystals** | | **This study** | **Hoshino et. al, Soft Matt, 2019**^[25]^ | **Tan et. al, Sci. Rep., 2019**^[26]^ | **Tan et. al.,**  **Acta Biomat, 2020**^[27]^ |
| --- | --- | --- | --- | --- | --- |
| **Synthesis method/Composition** | | Cit/Tb-HAp | PAA/HAp | Cit/HAp NRs | Cit/HAp NRs |
| **Length, nm** | | 325 ± 38 | 100 | 60.6 | 228.23 ± 99.22 |
| **Diameter, nm** | | 24 ± 4 | 20 | 11.9 | 4.31 ± 1.83 |
| **Aspect ratio, *L/D*** | | 13.54 | 5 | 5.3 | 72.75 |
| **Solvent** | | water | water | water | cyclohexane, |
| **I-N coexistence phase, Concentration** | | 14.8-27.2 wt% | 6.3 ~ 6.5 Vol% | 19.4-24.2% | 15.2 ~ 32.4 wt% |
| **LC phase, Critical concentration** | | > 32.4-35.1 wt% | >6.5 Vol% | 24.2%,  Note: 8.9 – 30.3% (g/cm^3^) depending on NaCl% (0 -0.5M) | >32.4 wt% |
| **Isotropic phase: Interparticle distance by SAXS** | | No peak | 49~37.6 nm | N/A | No peak (<17.5 wt%) |
| **LC: Interparticle distance by SAXS** | **in the long-axis** | No peak | *q_//_* ≈ 0.07 nm^-1^,  *d_//_* ≈ 89.71 nm | N/A | No peak |
|  | **in the short-axis** | No peak | *q_⊥_* ≈ 0.19 nm^-1^,  *d_⊥_* ≈ 33 nm | N/A | *q_⊥_* ≈ 0.8 nm^-1^,  *d_⊥_* ≈ 7.8 nm |

Table S5. Element ratio of calcium deposits in different groups analyzed by EDX.

| **Group** | **Ca/P (mol%)** | **Tb/(Ca + Tb) (mol%)** |
| --- | --- | --- |
| Control | 1.39 ± 0.04 | - |
| Commercial HAp | 1.55 ± 0.11 | - |
| Disordered HAp | 1.50 ± 0.08 | 9.66 ± 1.03 |
| MD HAp NRs | 1.58 ± 0.04 | 7.04 ± 1.05 |
| UD HAp NRs | 1.54 ± 0.04 | 7.59 ± 0.56 |

Table S6. Formulas of different HAp samples.

| **Reagent** | **Sample** | | | |
| --- | --- | --- | --- | --- |
|  | **HAp** | **Tb-HAp** | **Cit-HAp** | **Tb/Cit-HAp** |
| Ca(NO_3_)_3_ | 0.0045 mol | 0.00432 mol | 0.0045 mol | 0.00432 mol |
| (NH_4_)_2_HPO_4_ | 0.0027 mol | 0.0027 mol | 0.0027 mol | 0.0027 mol |
| C_6_H_5_Na_3_O_7_ | / | / | 0.006 mol | 0.006 mol |
| Tb(NO_3_)_3_ | / | 0.00018 mol | / | 0.00018 mol |

Table S7. Primer sequences used in the quantitative PCR analysis.

| Gene | Primer sequence |
| --- | --- |
| GAPDH | F: 5’-AACAGCGACACCCACTCC TC-3’ |
|  | R: 5’-CATACCAGGAAATGAGCTTGACAA-3’ |
| OCN | F: 5’-TGTGAGCTCAATCCGGACTGT-3’ |
|  | R: 5’-CCGATAGGCCTCCTGAAGC-3’ |
| ALP | F: 5’-ACCATTCCCACGTCTTCACATTT-3’ |
|  | R: 5’-AGACATTCTCTCGTTCACCGCC-3’ |
| RUNX2 | F: 5’-AGATGATGACACTGCCACCTC TG-3’ |
|  | R: 5’-GGGATGAAATGCTTGGGAACT-3’ |

Table S8. Standard one-step PCR cycling conditions.

| Step | Number of cycles | Temperature | Time |
| --- | --- | --- | --- |
| Reverse transcription | 1 | 48°C | 30 min |
| Polymerase activation | 1 | 95°C | 10 min |
| Amplification | 40 | 95°C | 15 sec |
|  |  | 60°C | 1 min |
| Melt curve | 1 | 95°C | 15 sec |
|  |  | 60°C | 15 sec |
|  |  | 95°C | 15 sec |

Table S9. Host information of hBMSCs.

| **Information** | **Description** |
| --- | --- |
| Product name | Human bone marrow-derived mesenchymal stem cells |
| Producer | ScienCell Research Laboratories (Carlsbad, CA, US) |
| Catalog number | 7500 |
| Lot number | 30579 |
| CA number | 0004483 |
| Host gender | Male |
| Host age | 20 wk. |
| Cell number | ≥ 5 × 10^5^ |
| Cell passage | Passage 0 |
| Viability | ≥ 70% |
| Proliferative potential | ≥ 15 PD |
| Mineral deposition after differentiation | Positive |
| Lipid staining after differentiation | Positive |
| HIV-1 DNA | Not detected |
| HBV DNA | Not detected |
| HCV DNA | Not detected |
| Mycoplasma DNA | Not detected |
| Bacteria/Fung | Negative |

**Table S10. Top 5 significantly enriched GO terms and KEGG pathways of DEGs of the UD HAp group.**

| **Category** | **GO ID** | **Description** | **Gene Count** | **Adjusted p value** |
| --- | --- | --- | --- | --- |
| BP | GO:0032502 | developmental process | 102 | 1.15×10^-12^ |
| BP | GO:0048856 | anatomical structure development | 96 | 2.69×10^-12^ |
| BP | GO:0032501 | multicellular organismal process | 110 | 5.85×10^-12^ |
| BP | GO:0007275 | multicellular organism development | 81 | 1.37×10^-9^ |
| BP | GO:0072359 | circulatory system development | 35 | 4.50×10^-9^ |
| CC | GO:0062023 | collagen-containing extracellular matrix | 24 | 1.97×10^-11^ |
| CC | GO:0030312 | external encapsulating structure | 27 | 2.61×10^-11^ |
| CC | GO:0071944 | cell periphery | 97 | 3.48×10^-11^ |
| CC | GO:0031012 | extracellular matrix | 26 | 1.79×10^-10^ |
| CC | GO:0005576 | extracellular region | 73 | 8.84×10^-9^ |
| ,MF | GO:0005201 | extracellular matrix structural constituent | 13 | 2.22×10^-6^ |
| ,MF | GO:0050840 | extracellular matrix binding | 7 | 3.31×10^-4^ |
| ,MF | GO:0005515 | protein binding | 150 | 1.26×10^-2^ |
| ,MF | GO:0005520 | insulin-like growth factor binding | 4 | 1.53×10^-2^ |
| ,MF | GO:0030020 | extracellular matrix structural constituent conferring tensile strength | 5 | 1.72×10^-2^ |
| KEGG | KEGG:04974 | Protein digestion and absorption | 77 | 4.00×10^-2^ |

**Table S11. The top 10 hub genes.**

| **Rank** | **Gene symbol** | **Degree** |
| --- | --- | --- |
| 1 | MMP2 | 20.0 |
| 2 | COL1A1 | 19.0 |
| 3 | CD4 | 16.0 |
| 4 | COL11A1 | 13.0 |
| 5 | ELN | 12.0 |
| 6 | ITGB3 | 10.0 |
| 7 | BGN | 10.0 |
| 8 | SPARC | 9.0 |
| 9 | NID2 | 9.0 |
| 10 | COL4A6 | 9.0 |

**Table S12. Top 5 significantly enriched GO terms and KEGG pathways of hub genes of the UD HAp group.**

| **Category** | **GO ID** | **Description** | **Gene Count** | **Adjusted p value** |
| --- | --- | --- | --- | --- |
| BP | GO:0030198 | extracellular matrix organization | 7 | 3.10×10^-8^ |
| BP | GO:0043062 | extracellular structure organization | 7 | 3.16×10^-8^ |
| BP | GO:0045229 | external encapsulating structure organization | 7 | 3.30×10^-8^ |
| BP | GO:0072359 | circulatory system development | 6 | 4.32×10^-3^ |
| BP | GO:0036072 | direct ossification | 2 | 5.30×10^-3^ |
| CC | GO:0062023 | collagen-containing extracellular matrix | 8 | 9.22×10^-11^ |
| CC | GO:0031012 | extracellular matrix | 8 | 8.87×10^-10^ |
| CC | GO:0030312 | external encapsulating structure | 8 | 8.99×10^-10^ |
| CC | GO:0098644 | complex of collagen trimers | 3 | 1.17×10^-5^ |
| CC | GO:0071944 | cell periphery | 10 | 4.16×10^-4^ |
| MF | GO:0005201 | extracellular matrix structural constituent | 8 | 1.82×10^-13^ |
| MF | GO:0050840 | extracellular matrix binding | 5 | 5.76×10^-9^ |
| MF | GO:0005198 | structural molecule activity | 8 | 2.04×10^-7^ |
| MF | GO:0030020 | extracellular matrix structural constituent conferring tensile strength | 3 | 1.54×10^-4^ |
| MF | GO:0001968 | fibronectin binding | 2 | 1.69×10^-2^ |
| KEGG | KEGG:04512 | ECM-receptor interaction | 3 | 2.10×10^-3^ |
| KEGG | KEGG:04926 | Relaxin signaling pathway | 3 | 7.05×10^-3^ |
| KEGG | KEGG:04510 | Focal adhesion | 3 | 2.63×10^-2^ |

**Movie S1.**

Animation of the molecular mechanism of the hBMSC-LC interaction.

**References:**

[1] X. Jin, J. Zhuang, Z. Zhang, H. Guo, J. Tan, *J Colloid Interface Sci* **2015**, 443, 125.

[2] A. V. Paduraru, O. Oprea, A. M. Musuc, B. S. Vasile, F. Iordache, E. Andronescu, *Nanomaterials (Basel)* **2021**, 11.

[3] A. Person, H. Bocherens, J.-F. Saliège, F. Paris, V. Zeitoun, M. J. J. o. A. S. Gérard, **1995**, 22, 211.

[4] S. Park, J. O. Baker, M. E. Himmel, P. A. Parilla, D. K. Johnson, *Biotechnol Biofuels* **2010**, 3, 10.

[5] I. M. Low, *J Am Ceram Soc* **2005**, 87, 2125.

[6] O. A. Tertuliano, J. R. Greer, *Nat Mater* **2016**, 15, 1195.

[7] L. B. Gower, *Chem Rev* **2008**, 108, 4551.

[8] K. Nitiputri, Q. M. Ramasse, H. Autefage, C. M. McGilvery, S. Boonrungsiman, N. D. Evans, M. M. Stevens, A. E. Porter, *ACS Nano* **2016**, 10, 6826.

[9] W. J. Habraken, J. Tao, L. J. Brylka, H. Friedrich, L. Bertinetti, A. S. Schenk, A. Verch, V. Dmitrovic, P. H. Bomans, P. M. Frederik, J. Laven, P. van der Schoot, B. Aichmayer, G. de With, J. J. DeYoreo, N. A. Sommerdijk, *Nat Commun* **2013**, 4, 1507.

[10] a) E. Afgan, D. Baker, M. van den Beek, D. Blankenberg, D. Bouvier, M. Cech, J. Chilton, D. Clements, N. Coraor, C. Eberhard, B. Gruning, A. Guerler, J. Hillman-Jackson, G. Von Kuster, E. Rasche, N. Soranzo, N. Turaga, J. Taylor, A. Nekrutenko, J. Goecks, *Nucleic Acids Res* **2016**, 44, W3; b) D. Blankenberg, N. Coraor, G. Von Kuster, J. Taylor, A. Nekrutenko, T. Galaxy, *Database (Oxford)* **2011**, 2011, bar011.

[11] G. Zhou, O. Soufan, J. Ewald, R. E. W. Hancock, N. Basu, J. Xia, *Nucleic Acids Res* **2019**, 47, W234.

[12] U. Raudvere, L. Kolberg, I. Kuzmin, T. Arak, P. Adler, H. Peterson, J. Vilo, *Nucleic Acids Res* **2019**, 47, W191.

[13] L. J. Jensen, M. Kuhn, M. Stark, S. Chaffron, C. Creevey, J. Muller, T. Doerks, P. Julien, A. Roth, M. J. N. a. r. Simonovic, **2009**, 37, D412.

[14] D. Hong, H. X. Chen, H. Q. Yu, Y. Liang, C. Wang, Q. Q. Lian, H. T. Deng, R. S. Ge, *Exp Cell Res* **2010**, 316, 2291.

[15] C. Lee-Thedieck, P. Schertl, G. Klein, *Adv Drug Deliv Rev* **2022**, 181, 114069.

[16] J. Kruegel, N. Miosge, *Cellular and molecular life sciences : CMLS* **2010**, 67, 2879.

[17] G. S. Stein, J. B. Lian, *Endocr Rev* **1993**, 14, 424.

[18] D. F. Williams, *Bioact Mater* **2022**, 10, 306.

[19] C. Ye, W. Zhang, K. Hang, M. Chen, W. Hou, J. Chen, X. Chen, E. Chen, L. Tang, J. Lu, Q. Ding, G. Jiang, B. Hong, R. He, *Cell Death Dis* **2019**, 10, 753.

[20] J. F. Hastings, J. N. Skhinas, D. Fey, D. R. Croucher, T. R. Cox, *Br J Pharmacol* **2019**, 176, 82.

[21] H. Li, T. Li, J. Fan, T. Li, L. Fan, S. Wang, X. Weng, Q. Han, R. C. Zhao, *Cell Death Differ* **2015**, 22, 1935.

[22] F. Song, D. Jiang, T. Wang, Y. Wang, Y. Lou, Y. Zhang, H. Ma, Y. Kang, *Biomed Res Int* **2017**, 2017, 6027402.

[23] a) A. Wang, H. Yin, D. Liu, H. Wu, Y. Wada, M. Ren, Y. Xu, T. Jiang, X. Cheng, *Applied Surface Science* **2007**, 253, 3311; b) D. A. González-Martínez, G. González Ruíz, M. d. C. Luzardo Lorenzo, F. Bordallo-León, Y. Hechavarría Luna, Y. Cazañas Quintana, E. González-Martínez, K. León, A. González Palomo, J. A. García Artalejo, J. Moran-Mirabal, *ACS Applied Nano Materials* **2022**, 5, 6159.

[24] C. Zhang, C. Li, S. Huang, Z. Hou, Z. Cheng, P. Yang, C. Peng, J. Lin, *Biomaterials* **2010**, 31, 3374.

[25] T. Hoshino, M. Nakayama, S. Fujinami, T. Nakatani, Y. Kohmura, T. Kato, *Soft Matter* **2019**, 15, 3315.

[26] J. Tan, X. Jin, M. Chen, *Scientific Reports* **2019**, 9, 466.

[27] J. Tan, Y. Liu, J. Gong, X. Jin, C. Cheng, R. Zhang, M. Chen, *Acta Biomaterialia* **2020**, 116, 383.
